# Deciphering the significance of anoikis in bladder cancer and systematic analysis of S100A7 as a potential therapeutic target

**DOI:** 10.1186/s40001-024-01642-9

**Published:** 2024-01-12

**Authors:** Haoran Wang, Jianyong Liu, Runhua Tang, Jie Hu, Ming Liu, Jianye Wang, Jingwen Zhang, Huimin Hou

**Affiliations:** 1 Department of Urology, Institute of Geriatric Medicine, Beijing Hospital, National Center of Gerontology, Chinese Academy of Medical Sciences, No. 1 DaHua Road, Dong Dan, Beijing, 100730 China; 2 Graduate School of Peking Union Medical College, Chinese Academy of Medical Sciences, 9 DongDan SANTIAO, Beijing, 100730 China; 3 Fifth School of Clinical Medicine, Peking University, Beijing, China; 4 Department of Critical Care Medicine, The First Medical Centre, Chinese PLA General Hospital, Beijing, 100853 People’s Republic of China

## Abstract

**Background:**

Bladder cancer is an epidemic and life-threating urologic carcinoma. Anoikis is a unusual type of programmed cell death which plays a vital role in tumor survival, invasion and metastasis. Nevertheless, the relationship between anoikis and bladder cancer has not been understood thoroughly.

**Methods:**

We downloaded the transcriptome and clinical information of BLCA patients from TCGA and GEO databases. Then, we analyzed different expression of anoikis-related genes and established a prognostic model based on TCGA database by univariate Cox regression, lasso regression, and multivariate Cox regression. Then the Kaplan–Meier survival analysis and receiver operating characteristic (ROC) curves were performed. GEO database was used for external validation. BLCA patients in TCGA database were divided into two subgroups by non-negative matrix factorization (NMF) classification. Survival analysis, different gene expression, immune cell infiltration and drug sensitivity were calculated. Finally, we verified the function of S100A7 in two BLCA cell lines.

**Results:**

We developed a prognostic risk model based on three anoikis-related genes including TPM1, RAC3 and S100A7. The overall survival of BLCA patients in low-risk groups was significantly better than high-risk groups in training sets, test sets and external validation sets. Subsequently, the checkpoint and immune cell infiltration had significant difference between two groups. Then we identified two subtypes (C_A_ and C_B_) through NMF analysis and found CA had better OS and PFS than CB. Besides, the accuracy of risk model was verified by ROC analysis. Finally, we identified that knocking down S100A7 gene expression restrained the proliferation and invasion of bladder cancer cells.

**Conclusion:**

We established and validated a bladder cancer prognostic model consisting of three genes, which can effectively evaluate the prognosis of bladder cancer patients. Additionally, through cellular experiments, we demonstrated the significant role of S100A7 in the metastasis and invasion of bladder cancer, suggesting its potential as a novel target for future treatments.

**Supplementary Information:**

The online version contains supplementary material available at 10.1186/s40001-024-01642-9.

## Introduction

In the field of urological tumors, bladder cancer attracts the attention of numerous experts and scholars. Not only is it one of the most common malignancies, but it also has a high.

morbidity and mortality rate [1, 2]. According to statistics, more than 550,000 people are diagnosed with bladder cancer each year, and over 200,000 individuals succumb to the disease as a result [3].The majority of patients are diagnosed with bladder cancer through hospital examinations prompted by painless gross hematuria [4]. Following transurethral resection of bladder tumors, patients often require intravesical instillation chemotherapy [5]. However, there are still many cases of recurrence and even progression among patients [6]. Therefore, it becomes particularly important to explore biomarkers that can effectively evaluate patient prognosis.

Anoikis is a programmed cell death process that is distinct from apoptosis and autophagy [7]. It occurs when cells are unable to establish appropriate interactions with the surrounding extracellular matrix [8]. The extracellular matrix contains various cytokines that are critical for cell growth, motility, and angiogenesis [9]. Therefore, anoikis serves to maintain the integrity of tissues and organs by preventing abnormal cell-extracellular matrix interactions. Simultaneously, this also implies a close connection between anoikis and tumor metastasis and invasion. Anoikis resistance means that cancer cells can detach from the previous extracellular matrix and survive without undergoing cell death. They can then establish contact with a new extracellular matrix, leading to the implantation and metastasis of tumors in distant organs [10].

Bladder cancer is treated differently based on its various types. A study involving 172 patients with pure in situ carcinoma suggested that advanced age at diagnosis appears to be associated with an increased risk of recurrence and progression of pure bladder in situ carcinoma. Elderly patients may not respond effectively to Bacillus Calmette-Guérin (BCG) treatment [11]. For non-muscle-invasive bladder cancer, transurethral resection of the bladder tumor remains a primary treatment option. Postoperatively, the decision to undergo adjuvant therapy depends on the patient's pathological conditions. Treating muscle-invasive bladder cancer is often more challenging, and many patients require neoadjuvant therapy before surgery is performed upon detection. Not all patients tolerate platinum-based therapies. With the increasing research on immunotherapy, many patients receive immunotherapy before surgery, providing more opportunities for surgical interventions [12, 13]. As technologies like circulating tumor DNA (ctDNA) analysis emerge, more effective biomarkers will be identified to guide treatment decisions and assess treatment outcomes [14].

Platinum-based chemotherapy is a crucial therapeutic approach for bladder cancer, yet the survival benefits for patients are relatively modest, with only about 15% achieving long-term relief [15]. Consequently, with the advent of immune checkpoint inhibitors, an increasing number of immunotherapeutic drugs are being developed, and more treatment targets are being identified [16]. Fibroblast growth factor receptor (FGFR) has emerged as a potential therapeutic target, entering clinical trials and bringing new hope for advanced cancer patients [17]. In this article, we delve into the crucial role of anoikis resistance in bladder cancer, examining its interplay with immune checkpoints and the immune microenvironment. This exploration provides new theoretical and experimental foundations for the development of novel drugs.

## Materials and methods

### Data collection

The transcriptomic data and clinical information of bladder cancer patients were downloaded from TCGA and GEO databases. The TCGA database included 19 normal individuals and 412 tumor patients. Through literature search and Genecards database ((https://www.genecards.org/) exploration, a total of 652 anoikis-related genes were identified. Genes exhibiting a false discovery rate (FDR) less than 0.05 and a log fold change (log FC) greater than 1 were defined as differentially expressed genes.

A total of 281 bladder cancer patients’ and 68 normal individuals' information were downloaded from the GEO database (GSE13507 and GSE31684). These data were utilized as the validation cohort for the prognostic assessment model. Additionally, the IMvigor210 cohort information was also obtained to evaluate the efficacy of immune therapy in different risk groups of patients.

### Construction of prognostic signature based on anoikis -related genes

First, we merged the expression matrix of differentially expressed genes with the patients' survival data. Subsequently, the patients in the TCGA database were randomly divided into control and validation groups in a 1:1 ratio. Univariate regression analysis was performed to identify genes associated with prognosis, and visualization was carried out using R packages “survival” and “forest plot”. CNV frequency was also conducted. Next, lasso regression analysis and multivariable regression analysis were employed to optimize the prognostic risk model. The risk score for each sample was calculated using the following formula, where “coef” represents the regression coefficients obtained from the multivariate Cox regression analysis, and “X” represents the expression levels of risk genes.

$$Risk\,score = \sum\nolimits_{{i = 1}}^{n} {\left( {Coefi \times Xi} \right)}$$

The patients were divided into low-risk group (LRG) and high-risk group (HRG) based on the median risk score. The survival status, expression levels of risk genes, and risk scores of patients in different risk groups were visualized. The overall survival rate and progression-free survival (PFS) were depicted using Kaplan–Meier curves for different risk groups.

### Establishment of the nomogram

We quantified different risk factors to assess patient prognosis. Receiver operating characteristic (ROC) analysis was performed on the risk score and clinical variables at 1, 3, and 5 years to determine the optimal prognostic indicators. The “rms” package in R was utilized to establish the analysis framework, and the visualization was accomplished using the “regplot” function. The model's consistency was evaluated using calibration curves.

### Consensus clustering analysis

Based on the genes selected from the risk model, we performed unsupervised consensus clustering on the patients in the TCGA cohort using the R package “ConsensusClusterPlus” [18]. The clustering results were validated using principal component analysis (PCA). Kaplan–Meier survival curves were used to visualize the overall survival (OS) rates of patients in different clusters.

### GSVA and ssGSEA analysis

Using the R package "GSVA," we analyzed the KEGG pathways between different clusters to investigate the biological process differences among subgroups [19]. The ssGSEA algorithm was employed to study the relationship of immune cell infiltration between different subgroups. The R package "ggplot2" was used to visualize the infiltration of immune cells in different subgroups.

### Functional enrichment analysis

Gene Ontology (GO) and Kyoto Encyclopedia of Genes and Genomes (KEGG) analysis were employed to explore functional analysis (adjusted p value < 0.05). Besides, based on the molecular feature databases such as KEGG and HALLMARK gene sets, we implemented Gene Set Enrichment Analysis (GSEA) to identify molecular and biological differences between different cohorts [20] (https://www.gsea-msigdb.org/gsea/msigdb).

### The correlation between risk stratification and clinical variables

The distribution of clinical variables, including age, gender, grade, stage, and TNM staging, among different groups of patients can be visualized. Additionally, the gene expression from the risk model for different patients can be presented using a heatmap. Further analysis was conducted focusing on T stage and stage, and the proportion of patients in each clinical variable subgroup can be displayed.

### Immune cell infiltration, immune microenvironment, and genetic alterations analysis

The ESTIMATE algorithm can be used to estimate the immune cell infiltration in different patient groups. Information on somatic cell alterations can be downloaded from the TCGA database. The top 15 genes with the highest mutation frequency can be visualized using R package “Maftools”. Based on the occurrence risk of tumor mutational burden (TMB) and sample risk grouping, the patients can be classified into four major categories, and their survival rates can be calculated to construct survival curves. The correlation between risk score and immune cell infiltration can be analyzed using various methods such as XCELL, TIMER, QUANTISEQ, MCP COUNTER, EPIC, CIBERSORT, and CIBERSORT-ABS. These methods can provide insights into the relationship between risk score and the abundance or proportions of immune infiltrating cells in the tumor microenvironment.

### Prediction of immunotherapy response and drug sensitivity

We assessed the expression of immune checkpoint-related genes in different risk groups of patients. Furthermore, we evaluated the tumor immunogenicity and response to different immune checkpoint drugs in high-risk and low-risk samples based on the Immune Phenotype Score (IPS). Using the R package “prophetiC,” we predicted the sensitivity of patients in different risk groups to different drugs, providing new insights and approaches for personalized treatment of bladder cancer.

### Cell culture

We selected UMUC-3 and T24 for further experimental validation. Cells were cultured in DMEM and RPMI-1640 medium plus 10% fetal bovine serum respectively, at 37 °C in a 5% CO2 environment. Subsequently, two siRNAs were applied to knock down expression level of S100A7. The siRNA sequences were as follows: si-S100A7-1: 5′-CCAGACGUGAUGACAAGAUTT-3′, si-S100A7-2: 5′-5′-CAAAUUACCUCGCCGAUGUTT-3′.

### Cell viability assay

Cells were transfected in a six-well plate. After transfection, the transfected cells were collected and counted. Approximately 3000 cells were seeded into a 96-well plate, and cell viability at different time points was observed using the CCK-8 assay, following the manufacturer’s recommended protocol.

### EdU analysis

T24 and UMUC3 cells were seeded into a glass bottom cell culture dish (NEST), and siRNA transfection was performed. After 48 h of continued incubation, the culture medium was removed, and EdU was added for an additional 2 h. The cells were then stained using paraformaldehyde according to the manufacturer's instructions for subsequent staining. Imaging was performed using a confocal microscope, and cell counting was conducted using ImageJ for analysis.

### Wound healing assay

T24 and UMUC3 cells were seeded into a 6-well plate and subjected to siRNA transfection. When the cells reached approximately 100% confluence, a wound was created in the cell monolayer using a 10µL pipette tip. The medium was then replaced with serum-free culture medium and incubated in a cell culture incubator. Images were captured at 0 and 48 h under a microscope to record the wound healing process. ImageJ was used to analyze the extent of wound closure.

### Transwell assay

Transfected cells were collected and subsequently centrifuged. After centrifugation, cells were resuspended in serum-free RPMI-1640 or DMEM medium and cultured on the upper surface of Transwell chambers, while the lower chamber contained medium supplemented with FBS. After 24 h, residual cells on the upper membrane surface were gently wiped off using a cotton swab. Cells adhering to the lower membrane were fixed with 4% paraformaldehyde and stained with crystal violet. Subsequently, cells were photographed using an optical microscope. Finally, cell analysis was performed using ImageJ.

## Results

### Expression of anoikis-related genes and construction of a prognostic model

**Fig. 1:**
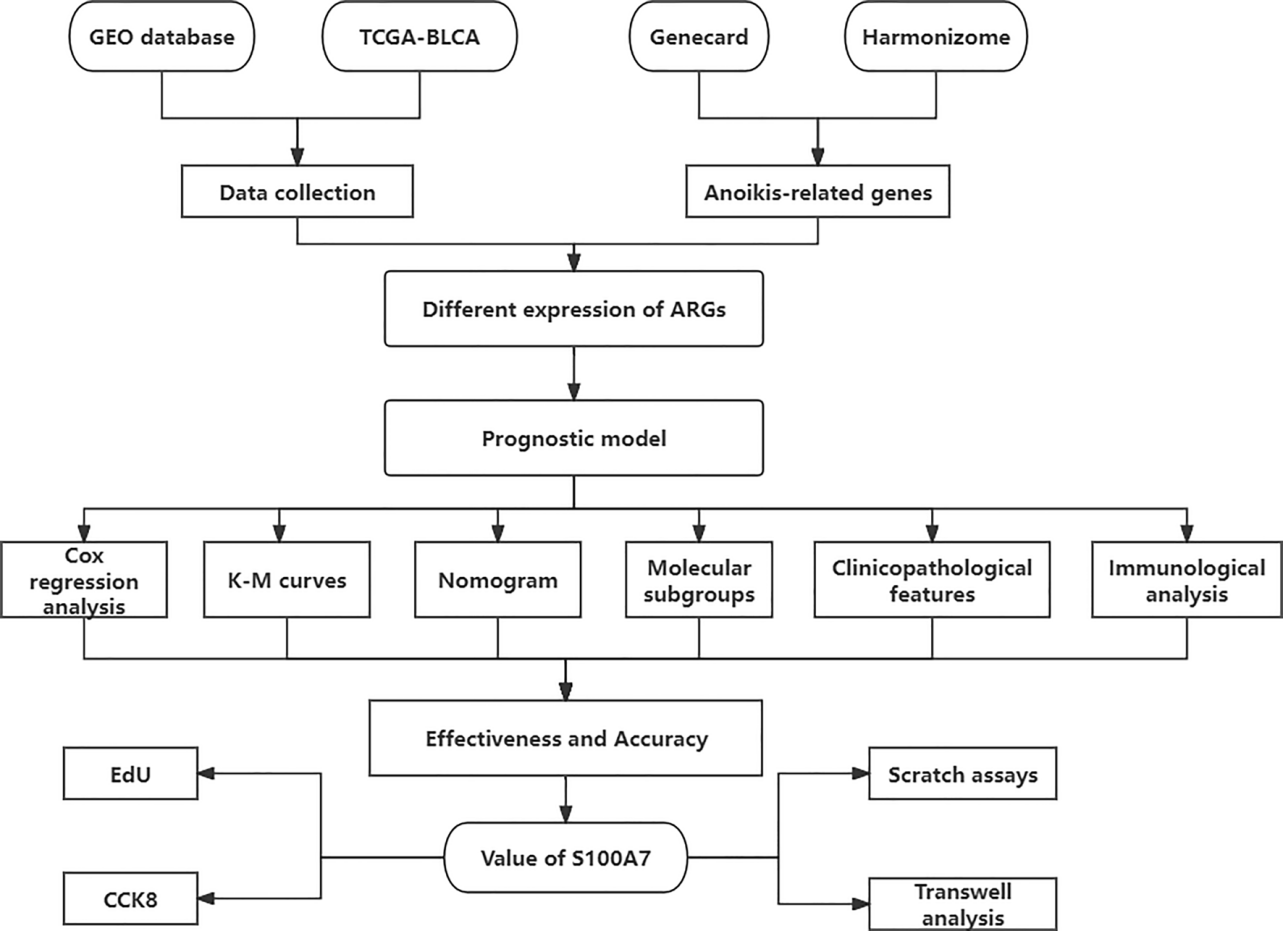
The flowchart illustrating the methodology of this study

The overall experimental design workflow for this study is illustrated in Fig. 1.

**Fig. 2:**
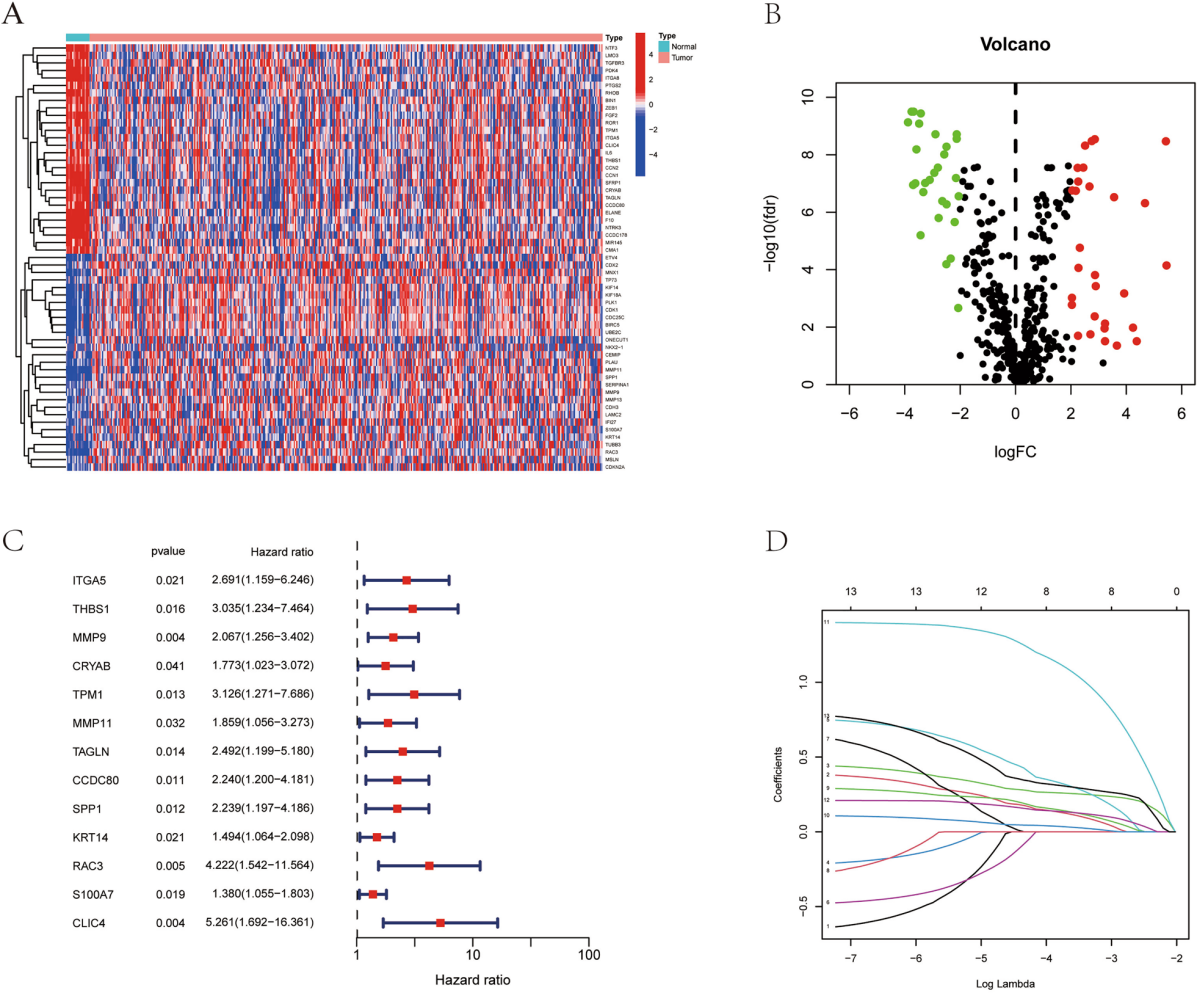
Establishment of prognostic model for BCa. **A** The heatmap of differentially expressed ARGs. **B** The volcano plot of differentially expressed ARGs. **C** Cox regression of ARGs associated with prognosis. **D** Cross-validation for variable selection in LASSO analysis

We initiated our study by retrieving and collecting anoikis-related genes from the Genecard and Harmonizome databases. In the Genecard database, we selected genes with a correlation greater than 0.4. Subsequently, we defined genes with log FC > 1 and Fdr < 0.05 as differentially expressed genes (DEGs) and identified DEGs related to anoikis in normal individuals and bladder cancer patients. A total of 57 DEGs were identified, consisting of 29 upregulated and 28 downregulated genes. We then selected the top 50 DEGs with the largest differences to generate a heatmap and volcano plot (Fig. 2A, B).

Following this, we subjected these DEGs to univariate Cox regression analysis and identified 13 genes that were associated with bladder cancer (Fig. 2C). To construct a predictive model and assess its accuracy, we randomly divided the TCGA data into training and internal validation sets in a 1:1 ratio, The clinical information between the two patient groups showed no statistically significant differences (Additional file 1: Table S1). Using LASSO Cox regression analysis, we established a prognostic gene model for bladder cancer patients as shown in Fig. 2D and Additional file 1: Figure S1A. Ultimately, we constructed the prognostic model using TPM1, RAC3, and S100A7, and calculated the risk scores as formula mentioned before.

### Internal validation and external validation of the prognostic model

**Fig. 3:**
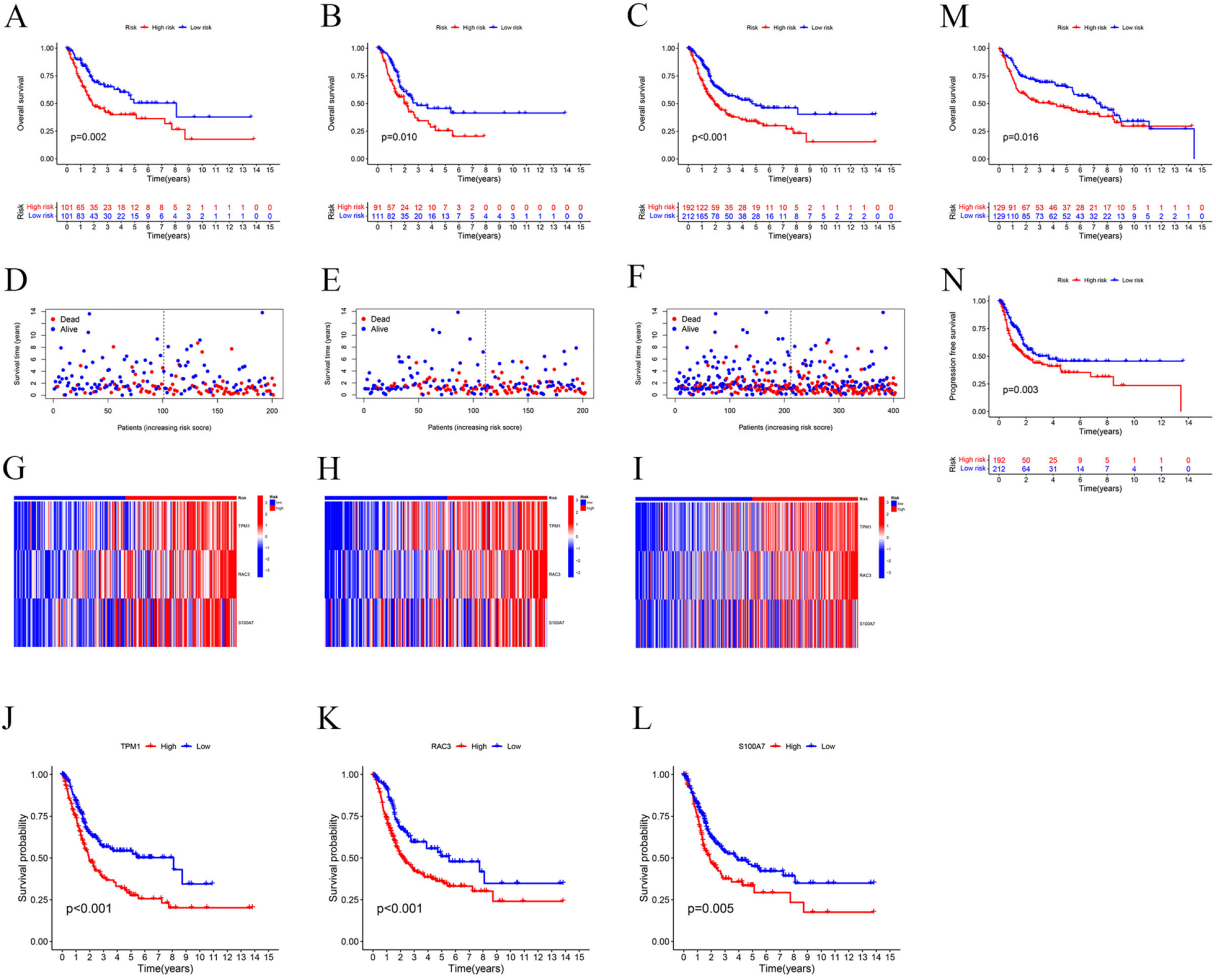
Prognosis of the risk model in different cohorts. The Kaplan-Meier curves, survival status and the heatmap of 3 ARGs in train cohort **A**, **D**, **G**, test cohort **B**,** E**,** H** and all TCGA cohort **C**, **F**,** I**. **J–L** Impact of each model gene on patient survival. **M** The Kaplan-Meier curve in GEO cohort. **N** The Kaplan-Meier curve of PFS.

In TCGA, patients in both the training and validation sets were stratified into high-risk and low-risk groups using the risk model. As shown in Fig. 3A–C, patients in the high-risk group exhibited poorer prognoses in training set (p = 0.002), validation set (p = 0.01) and all TCGA set (p < 0.001). Furthermore, from the scatter plot and heatmap of patient risk scores, it is evident that the high-risk group has a higher number of deaths compared to the low-risk group and all three genes are highly expressed in the high-risk group. (Fig. 3D–I). To further validate the accuracy of our model, we separately investigated the impact of the three genes on the prognosis of bladder cancer patients. Survival curves revealed that high expression of all three genes was associated with adverse outcomes in Fig. 3J–L (TPM1 p < 0.001; RAC3 p < 0.001; S100A7 p = 0.005).

We merged GSE13507 and GSE31684 datasets into a single dataset for external validation. Using the risk model calculation formula, we stratified bladder cancer patients from the GEO dataset into high-risk and low-risk groups. Survival curve analysis in Fig. 3M revealed that patients in the high-risk group had significantly worse prognoses (p = 0.016), consistent with our findings in the TCGA database. Additionally, we investigated the impact of the risk model on Progression-Free Survival (PFS) in bladder cancer patients using the TCGA database. The results in Fig. 3N showed that patients in the high-risk group had shorter PFS (p = 0.003).

### Determination of independent prognostic indicators

**Fig. 4:**
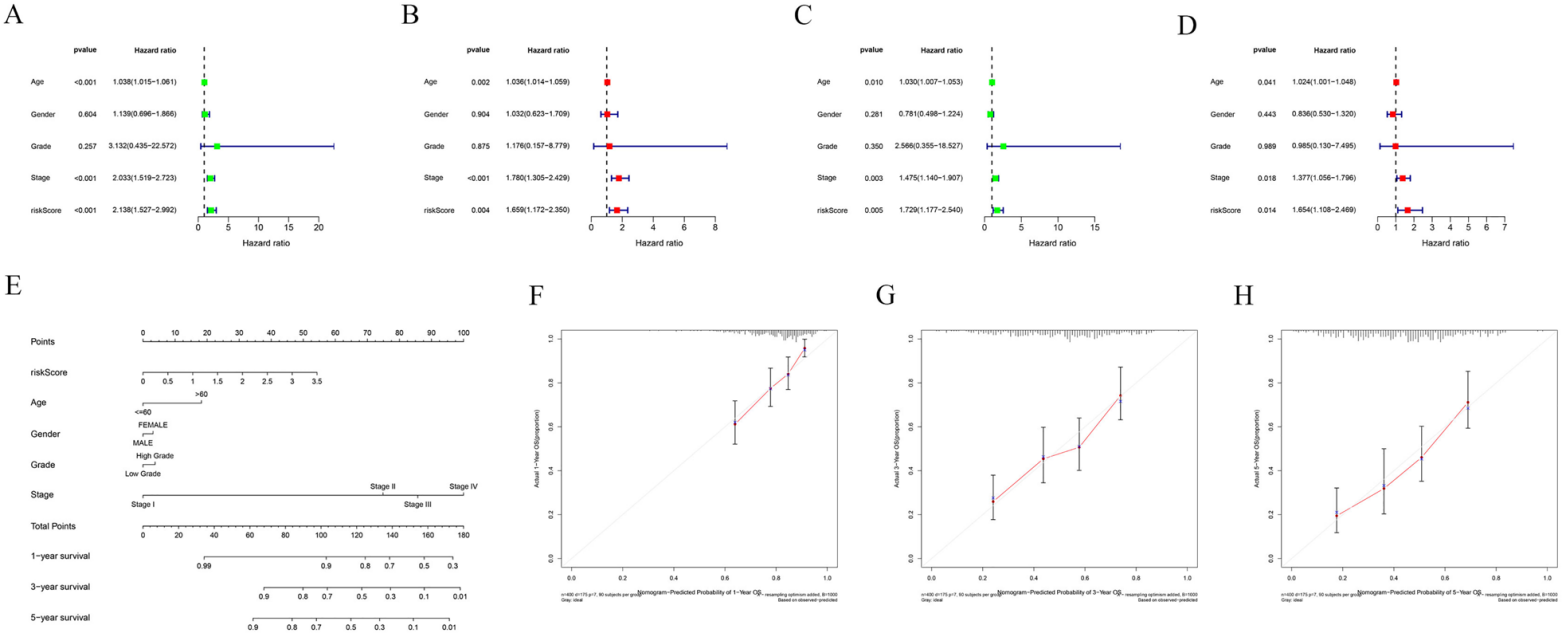
Determination of independent prognostic factors and nomogram. **A**, **B** The univariate and multivariate Cox regression analyses in training set. **C**, **D** The univariate and multivariate Cox regression analyses in test set. **E** The establish of nomogram. **F–H** The calibration curve which illustrates the consistency between actual outcomes and predictions

We verified whether our prognostic model can serve as an independent prognostic factor for bladder cancer patients by conducting univariate Cox regression and multivariate Cox regression analyses. We conducted separate analyses for the training set, validation set, and the entire TCGA cohort. In the training set shown in Fig. 4A, B, both univariate and multivariate Cox regression analyses indicated that age, stage, and the risk model were independent prognostic factors. Similar results were obtained in the training set (Fig. 4C, D) and the entire TCGA cohort (Additional file 1: Figure S1B, C). The Additional file S1: Fig. 2A–F display the impact of different risk factors on survival. This suggests that our prognostic risk model can be applied as an independent prognostic factor in bladder cancer patients.

### Construction of nomogram

To provide more individualized guidance and prognostic assessment for bladder cancer patients, we developed a nomogram. This nomogram shown in Fig. 4E incorporates our prognostic model along with other clinical indicators to assign scores to different bladder cancer patients. These scores are then used to predict the 1-year, 3-year, and 5-year survival rates of the patients. To validate the accuracy of the nomogram, we introduced a calibration curve (Fig. 4F–H). The results showed a close alignment between our model's predictions and the actual survival rates of the patients, indicating that our nomogram is highly accurate and can provide valuable guidance for clinical treatment.

### Clustering and analysis of molecular subgroups

**Fig. 5:**
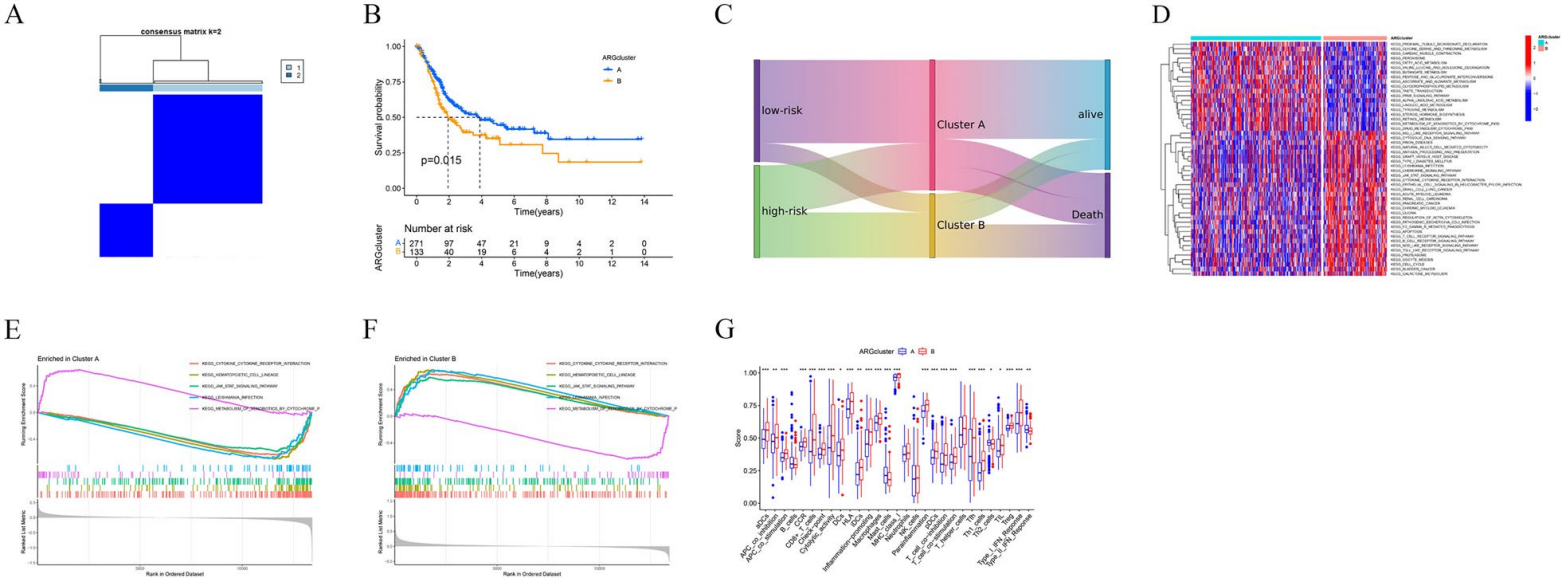
Subgroups determination and analysis. **A** The discrimination is most pronounced when the k = 2. **B** The Kaplan–Meier curve of OS between two subgroups. **C** Sankey diagram illustrating the relationship between patient high/low-risk grouping, subgroups, and prognosis. **D** GSVA between two clusters. **E–F** GSEA between two clusters. **G** Different immune cell scores between two clusters

We performed cluster analysis of bladder cancer patients from TCGA based on the model genes using the ‘ConsensusClusterPlus’ R package, which resulted in the classification of patients into two groups, A and B (Fig. 5A). Survival analysis shown in Fig. 5B revealed that bladder cancer patients in Group B had significantly worse survival outcomes compared to Group A (p = 0.015). The Sankey diagram results show that patients belonging to the low-risk group and Cluster A have a better prognosis (Fig. 5C). Subsequently, we conducted principal component analysis (PCA) and found clear differentiation between the two groups (Additional file 1: Figure S3A, B).

Following the identification of survival differences between the two groups, we applied Gene Set Variation Analysis (GSVA) to further analyze the patients (Fig. 5D). The results indicated significant pathway differences between the two groups, with the top 50 pathways presented in the form of a heatmap. Gene Set Enrichment Analysis (GSEA) was also applied, revealing that the 'metabolism of xenobiotics by cytochrome P450' pathway was significantly activated in Group A patients (Fig. 5E). In contrast, pathways activated in Group B patients included ‘Cytokine–receptor interactions,’ ‘Hematopoietic cell lineage,’ ‘JAK/STAT pathway,’ and 'Leishmania infection.' (Fig. 5F) The activation of these pathways in Group B patients may be associated with adverse prognostic outcomes. Meanwhile, there were significant differences in immune cell scores among patients in different subgroups (Fig. 5G).

### Correlations between clinicopathological features in different groups

**Fig. 6:**
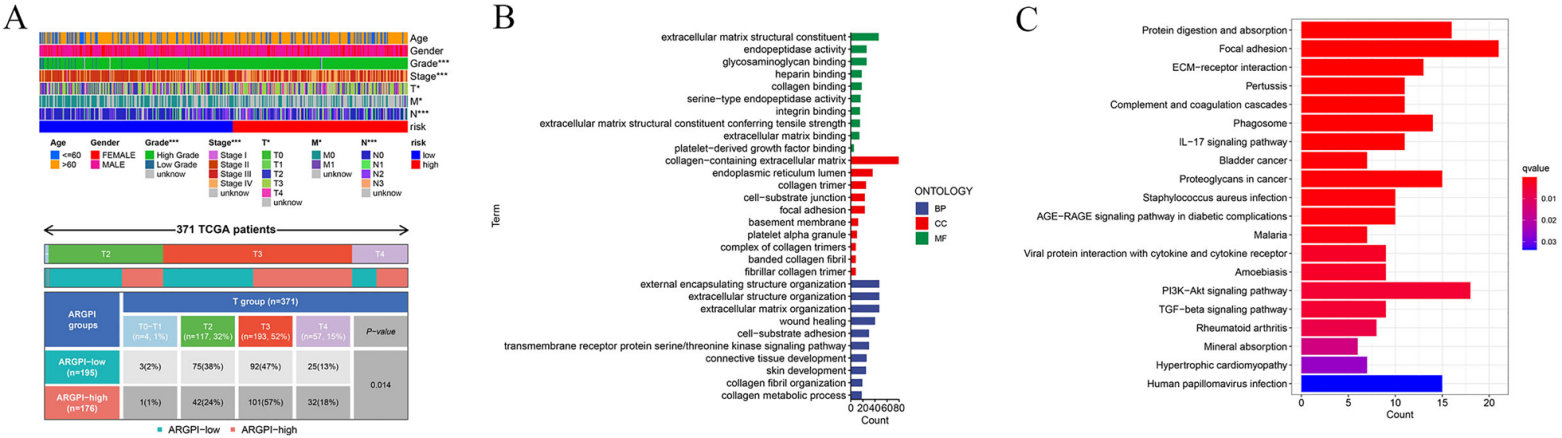
Different clinical, pathological characteristics and richness analysis. **A** The Clinical correlation analysis. **B** The different richness of molecular biological processes (BP), cellular components (CC), and molecular functions (MF) through GO analysis. **C** The different richness pathways via KEGG analysis

We utilized a heatmap to assess the clinical-pathological characteristics of patients in different prognostic risk groups, as shown in Fig. 6A. The results indicated that there were no statistically significant differences in age and gender between the high-risk and low-risk groups. However, significant differences were observed in tumor stage, grade, and TNM classification between the two groups. Subsequently, we conducted further analysis of tumor stage (Additional file 1: Figure S4) and T classification, revealing that high-risk group patients had significantly higher tumor stage (p = 0.001), and there was a higher proportion of T3 and T4 patients in the high-risk group with statistical significance (p = 0.014).

### Functional analysis between two groups

To further investigate the differences between the high and low-risk groups, we employed the “limma” package with criteria of |logFC|> 1 and fdr < 0.05. This analysis identified a total of 326 differentially expressed genes, of which 61 genes were upregulated in the low-risk group, while 265 genes were upregulated in the high-risk group (Additional file 1: Table S2).

Gene Ontology (GO) analysis shown in Fig. 6B revealed that in the Biological Process (BP) category, differentially expressed genes were primarily associated with extracellular matrix organization, extracellular structure organization, and external encapsulating structure organization. In the Cellular Component (CC) category, these genes were mainly related to the collagen-containing extracellular matrix. In the Molecular Function (MF) category, enriched differentially expressed genes were primarily associated with extracellular matrix structural constituents. Additionally, KEGG pathway enrichment analysis indicated a close association of these differentially expressed genes with the Focal adhesion and PI3K-Akt signaling pathways (Fig. 6C).

### Differences in immune cell infiltration and immune checkpoint expression

**Fig. 7:**
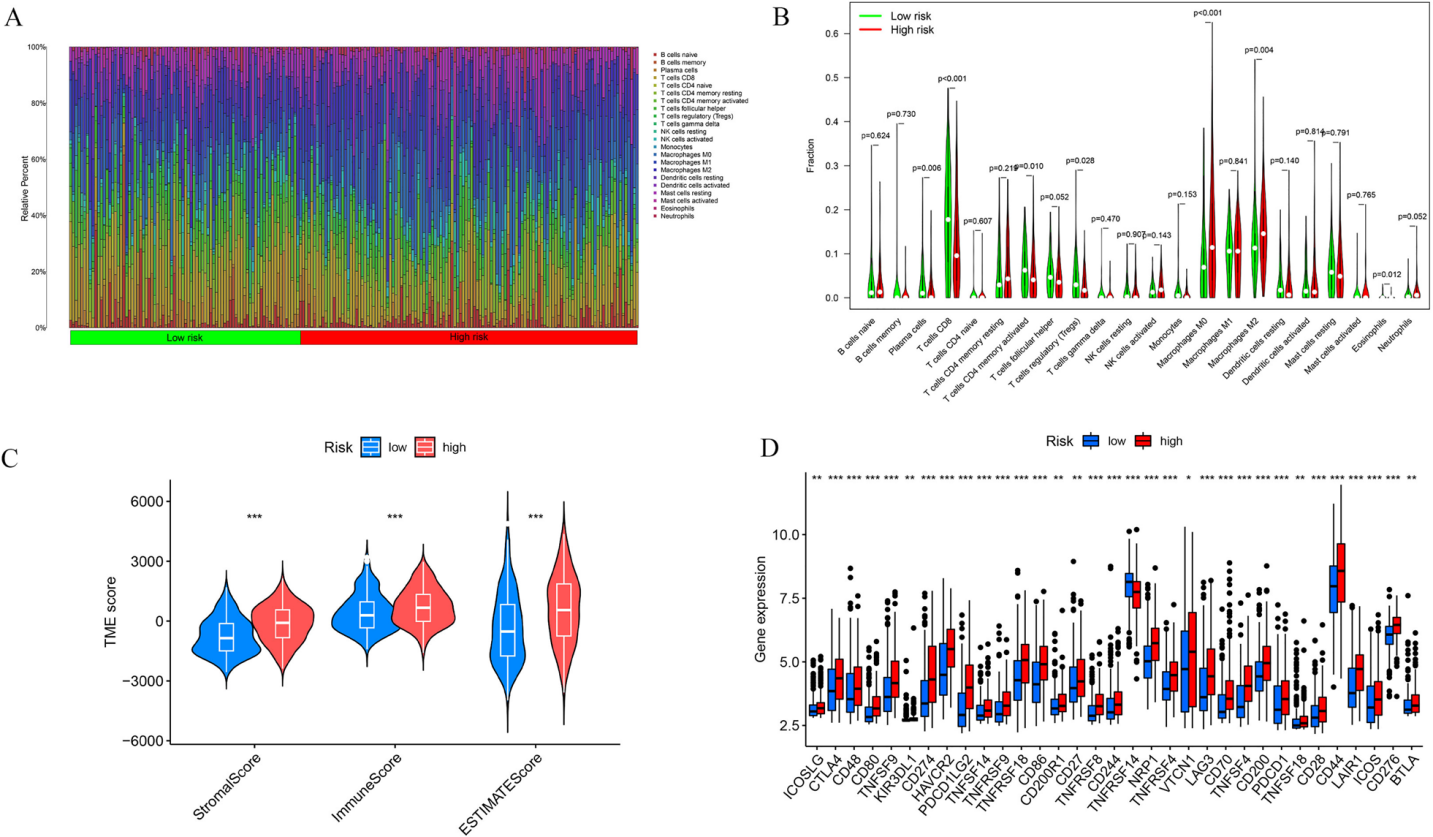
Analysis of different immune cell infiltrations. **A**, **B** The different immune cell infiltrations between high and low risk groups. **C** The estimate score between high and low risk groups. **D** The different expression of immune checkpoint markers

To investigate whether there are differences in immune cell infiltration between high-risk and low-risk group patients, we conducted an analysis using CIBERSORT. As depicted in the bar charts and violin plots in Fig. 7A, B, there were notable differences in the infiltration of various immune cells between the two groups. Plasma cells, T cells CD8, T cells CD4 memory activated, T cells regulatory (Tregs), and Eosinophils were significantly increased in the low-risk group, while Macrophages M0 and Macrophages M2 were markedly elevated in the high-risk group. This suggests that increased infiltration of macrophages, especially the M2 subtype, might be associated with adverse prognostic outcomes, although further experiments are needed to validate this. Subsequent correlation analysis revealed that T cells CD4 memory resting, neutrophils, macrophages M2, and macrophages M0 were positively correlated with the risk score. The correlations between different immune cells, model genes, and risk scores are detailed in the Additional file 1: Figure S5.

The ESTIMATE results indicate that patients in the high-risk group exhibit higher stromal, immune, and ESTIMATE scores compared to the low-risk group (Fig. 7C). Given the significant differences in immune cell infiltration between different groups of patients, we also analyzed the expression of immune checkpoint markers in these groups (Fig. 7D). The results showed that the majority of immune checkpoint markers, such as CTLA4, PDCD1, were highly expressed in high-risk group patients. Interestingly, only TNFRSF14 exhibited a negative correlation with the risk score, indicating higher expression in low-risk group patients.

### Tumor mutation analysis, immunotherapy and drug sensitivity between two groups

**Fig. 8:**
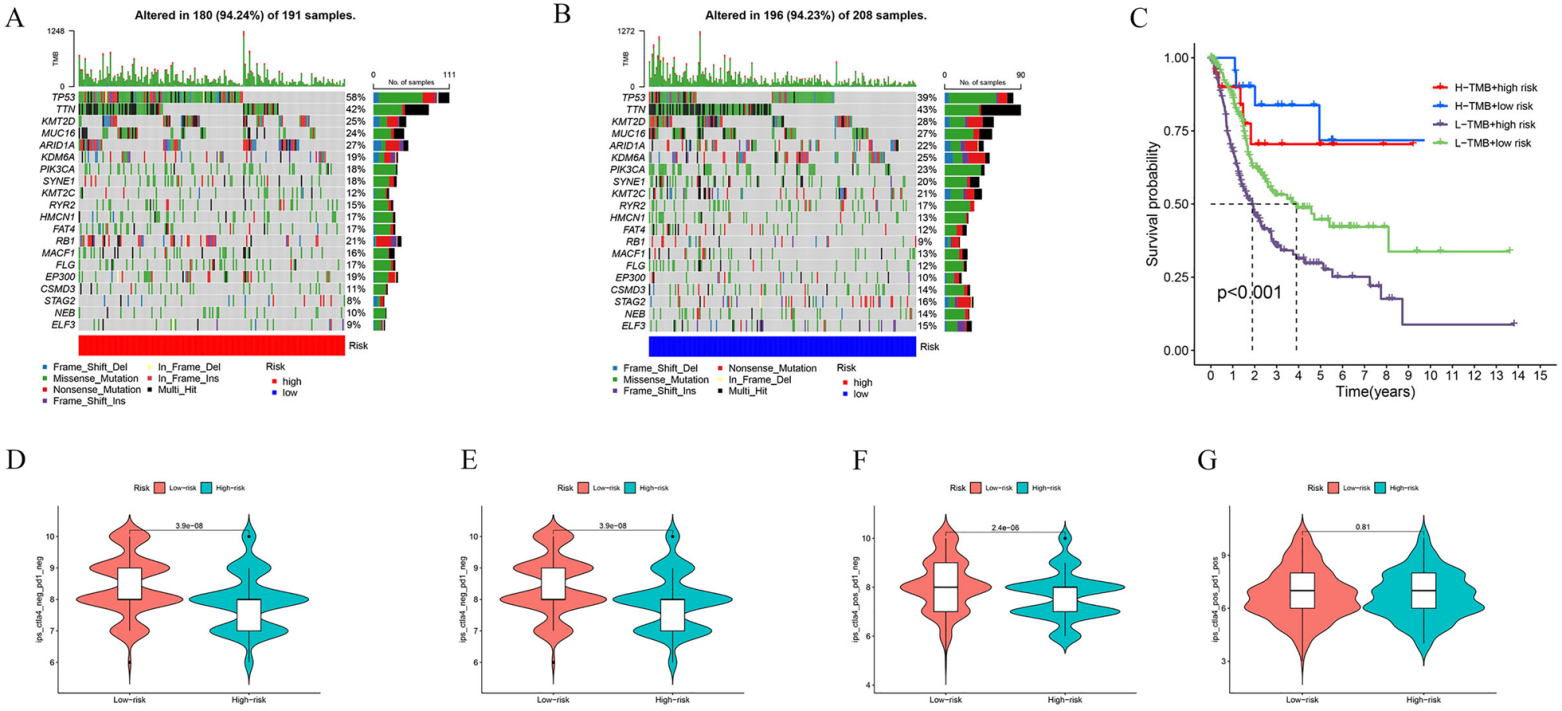
Somatic mutation characteristics and immunotherapy analysis. **A**, **B** The different somatic mutation characteristics in different risk groups. **C** The Kaplan–Meier survival curves between the four different groups. **D–G** The different IPS scores in different groups

The tumor mutation analysis results indicate significant differences in gene mutation profiles between the high-risk and low-risk groups in Fig. 8A, B. In the high-risk group, TP53 had the highest mutation frequency at 58%, followed by TTN at 42%. Conversely, in the low-risk group, the situation was markedly different, with TTN having the highest mutation frequency at 43%, and TP53 mutation frequency decreasing to 39%. This suggests that the high mutation rate of TP53 may be associated with a high-risk score and poor prognosis.

Although there is no difference in tumor mutation burden (TMB) between the high-risk and low-risk groups, survival analysis results indicate that high-risk patients with low TMB have a poorer prognosis (Fig. 8C). In the low-risk group, both IPS and IPS-CTLA4 scores are comparatively higher (Fig. 8D–G). Drug sensitivity is explained in the Additional file 1.

### Determine the key role of S100A7

**Fig. 9:**
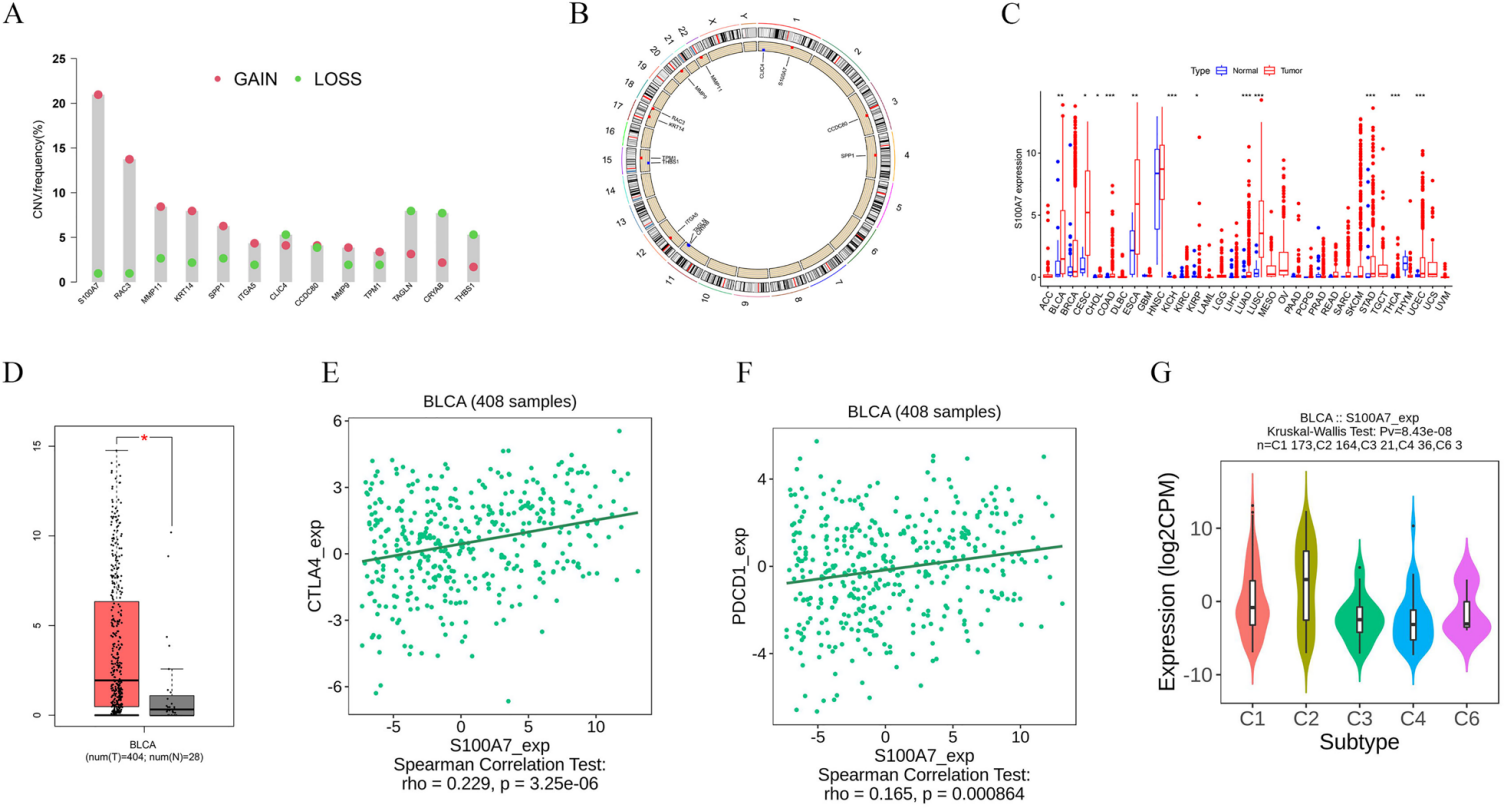
The pivotal role of S100A7. **A** Copy number variations of different prognostic genes. **B** Circus plot illustrating the mutation positions of different prognostic genes. **C** The different expression of S100A7 in different cancers. **D** The expression of S100A7 between normal and tumor tissues. **E**, **F** The correlation among the expressions of CTLA4, PDCD1, and S100A7. **G** The relationship between different immune subtypes and S100A7

First, we performed copy number variation (CNV) analysis on the 13 pathogenic genes identified through single-factor Cox regression. As shown in Fig. 9A, we found that S100A7 had the highest copy number gain. Additionally, we used a circular plot to label the positions of these genes on the chromosome (Fig. 9B). Pan-cancer analysis reveals a significant upregulation of S100A7 expression in various cancers, including bladder cancer (Fig. 9C). Furthermore, analysis in the GEPIA database revealed that S100A7 is highly expressed in tumor cells (Fig. 9D). TISIDB database analysis indicated a positive and statistically significant correlation between S100A7 and immune checkpoint-related genes CTLA4 and PDCD1 (Fig. 9E, F). Figure 9G indicated that there is also a strong connection between the immune microenvironment and S100A7. Bladder cancer patients were classified into six immune subtypes, which may assist in categorizing different types of bladder cancer based on immune responses. Based on these results, we speculate that S100A7 may have a relationship with the immune microenvironment, which in turn affects tumor development and treatment response.

To further explore the regulatory mechanisms involved, we identified seven potential transcription factors (TFs) that could potentially regulate the expression of S100A7 (Table 1). Differential analysis revealed that only SATB1 exhibited significant differences between normal and tumor patients (Additional file 1: Table S3). This suggests that SATB1 may play a crucial role in the regulation of S100A7, although further experimental validation is needed. In conclusion, S100A7 plays a significant role in bladder cancer, and as such, we have selected it for further experimental validation and analysis.

**Table 1:** The potential transcription factors for S100A7

Gene	TF
S100A7	ZNF750
	GRHL1
	STAT1
	SATB1
	GRHL3
	FOXN1
	ASCL1

### Cellular Experiments Validating the Role of S100A7

**Fig. 10:**
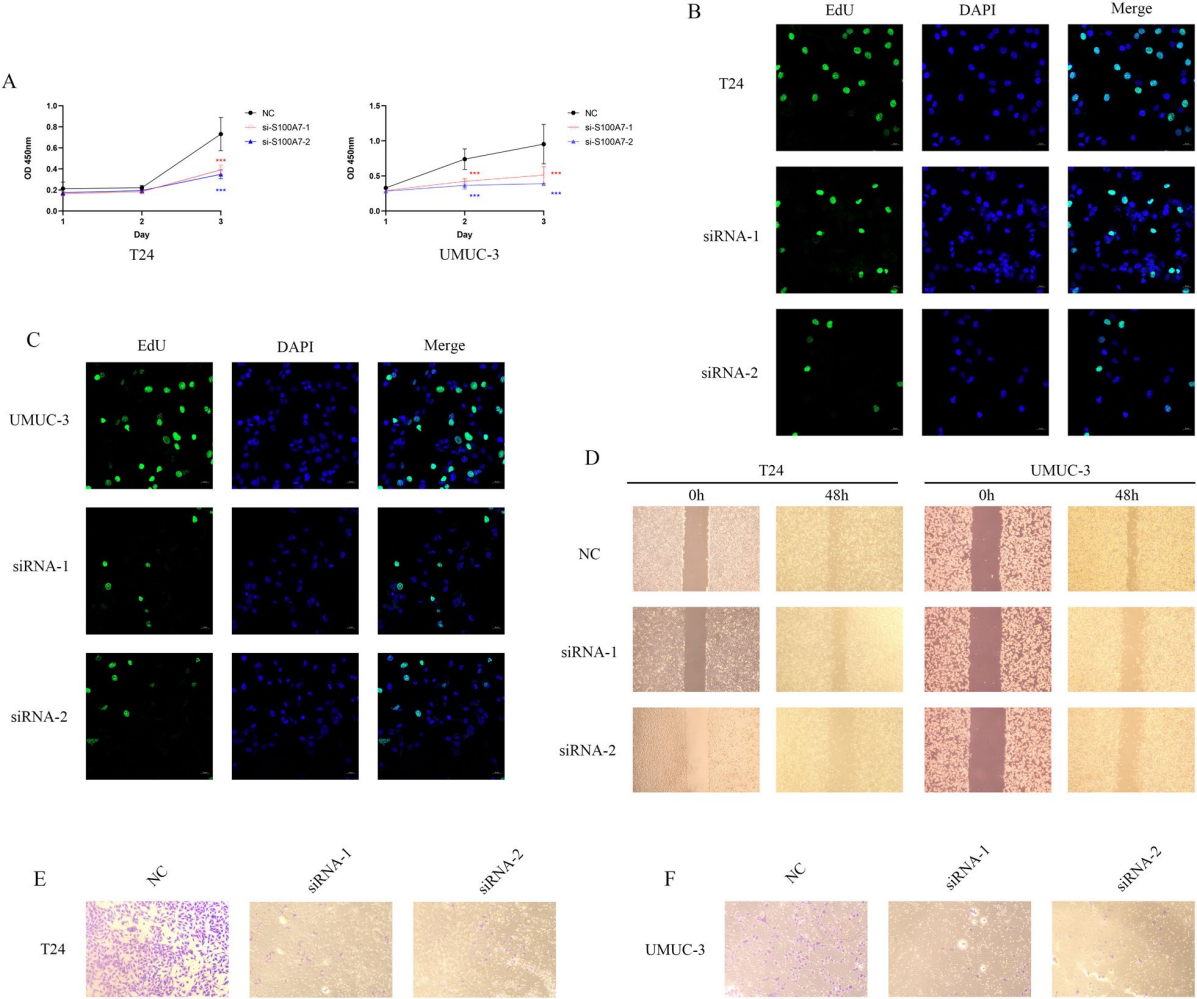
Cellular experiments of S100A7. **A** CCK8 experiments in T24 and UMUC-3 cell lines. **B**, **C** EdU analysis in T24 and UMUC-3 cell lines. **D** Wound healing experiments in two bladder cancer cell lines. **E** Transwell experiments in two bladder cancer cell lines

We selected two small interfering RNAs (siRNAs) for transfection into T24 and UMUC-3 bladder cancer cell lines to validate the impact of S100A7 on tumor cells. Subsequently, we conducted CCK-8 and EdU assays on transfected cells. The results in Fig. 10A revealed that the proliferation capacity of cells was notably reduced after S100A7 knockdown. The EdU assay shown in Fig. 10B, C also indicated a significant decrease in the percentage of green fluorescent-positive cells after transfection. Scratch assays in Fig. 10D demonstrated that the migration ability of tumor cells was substantially decreased upon S100A7 silencing. Transwell experiments further confirmed that, compared to non-knockdown cells, S100A7-silenced cells exhibited significantly reduced migration capabilities (Fig. 10E, F). These in vitro experimental findings underscore the significant role of S100A7 in tumor cell proliferation and migration, suggesting its potential as a therapeutic target in future bladder cancer treatments.

## Discussion

Bladder cancer is a highly recurrent and invasive tumor, with a very low 5-year overall survival rate for patients with metastatic bladder cancer [21]. Anoikis, a form of programmed cell death, prevents detached cells from adhering to inappropriate locations and developing abnormally, making it a crucial cellular defense mechanism [22]. It can be triggered through various intracellular pathways, including DNA damage and endoplasmic reticulum stress, as well as extrinsic pathways [23, 24]. Some researchers consider anoikis induction as a potential hallmark of cancer cells, as it can promote tumor invasion, metastasis, and resistance to therapies [25–27]. Therefore, we established a prognostic risk model related to anoikis to predict the prognosis of bladder cancer patients and aid in the development of potential therapeutic strategies. Furthermore, we conducted in-depth research into the role of anoikis-related gene S100A7 in bladder cancer, particularly its impact on cell proliferation and invasion.

In this study, we initiated an analysis of anoikis-related genes in normal and bladder cancer tissues. The results revealed elevated expression of multiple anoikis-related genes in bladder cancer, consistent with previous research and indicating a potential association between anoikis and bladder cancer prognosis [28, 29], suggesting therapeutic significance. Subsequently, employing COX regression and LASSO regression analyses, we established a prognostic model for anoikis-related outcomes involving three genes (TPM1, RAC3, S100A7). Further analysis of this model demonstrated significant discriminative ability in the TCGA internal training set (p = 0.002), validation set (p = 0.01), overall TCGA cohort (p < 0.001), and external GEO validation cohort (p = 0.016). These findings underscored the substantial impact of anoikis on the overall survival of cancer patients. Indeed, the role of anoikis has been extensively investigated in patients with hepatocellular carcinoma [30], prostate cancer [31, 32], gastric cancer [33, 34], and other malignancies [35–37]. This study aims to further contribute to the understanding of the role of anoikis in bladder cancer patients, thereby addressing a gap in the current research on this topic in bladder cancer.

The TPM1 gene is considered a member of the tumor-related protein family, originally known as the tropomyosin family [38]. It regulates tumor-specific variations by modulating stress fibers and modifying the actin cytoskeleton through the aggregation of actin filaments [39]. The correlation between RAC3 and poor prognosis in bladder cancer has been established [40]. Cheng and his colleagues found that elevated RAC3 expression is associated with adverse clinical outcomes and an increased tumor immune response [41]. This may explain the higher immune scores and elevated expression of immune checkpoint genes observed in the high-risk group. Furthermore, Professor Wang's team discovered that knocking down RAC3 can inhibit the proliferation and invasion of bladder cancer cells without inducing apoptosis. The underlying mechanism involves RAC3's regulation of the PI3K/AKT/mTOR pathway to modulate autophagy, influencing the biological activity of bladder cancer cells [42, 43]. Similar findings were observed in our study, as indicated by KEGG analysis showing significant differences in the enrichment of the PI3K/AKT pathway between high and low-risk groups. This suggests that RAC3 may impact the progression of bladder cancer by modulating autophagy through the PI3K/AKT pathway.

In this study, we delved further into the role of S100A7 in bladder cancer cells. Our pan-cancer analysis revealed elevated expression of S100A7 in various cancer cells, including bladder cancer. TISIDB analysis indicated a correlation between high S100A7 expression and adverse outcomes in bladder cancer patients, suggesting a close association with tumor progression. Under pathological conditions, increased intracellular expression of S100A7 was linked to enhanced tumor cell proliferation and migration [44]. Once secreted, S100A7 might act as a mediator in the interaction between tumor cells and the tumor microenvironment. Secreted S100A7 can bind to receptors such as the receptor for advanced glycation end products (RAGE) and Toll-like receptor 4 (TLR4), exerting paracrine effects to promote immune cell recruitment and endothelial cell proliferation [45, 46]. Moreover, elevated expression of S100A7 was observed in bladder cancer patients with muscle-invasive tumors, thus piquing our interest. Therefore, we employed two siRNAs to downregulate the expression of S100A7 in T24 and UMUC-3 cells. The results indicated that reducing the expression of S100A7 led to a significant inhibition of bladder cancer cell proliferation and invasion. After the suppression of S100A7 expression, its binding with JAB1 and activation of the RAGE/S100A7 axis were reduced. Consequently, the activation of cancer-related signaling pathways such as AKT, ERK, AP-1, STAT3, and NF-kB was inhibited, leading to a blockade in tumor proliferation and invasion capabilities [44, 45].

With the advancement of machine learning, radiogenomics is gaining increasing recognition. In the context of renal cancer, the use of non-invasive diagnostic methods such as CT or MRI [47], combined with genetic analysis of disease-relevant factors, provides more precise guidance for clinical diagnosis, treatment selection, and prognosis assessment [48]. The application of artificial intelligence has propelled disease diagnosis to new heights, enabling the accurate identification of subtle differences in images such as CT and MRI and precise identification of tumor subtypes that are challenging to distinguish [49]. This significantly enhances the effectiveness and accuracy of diagnosis.

The close association between apoptosis dysregulation and the prognosis of bladder cancer suggests that conducting relevant genetic tests aids in understanding patient prognosis and facilitates personalized treatment selection. Of particular note is the close integration of our model with immunotherapy, allowing the selection of different therapeutic agents based on individual patient differences. In future treatments, the combination of artificial intelligence and radiogenomics, establishing a radiogenomics approach that integrates apoptosis dysregulation with imaging, will play a crucial role in the diagnosis, treatment selection, and prognosis evaluation of bladder cancer.

At the same time, it is undeniable that our study has its limitations. Firstly, our cohort studies are based on public databases and do not include our hospital's cohort. Secondly, the regulatory mechanism of S100A7 on the proliferation and migration capabilities of bladder cancer cells was not further investigated.

Overall, our study reveals the significant role of anoikis in bladder cancer. Moreover, we have successfully established a stable and accurate prognostic model that can predict patient outcomes and provide valuable insights for the effective use of immunotherapy and drugs. Additionally, our data analysis and in vitro experiments have identified a promising therapeutic target for bladder cancer treatment, namely S100A7. These findings are meaningful and have the potential to advance the field of BLCA research, possibly improving treatment outcomes for patients.

### Supplementary Information

**Additional file 1.** The different drug sensitivity between high and low-risk groups in TCGA cohort.

## Data Availability

The datasets generated and/or analyzed in this study are available at the Cancer Genome Atlas (TCGA) database (https://portal.gdc.cancer.gov/) and the Gene Expression Omnibus (GEO) database (https://www.ncbi.nlm.nih.gov/geo/). For access to additional data results, interested parties may communicate with the corresponding author, subject to obtaining consent.
