# Supplementary figures and images for: Deciphering the significance of anoikis in bladder cancer and systematic analysis of S100A7 as a potential therapeutic target
Source: Eur J Med Res. 2024 Jan 12;29:52. doi: 10.1186/s40001-024-01642-9 (PMC10785515; doi:10.1186/s40001-024-01642-9)

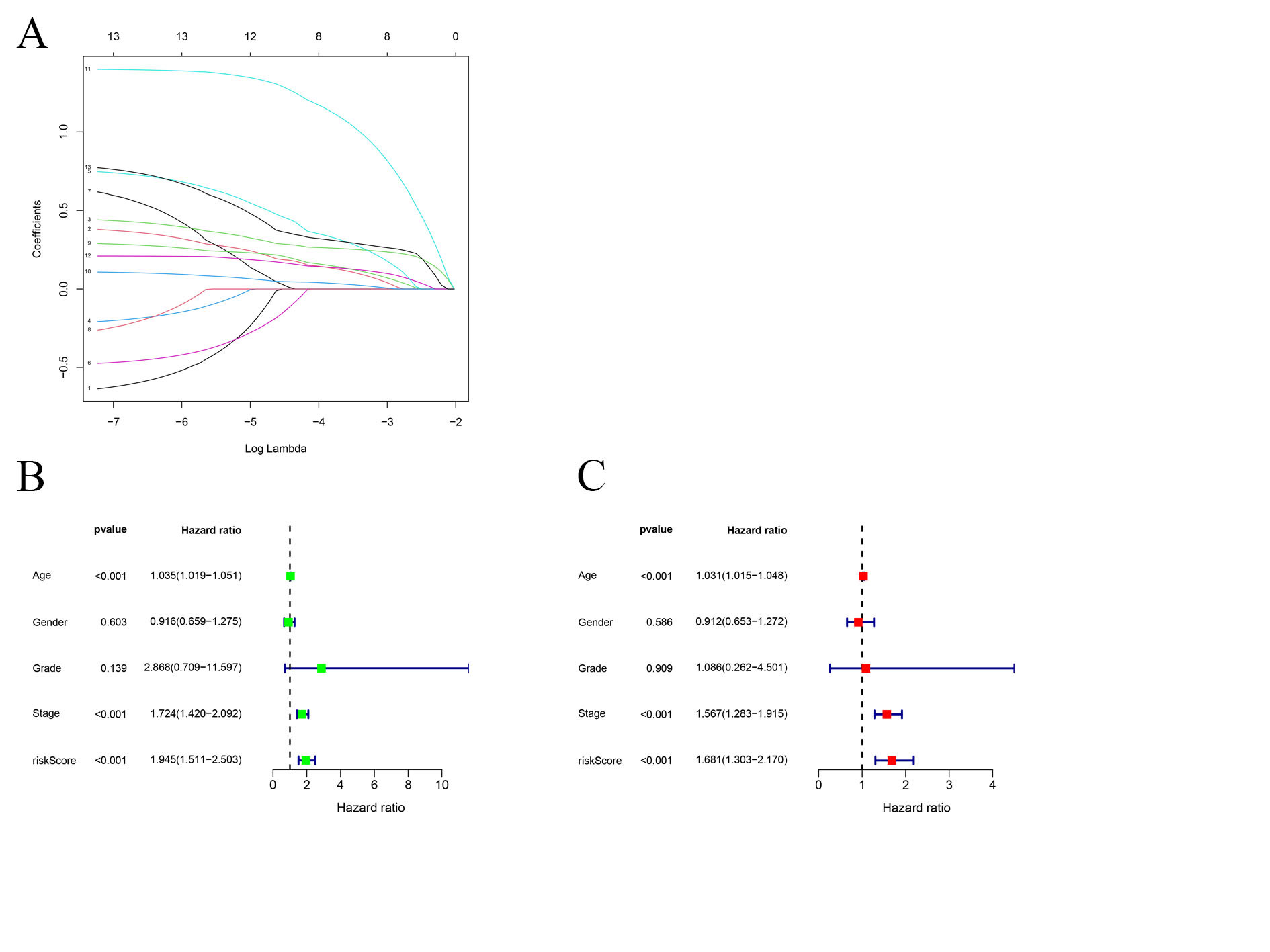

Supplement: Supplementary file 1 — Additional file 1. The different drug sensitivity between high and low-risk groups in TCGA cohort. [file 40001_2024_1642_MOESM1_ESM.zip › Supplementary material/Supplementary Figure1.jpg]

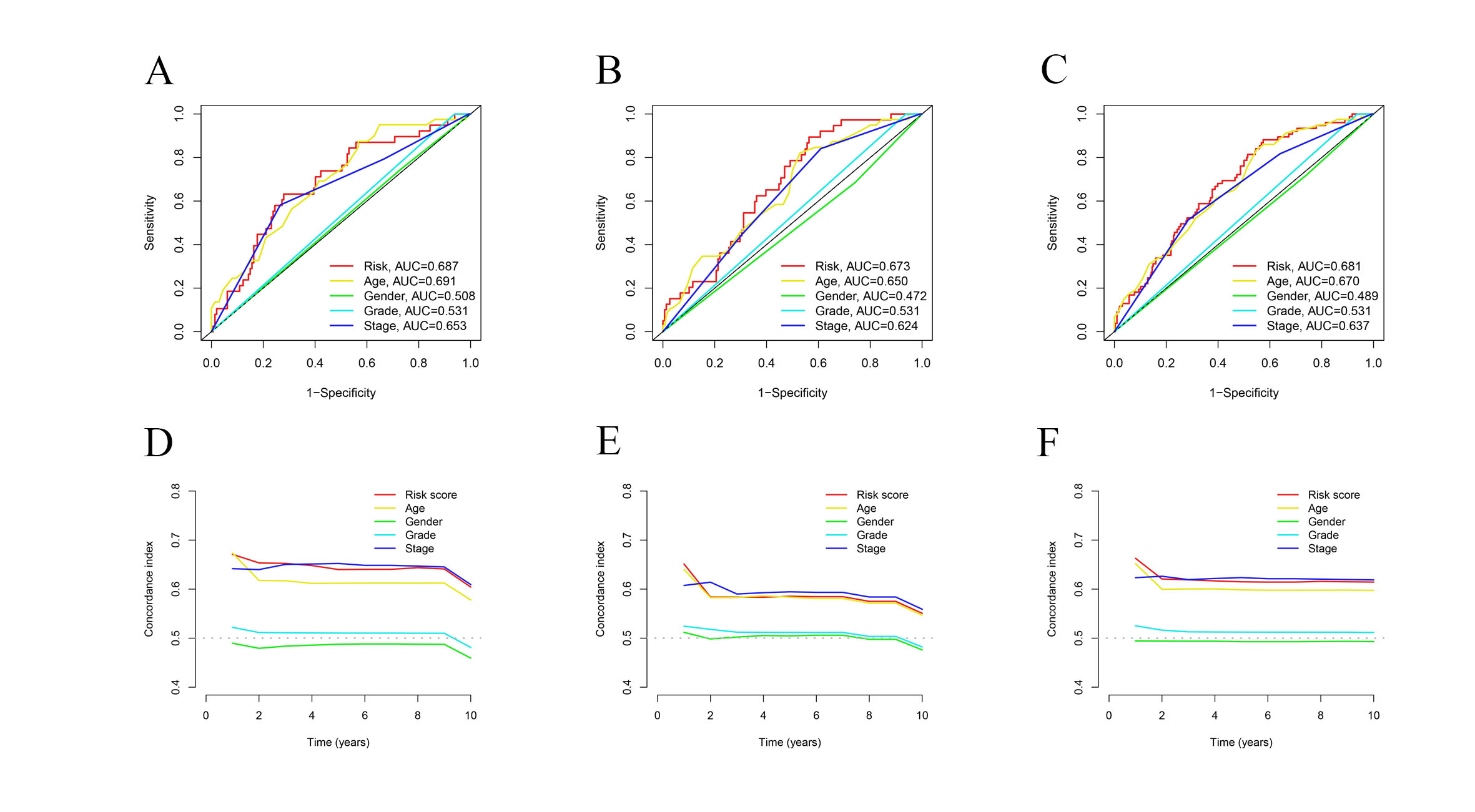

Supplement: Supplementary file 1 — Additional file 1. The different drug sensitivity between high and low-risk groups in TCGA cohort. [file 40001_2024_1642_MOESM1_ESM.zip › Supplementary material/Supplementary Figure2.jpg]

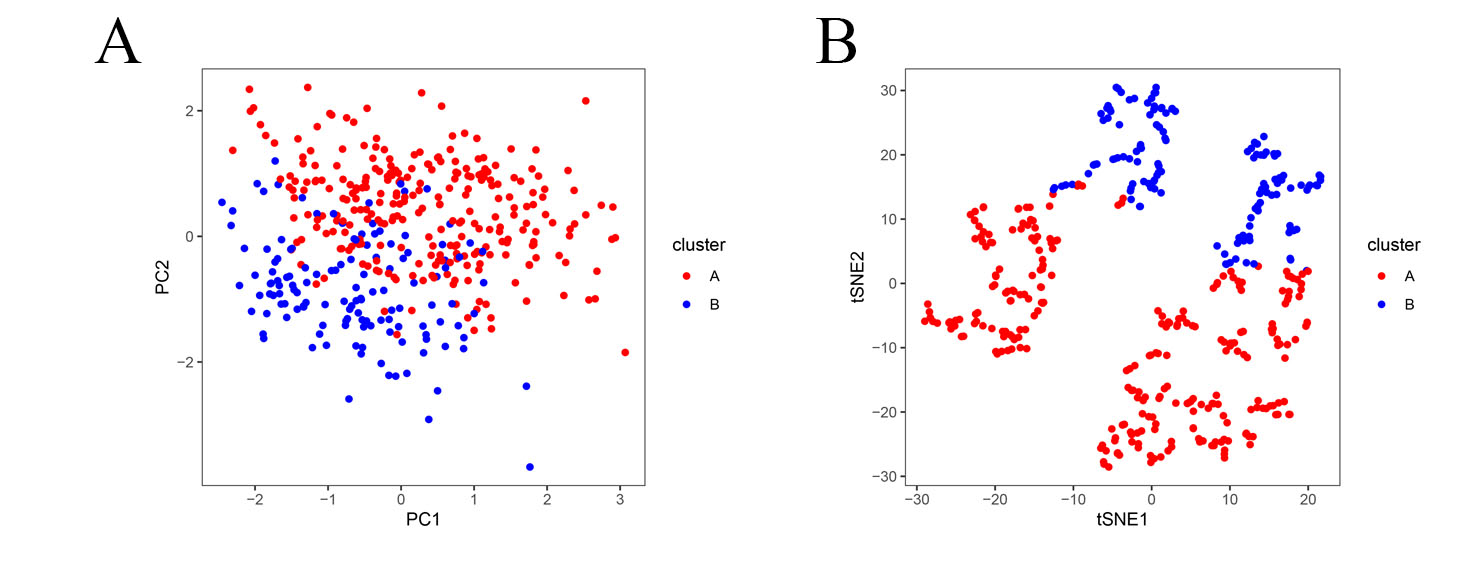

Supplement: Supplementary file 1 — Additional file 1. The different drug sensitivity between high and low-risk groups in TCGA cohort. [file 40001_2024_1642_MOESM1_ESM.zip › Supplementary material/Supplementary Figure3.jpg]

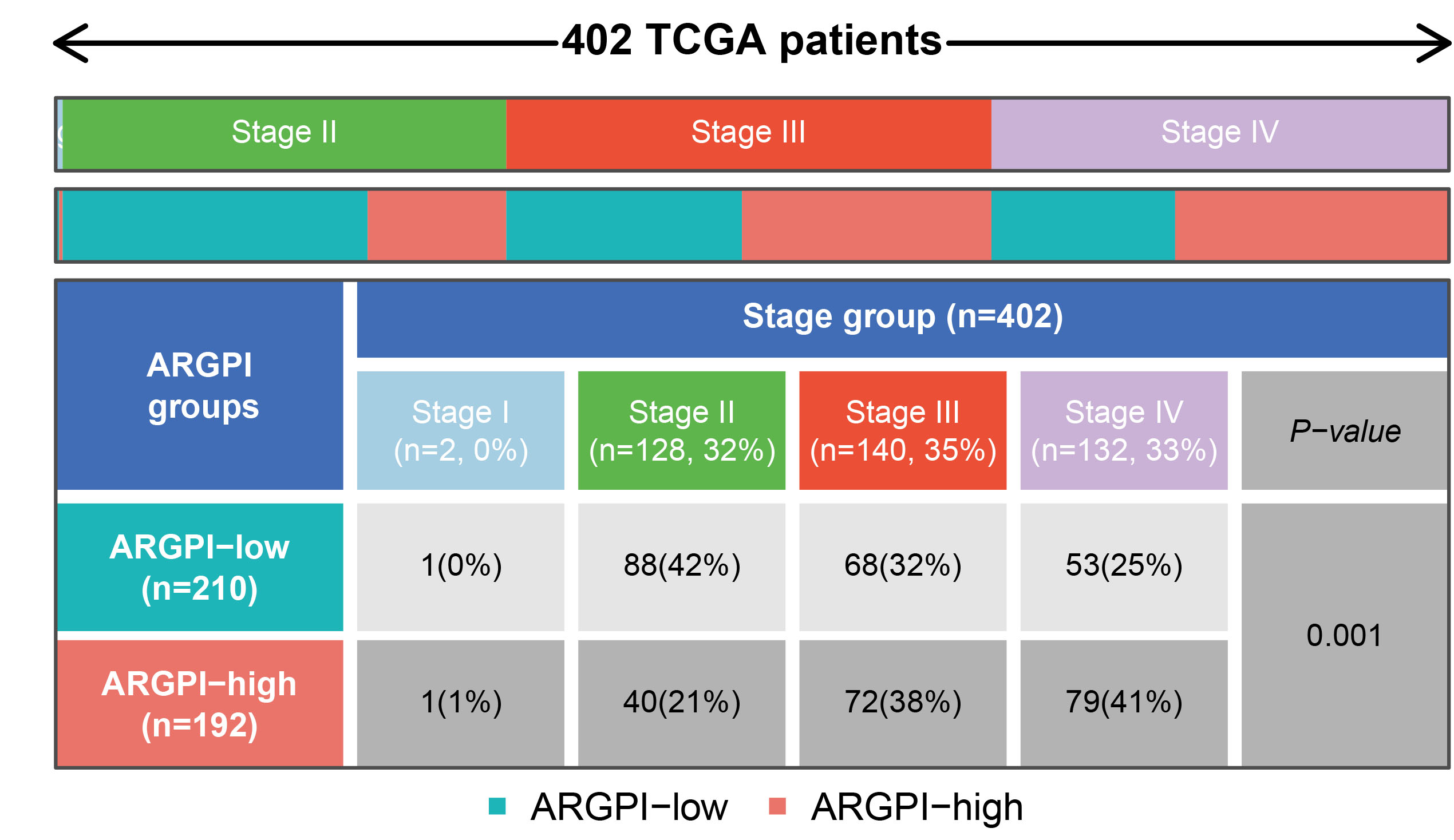

Supplement: Supplementary file 1 — Additional file 1. The different drug sensitivity between high and low-risk groups in TCGA cohort. [file 40001_2024_1642_MOESM1_ESM.zip › Supplementary material/Supplementary Figure4.jpg]

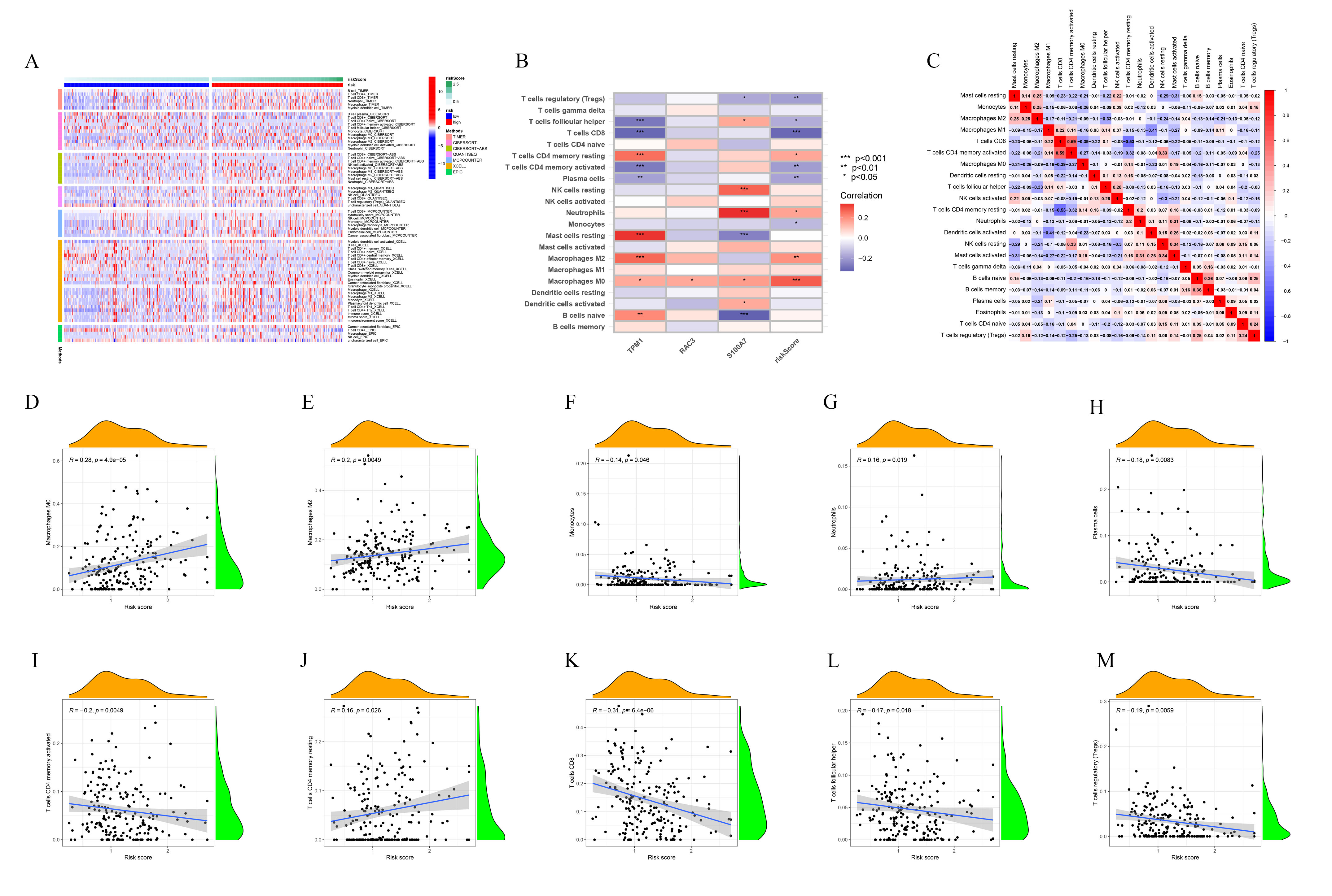

Supplement: Supplementary file 1 — Additional file 1. The different drug sensitivity between high and low-risk groups in TCGA cohort. [file 40001_2024_1642_MOESM1_ESM.zip › Supplementary material/Supplementary Figure5.jpg]

Risk low high

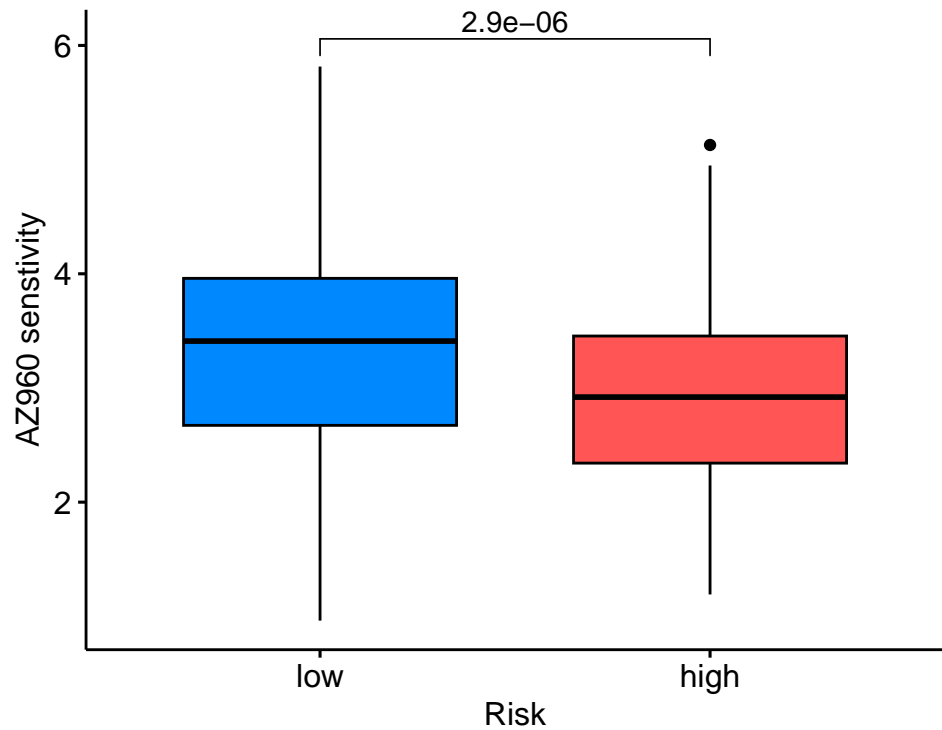

Supplement: Supplementary file 1 — Additional file 1. The different drug sensitivity between high and low-risk groups in TCGA cohort. [file 40001_2024_1642_MOESM1_ESM.zip › Supplementary material/supplementary file 1/drugSenstivity.AZ960.pdf]

Risk 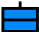 low 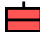 high

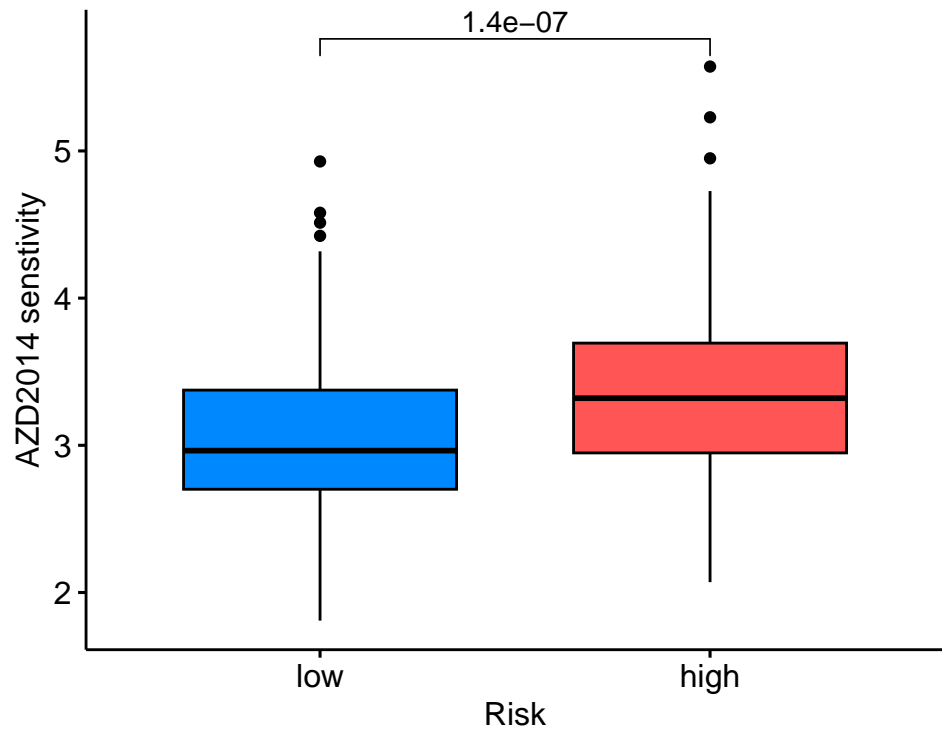

Supplement: Supplementary file 1 — Additional file 1. The different drug sensitivity between high and low-risk groups in TCGA cohort. [file 40001_2024_1642_MOESM1_ESM.zip › Supplementary material/supplementary file 1/drugSenstivity.AZD2014.pdf]

Risk low high

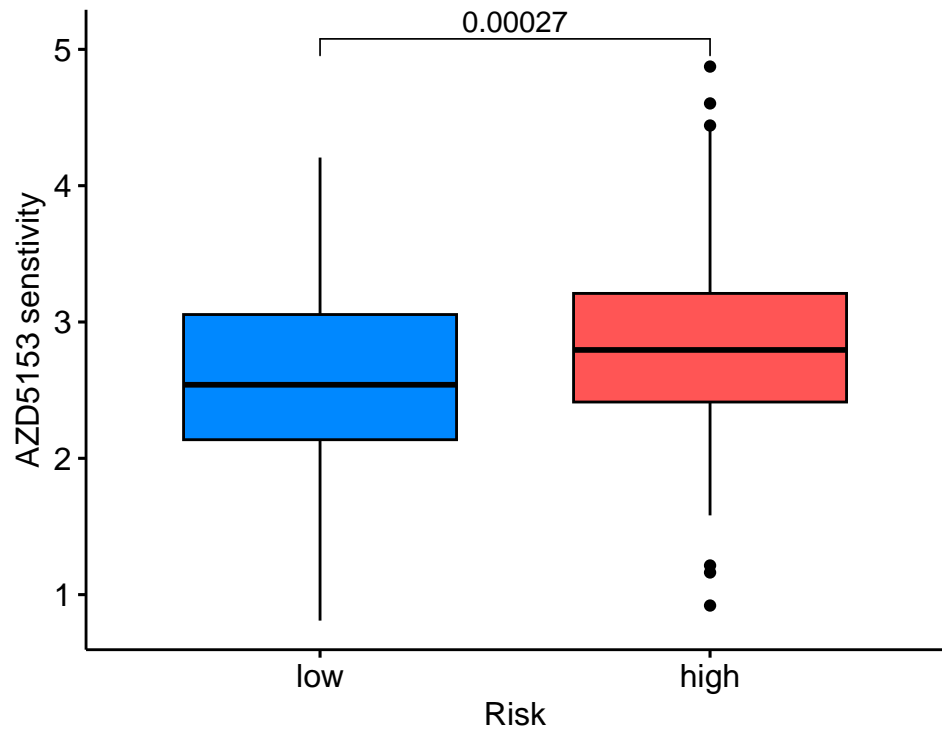

Supplement: Supplementary file 1 — Additional file 1. The different drug sensitivity between high and low-risk groups in TCGA cohort. [file 40001_2024_1642_MOESM1_ESM.zip › Supplementary material/supplementary file 1/drugSenstivity.AZD5153.pdf]

Risk low high

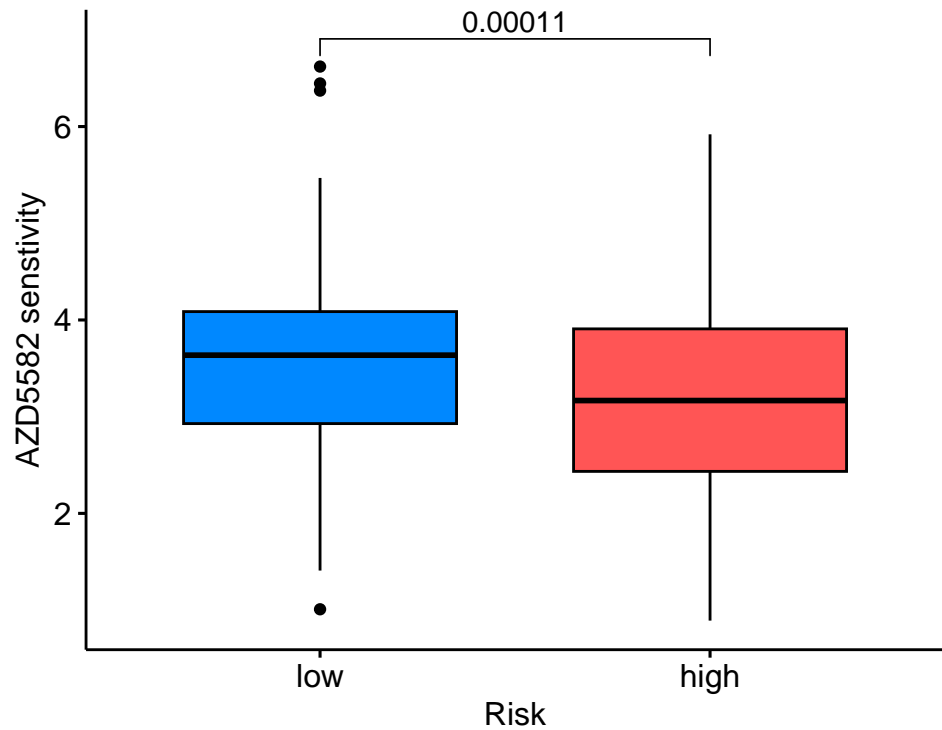

Supplement: Supplementary file 1 — Additional file 1. The different drug sensitivity between high and low-risk groups in TCGA cohort. [file 40001_2024_1642_MOESM1_ESM.zip › Supplementary material/supplementary file 1/drugSenstivity.AZD5582.pdf]

Risk 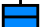 low 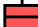 high

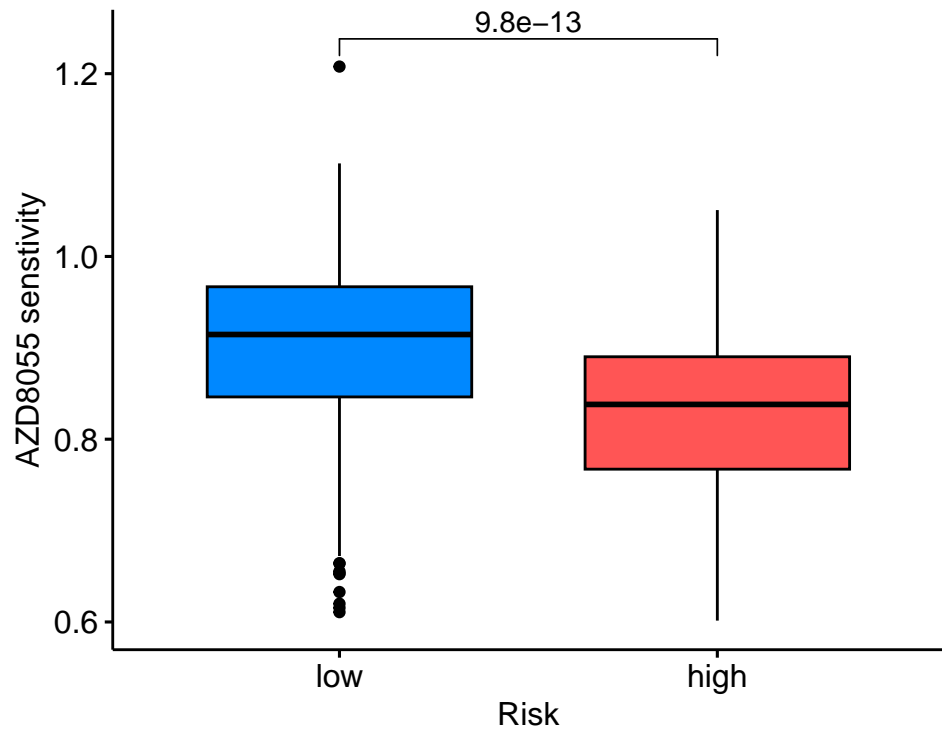

Supplement: Supplementary file 1 — Additional file 1. The different drug sensitivity between high and low-risk groups in TCGA cohort. [file 40001_2024_1642_MOESM1_ESM.zip › Supplementary material/supplementary file 1/drugSenstivity.AZD8055.pdf]

Risk low high

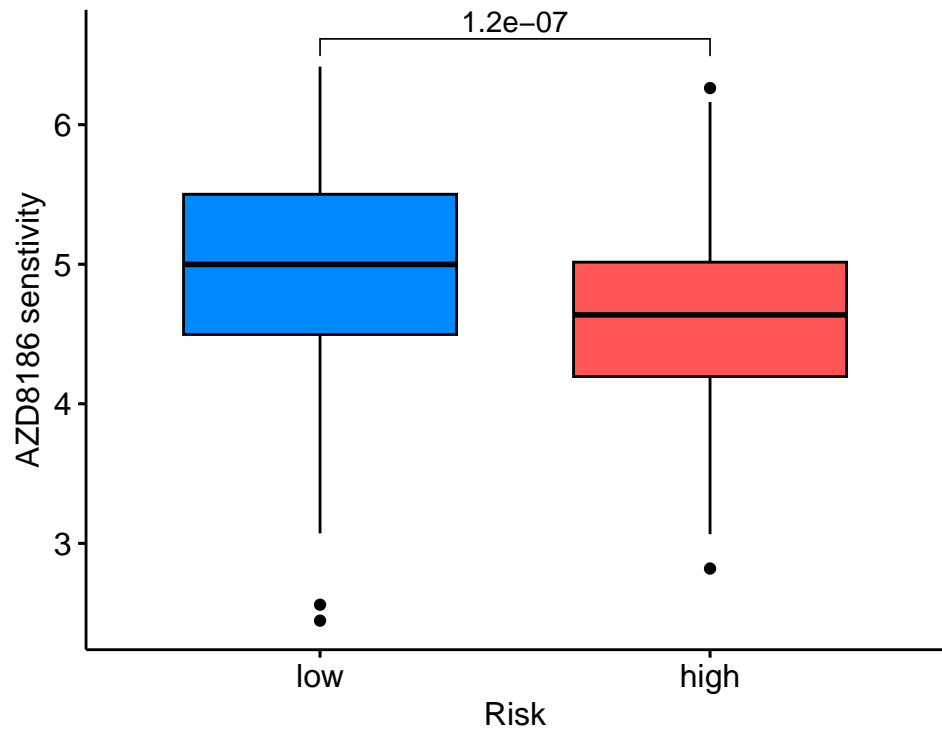

Supplement: Supplementary file 1 — Additional file 1. The different drug sensitivity between high and low-risk groups in TCGA cohort. [file 40001_2024_1642_MOESM1_ESM.zip › Supplementary material/supplementary file 1/drugSenstivity.AZD8186.pdf]

Risk 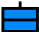 low 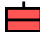 high

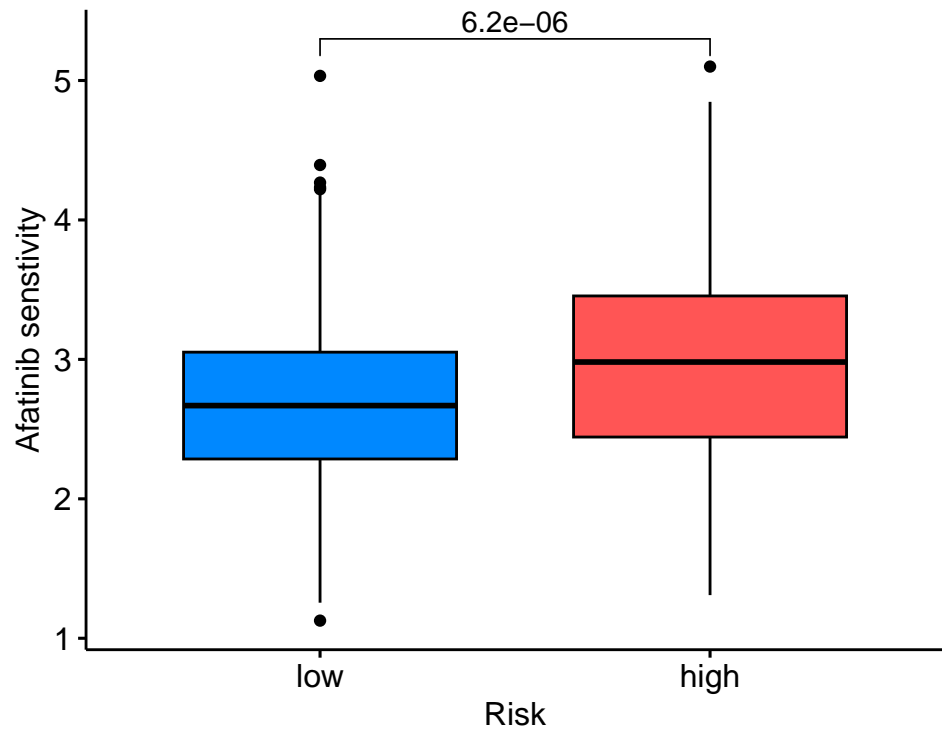

Supplement: Supplementary file 1 — Additional file 1. The different drug sensitivity between high and low-risk groups in TCGA cohort. [file 40001_2024_1642_MOESM1_ESM.zip › Supplementary material/supplementary file 1/drugSenstivity.Afatinib.pdf]

Risk 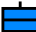 low 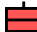 high

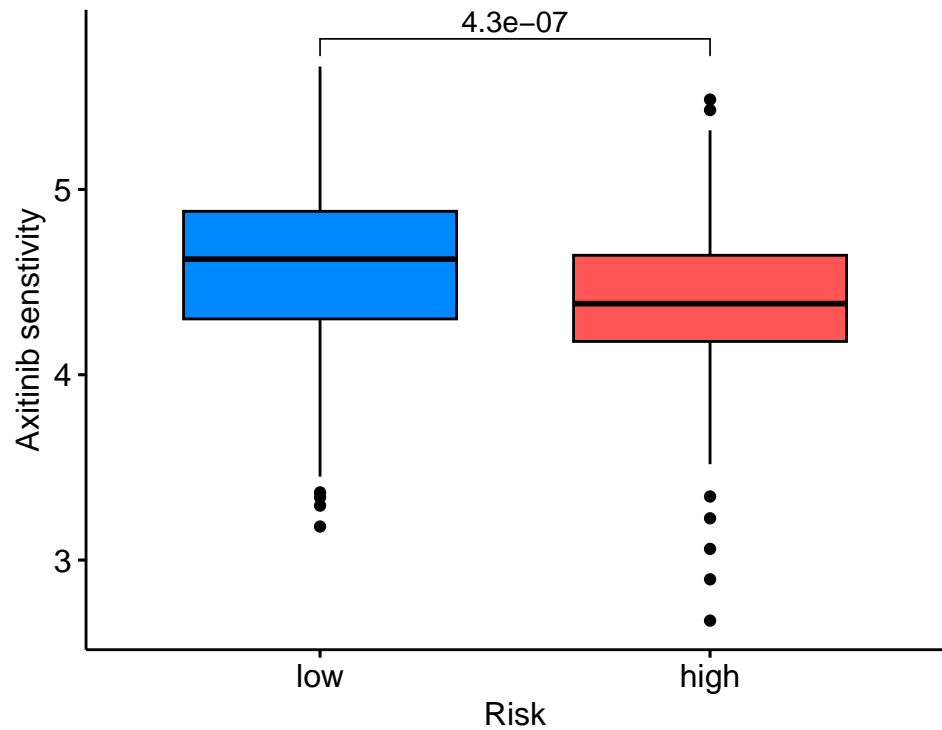

Supplement: Supplementary file 1 — Additional file 1. The different drug sensitivity between high and low-risk groups in TCGA cohort. [file 40001_2024_1642_MOESM1_ESM.zip › Supplementary material/supplementary file 1/drugSenstivity.Axitinib.pdf]

Risk 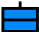 low 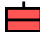 high

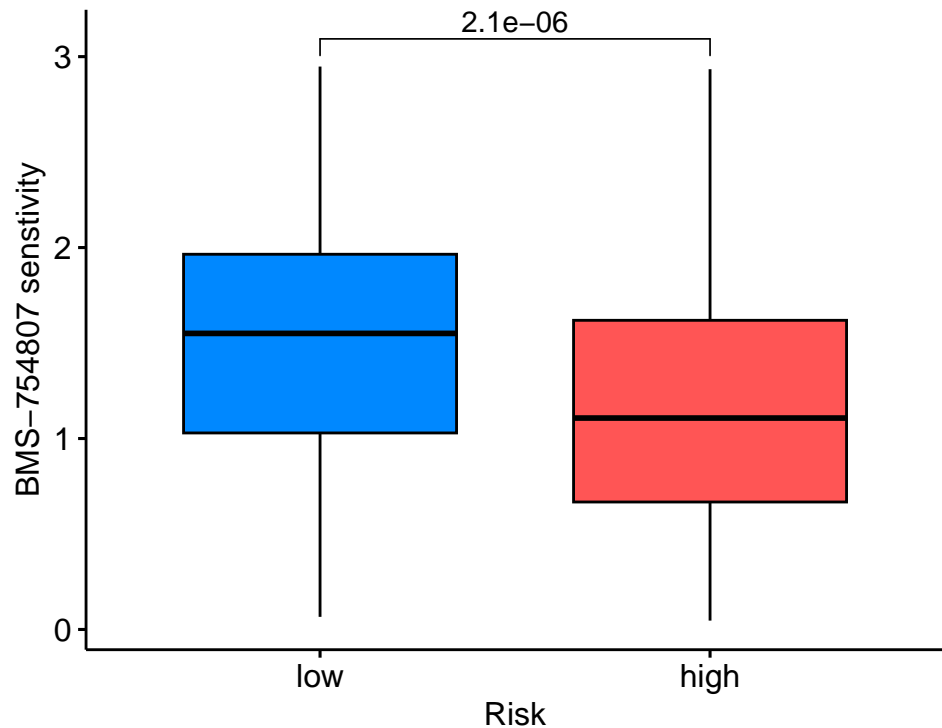

Supplement: Supplementary file 1 — Additional file 1. The different drug sensitivity between high and low-risk groups in TCGA cohort. [file 40001_2024_1642_MOESM1_ESM.zip › Supplementary material/supplementary file 1/drugSenstivity.BMS-754807.pdf]

Risk 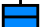 low 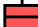 high

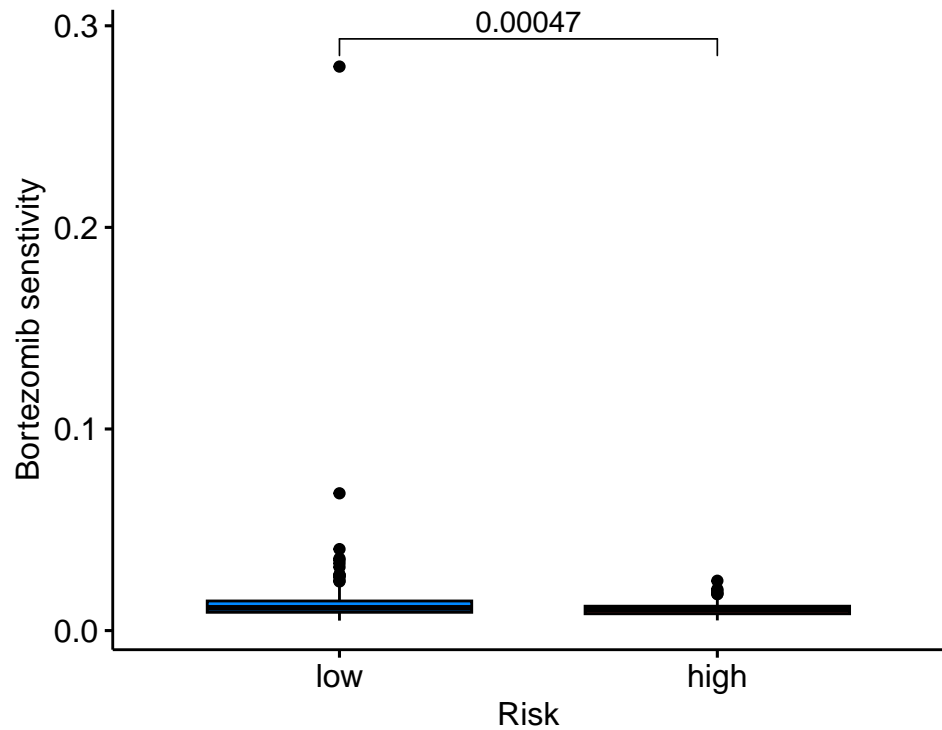

Supplement: Supplementary file 1 — Additional file 1. The different drug sensitivity between high and low-risk groups in TCGA cohort. [file 40001_2024_1642_MOESM1_ESM.zip › Supplementary material/supplementary file 1/drugSenstivity.Bortezomib.pdf]

Risk 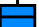 low 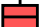 high

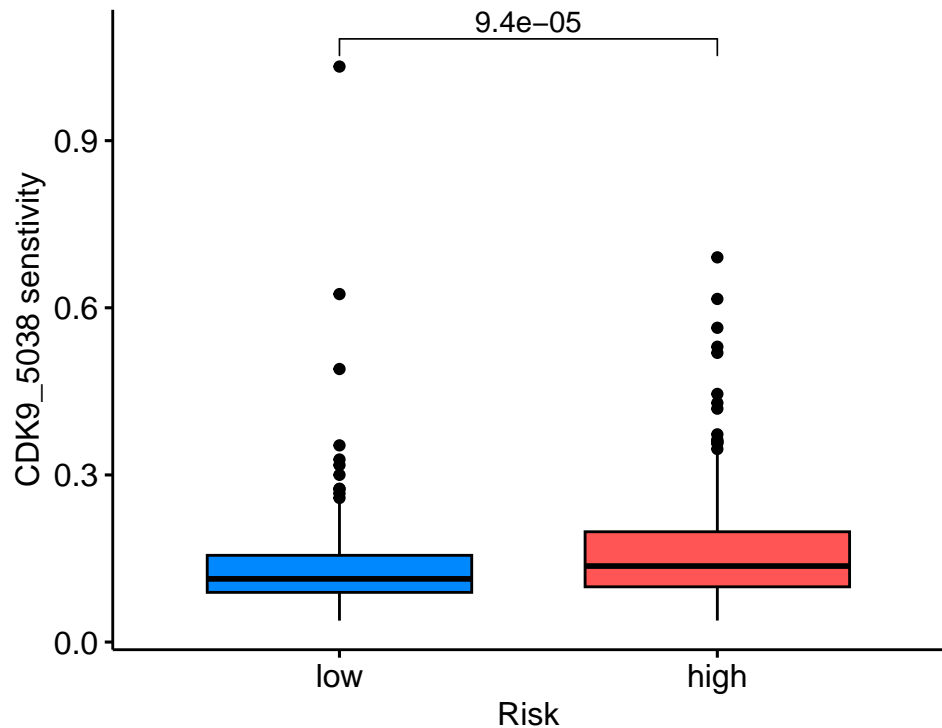

Supplement: Supplementary file 1 — Additional file 1. The different drug sensitivity between high and low-risk groups in TCGA cohort. [file 40001_2024_1642_MOESM1_ESM.zip › Supplementary material/supplementary file 1/drugSenstivity.CDK9_5038.pdf]

Risk 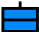 low 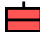 high

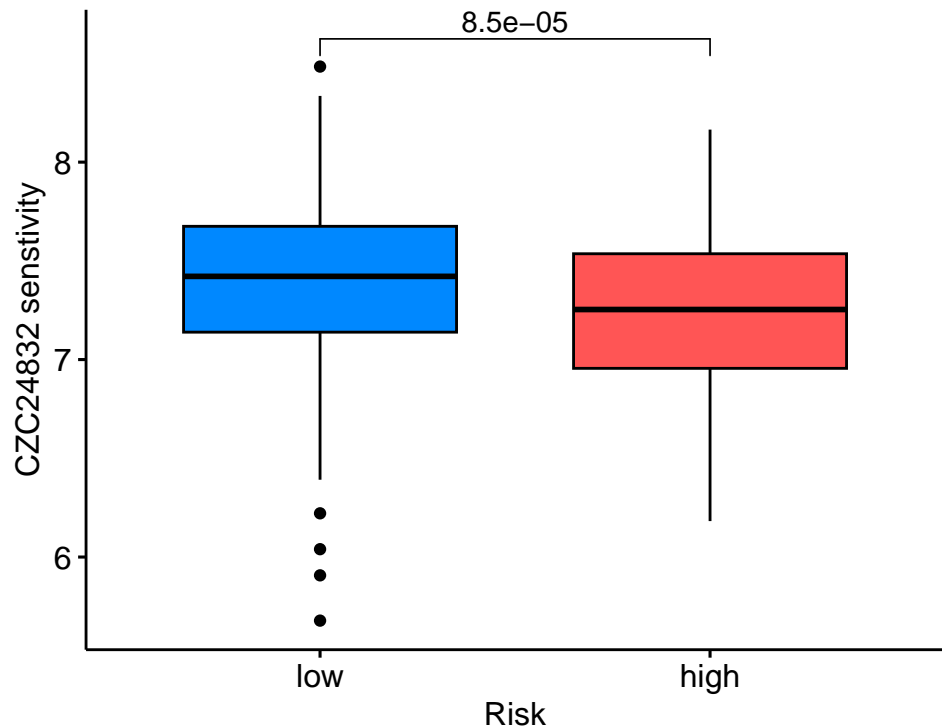

Supplement: Supplementary file 1 — Additional file 1. The different drug sensitivity between high and low-risk groups in TCGA cohort. [file 40001_2024_1642_MOESM1_ESM.zip › Supplementary material/supplementary file 1/drugSenstivity.CZC24832.pdf]

Risk 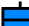 low 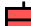 high

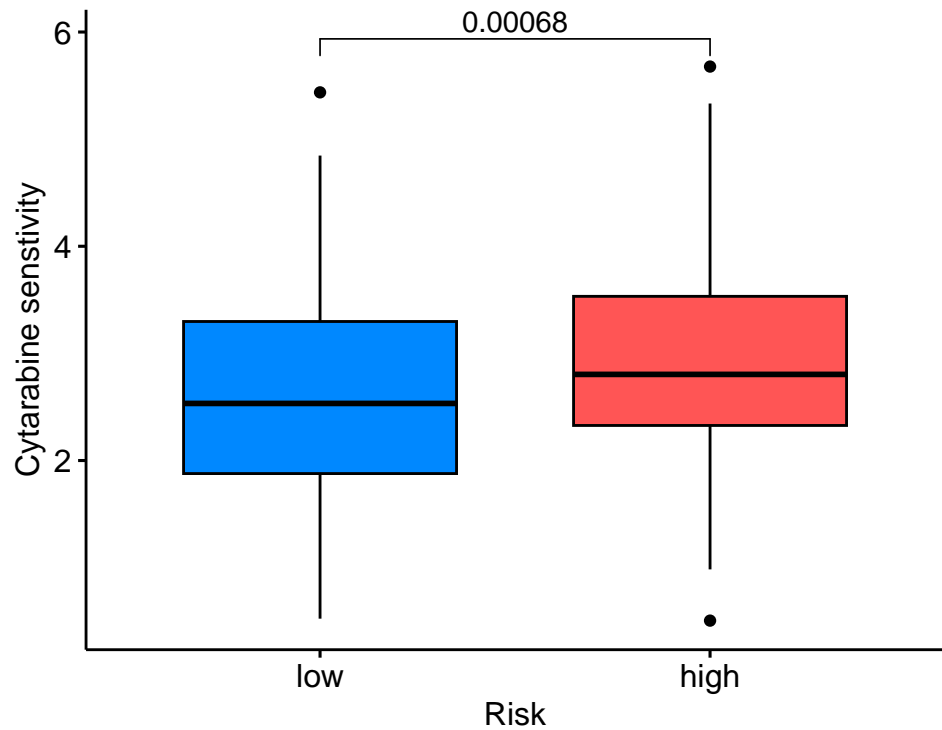

Supplement: Supplementary file 1 — Additional file 1. The different drug sensitivity between high and low-risk groups in TCGA cohort. [file 40001_2024_1642_MOESM1_ESM.zip › Supplementary material/supplementary file 1/drugSenstivity.Cytarabine.pdf]

Risk 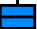 low 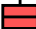 high

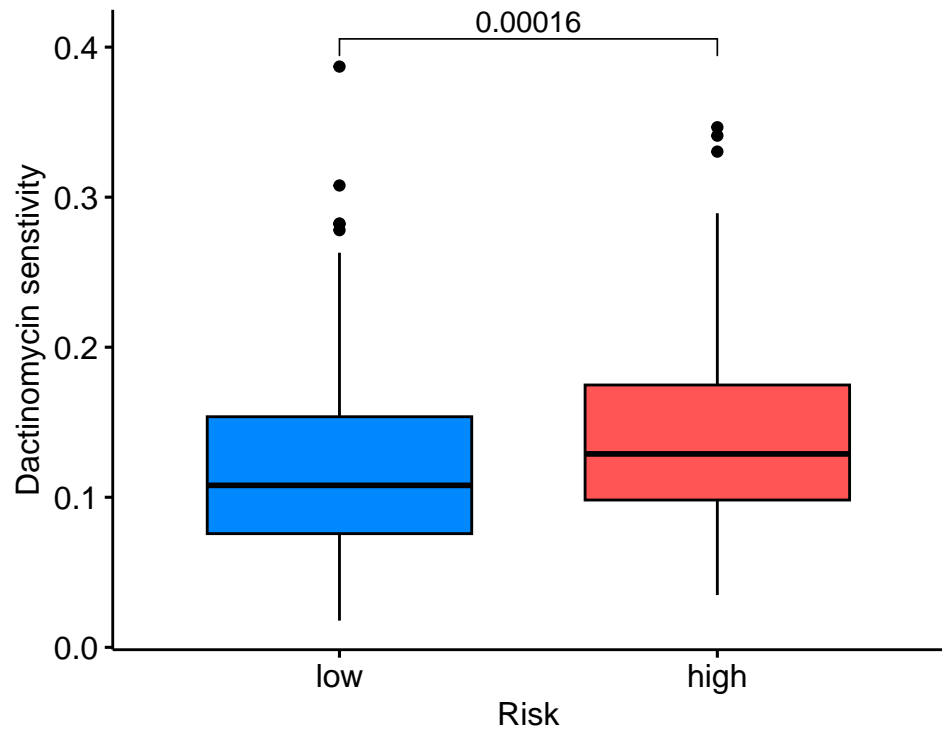

Supplement: Supplementary file 1 — Additional file 1. The different drug sensitivity between high and low-risk groups in TCGA cohort. [file 40001_2024_1642_MOESM1_ESM.zip › Supplementary material/supplementary file 1/drugSenstivity.Dactinomycin.pdf]

Risk 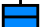 low 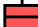 high

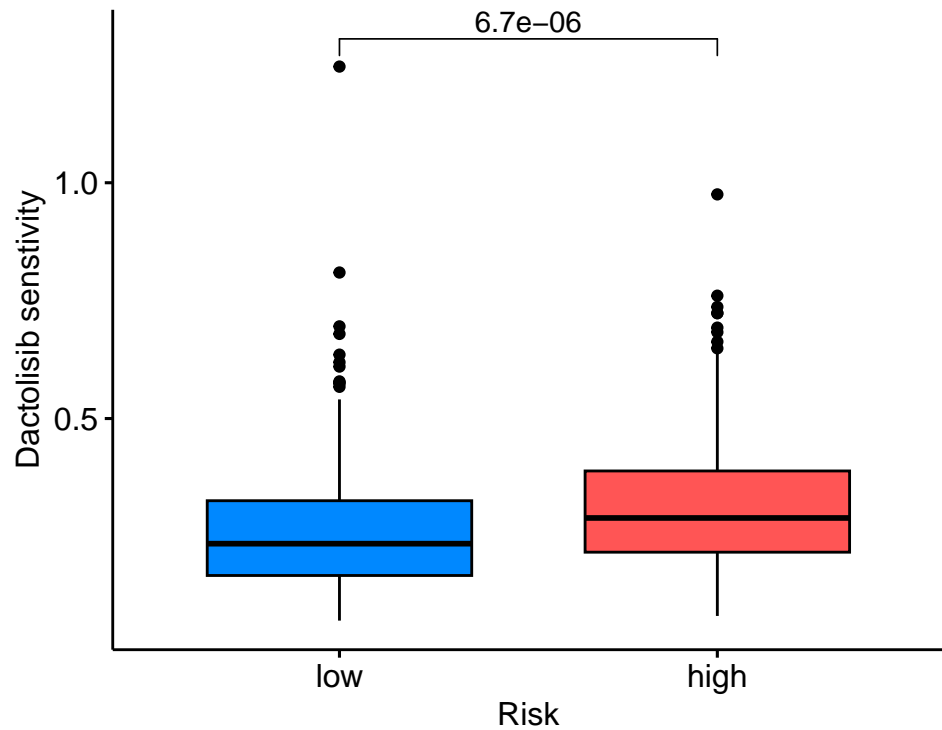

Supplement: Supplementary file 1 — Additional file 1. The different drug sensitivity between high and low-risk groups in TCGA cohort. [file 40001_2024_1642_MOESM1_ESM.zip › Supplementary material/supplementary file 1/drugSenstivity.Dactolisib.pdf]

Risk 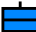 low 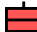 high

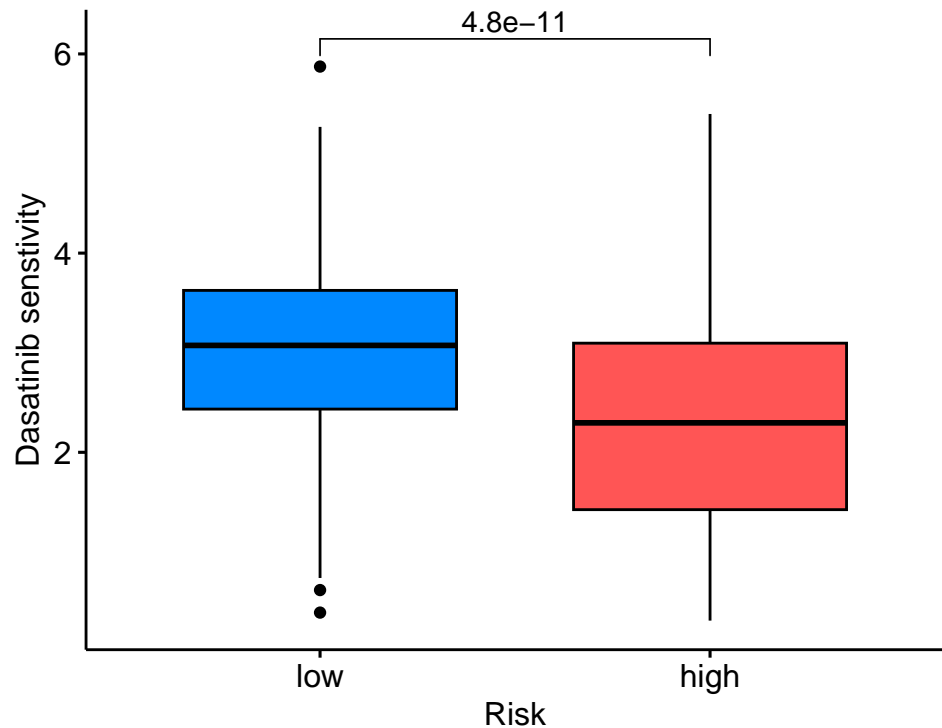

Supplement: Supplementary file 1 — Additional file 1. The different drug sensitivity between high and low-risk groups in TCGA cohort. [file 40001_2024_1642_MOESM1_ESM.zip › Supplementary material/supplementary file 1/drugSenstivity.Dasatinib.pdf]

Risk low high

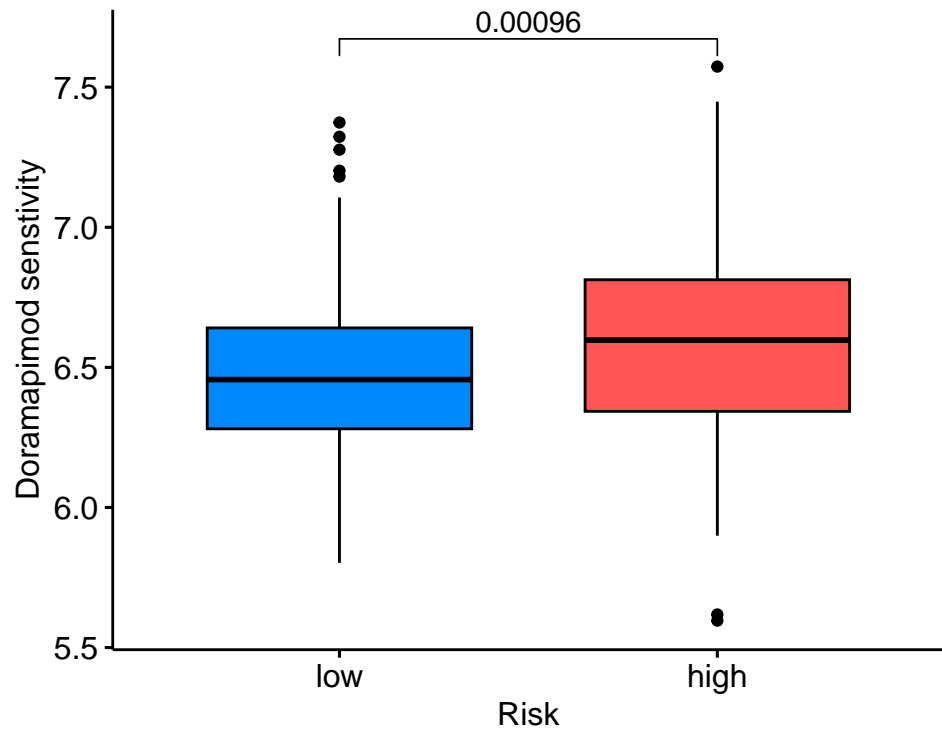

Supplement: Supplementary file 1 — Additional file 1. The different drug sensitivity between high and low-risk groups in TCGA cohort. [file 40001_2024_1642_MOESM1_ESM.zip › Supplementary material/supplementary file 1/drugSenstivity.Doramapimod.pdf]

Risk low high

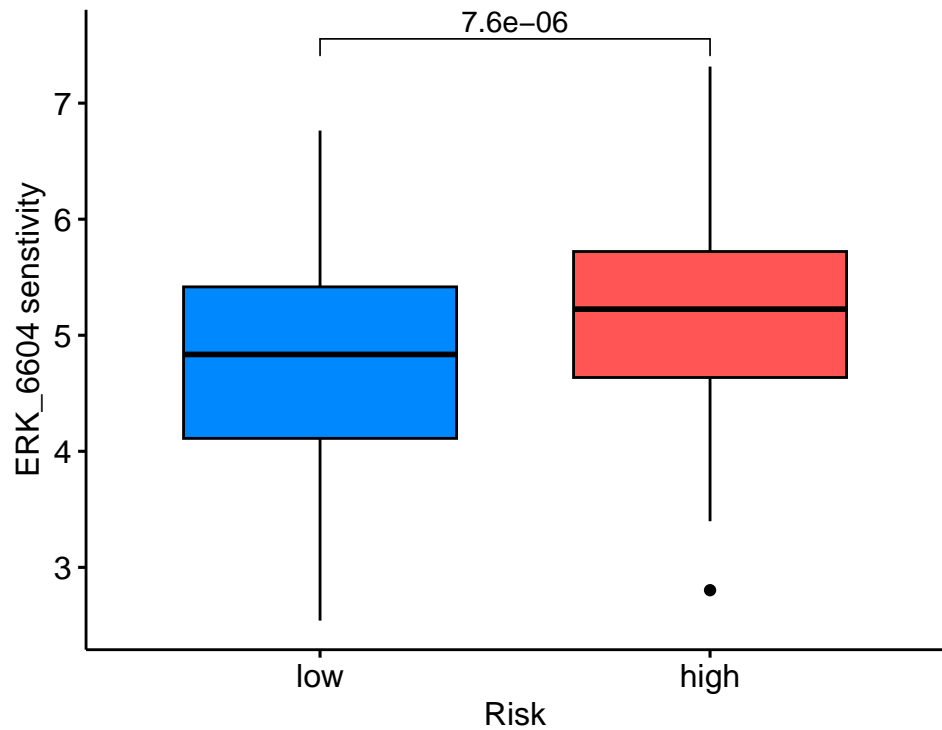

Supplement: Supplementary file 1 — Additional file 1. The different drug sensitivity between high and low-risk groups in TCGA cohort. [file 40001_2024_1642_MOESM1_ESM.zip › Supplementary material/supplementary file 1/drugSenstivity.ERK_6604.pdf]

Risk low high

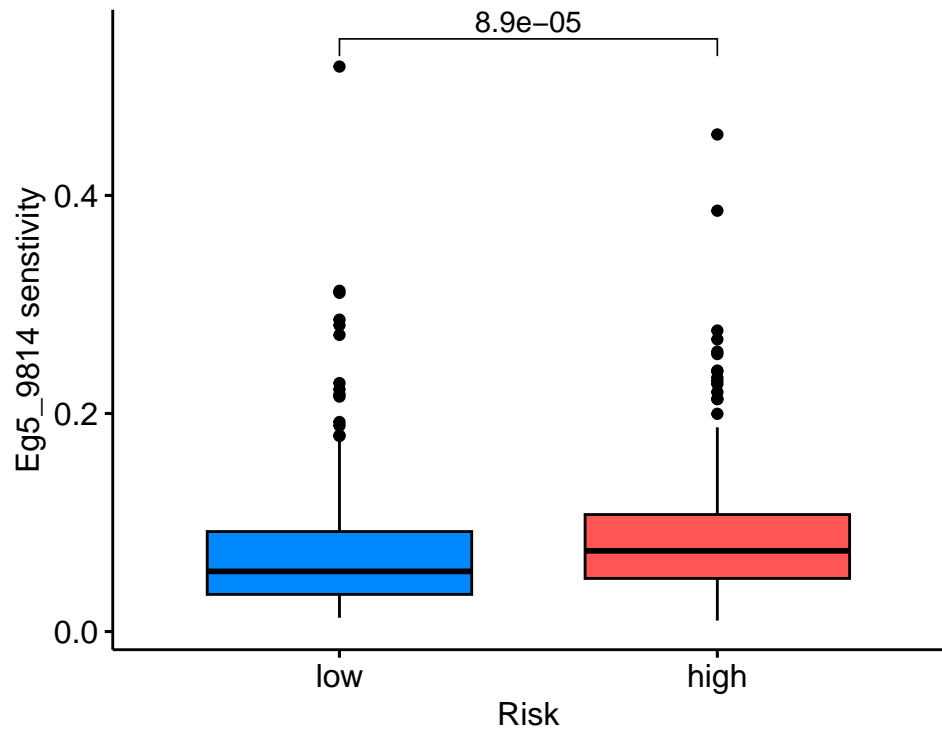

Supplement: Supplementary file 1 — Additional file 1. The different drug sensitivity between high and low-risk groups in TCGA cohort. [file 40001_2024_1642_MOESM1_ESM.zip › Supplementary material/supplementary file 1/drugSenstivity.Eg5_9814.pdf]

Risk 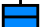 low 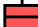 high

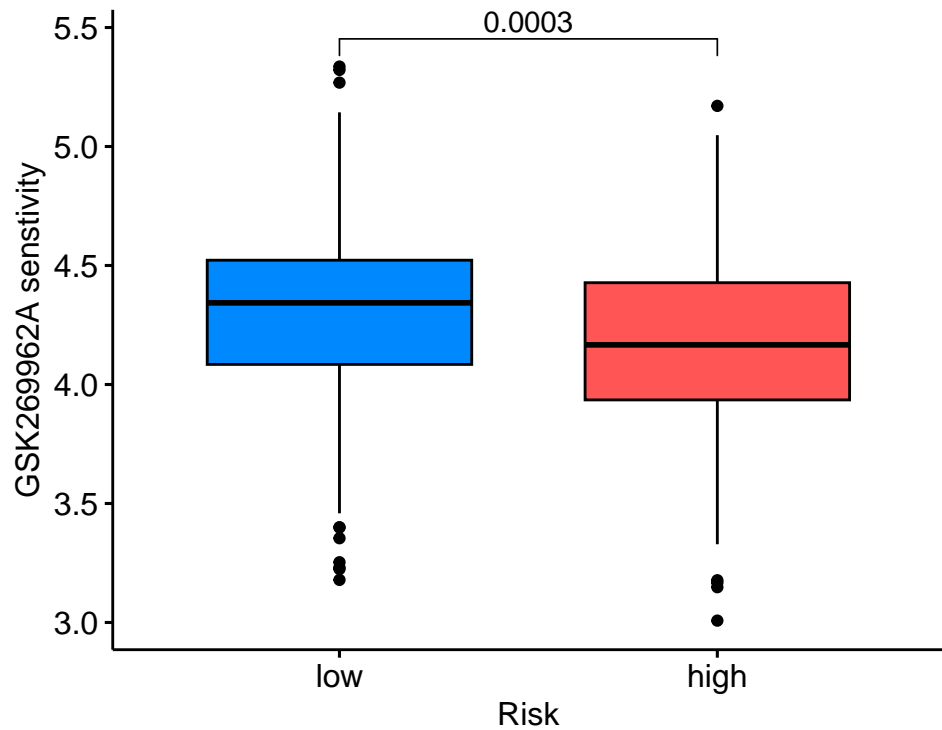

Supplement: Supplementary file 1 — Additional file 1. The different drug sensitivity between high and low-risk groups in TCGA cohort. [file 40001_2024_1642_MOESM1_ESM.zip › Supplementary material/supplementary file 1/drugSenstivity.GSK269962A.pdf]

Risk low high

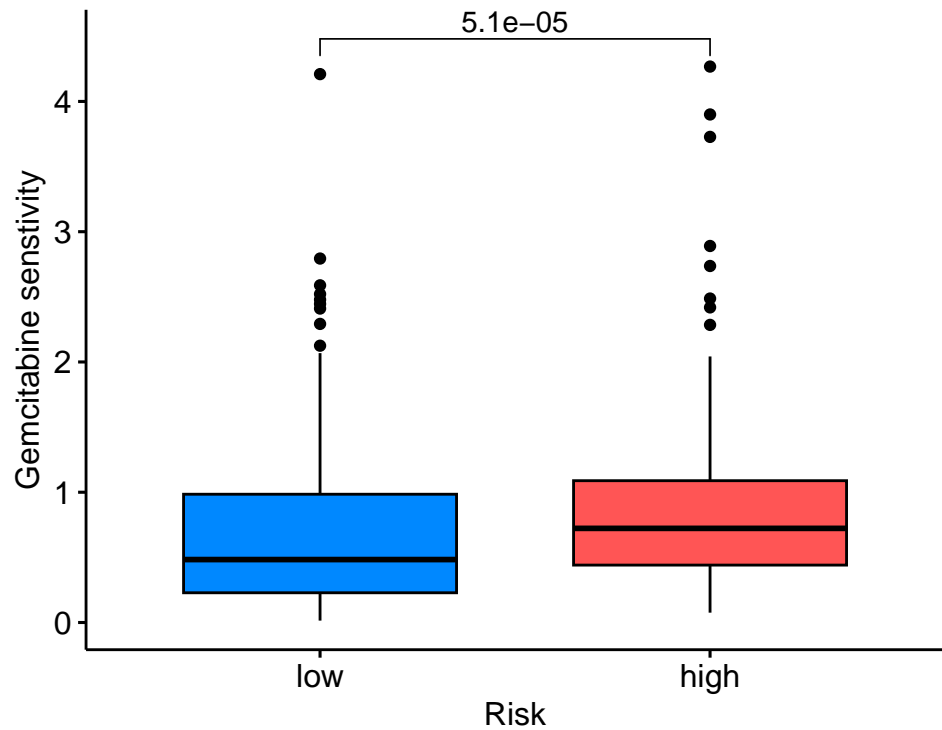

Supplement: Supplementary file 1 — Additional file 1. The different drug sensitivity between high and low-risk groups in TCGA cohort. [file 40001_2024_1642_MOESM1_ESM.zip › Supplementary material/supplementary file 1/drugSenstivity.Gemcitabine.pdf]

Risk low high

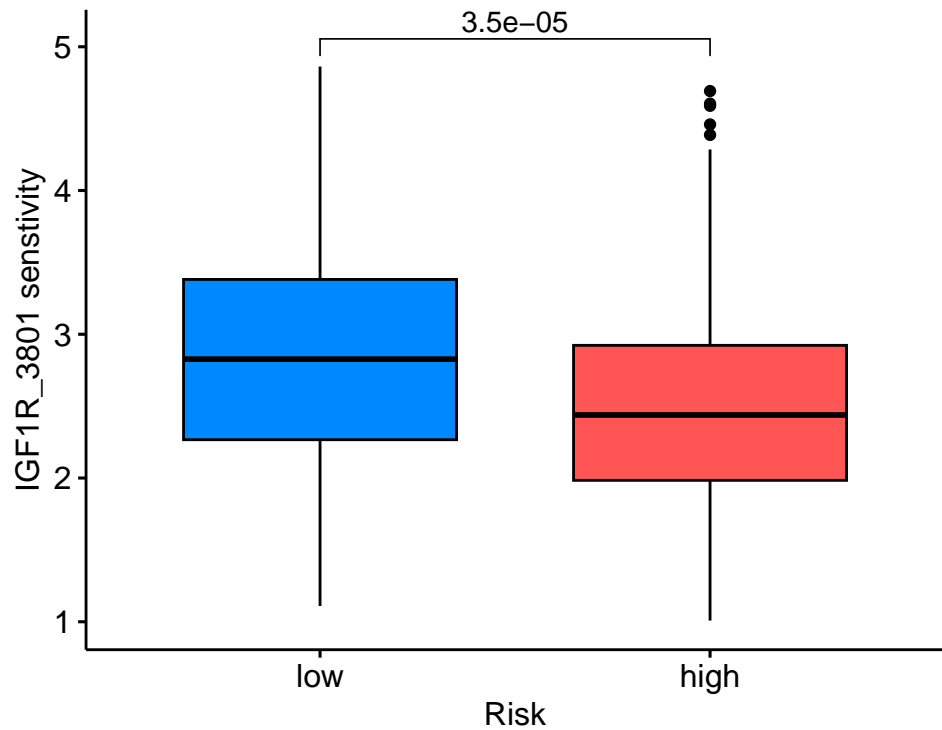

Supplement: Supplementary file 1 — Additional file 1. The different drug sensitivity between high and low-risk groups in TCGA cohort. [file 40001_2024_1642_MOESM1_ESM.zip › Supplementary material/supplementary file 1/drugSenstivity.IGF1R_3801.pdf]

Risk low high

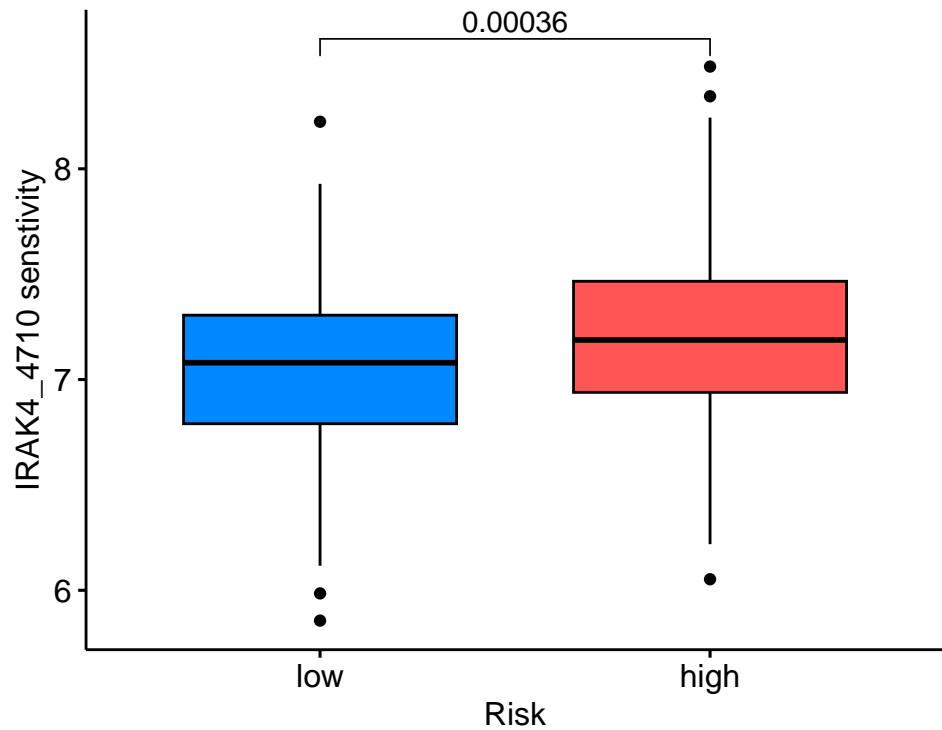

Supplement: Supplementary file 1 — Additional file 1. The different drug sensitivity between high and low-risk groups in TCGA cohort. [file 40001_2024_1642_MOESM1_ESM.zip › Supplementary material/supplementary file 1/drugSenstivity.IRAK4_4710.pdf]

Risk low high

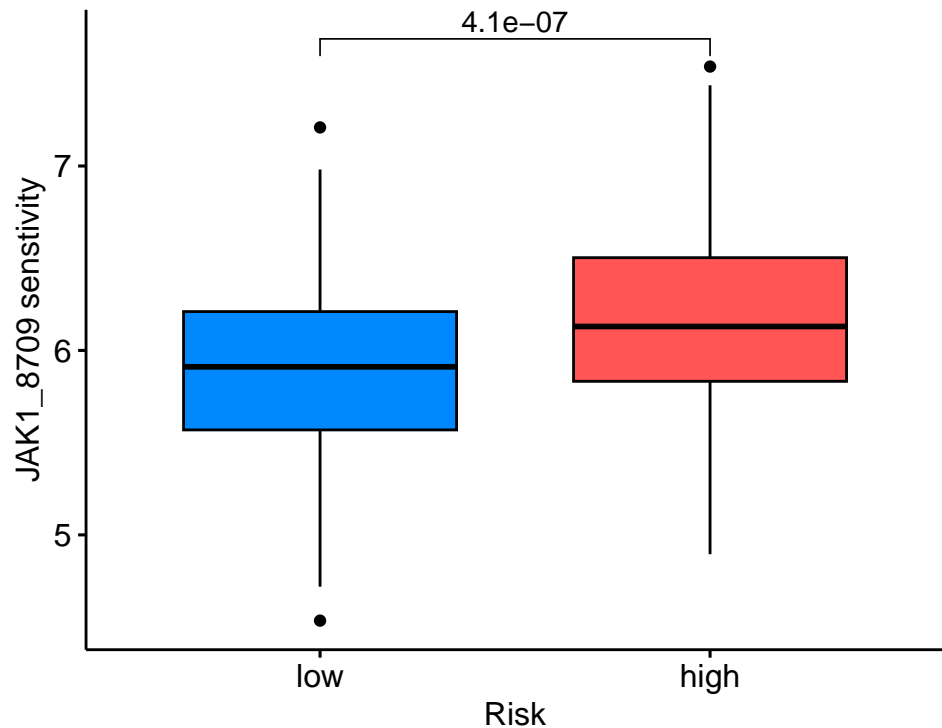

Supplement: Supplementary file 1 — Additional file 1. The different drug sensitivity between high and low-risk groups in TCGA cohort. [file 40001_2024_1642_MOESM1_ESM.zip › Supplementary material/supplementary file 1/drugSenstivity.JAK1_8709.pdf]

Risk 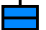 low 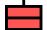 high

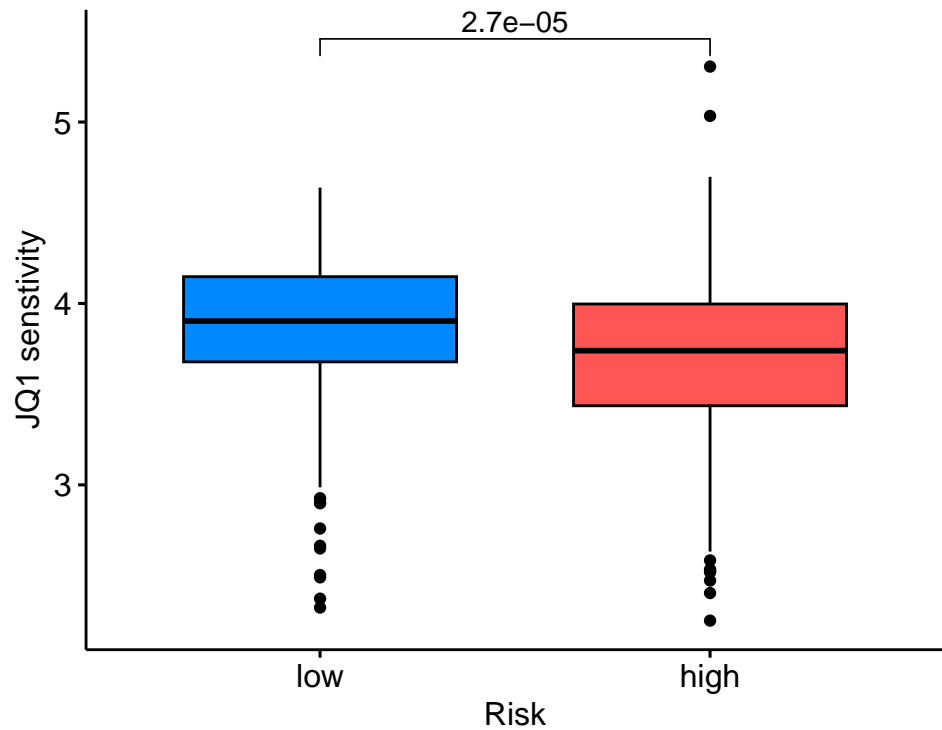

Supplement: Supplementary file 1 — Additional file 1. The different drug sensitivity between high and low-risk groups in TCGA cohort. [file 40001_2024_1642_MOESM1_ESM.zip › Supplementary material/supplementary file 1/drugSenstivity.JQ1.pdf]

Risk 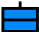 low 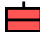 high

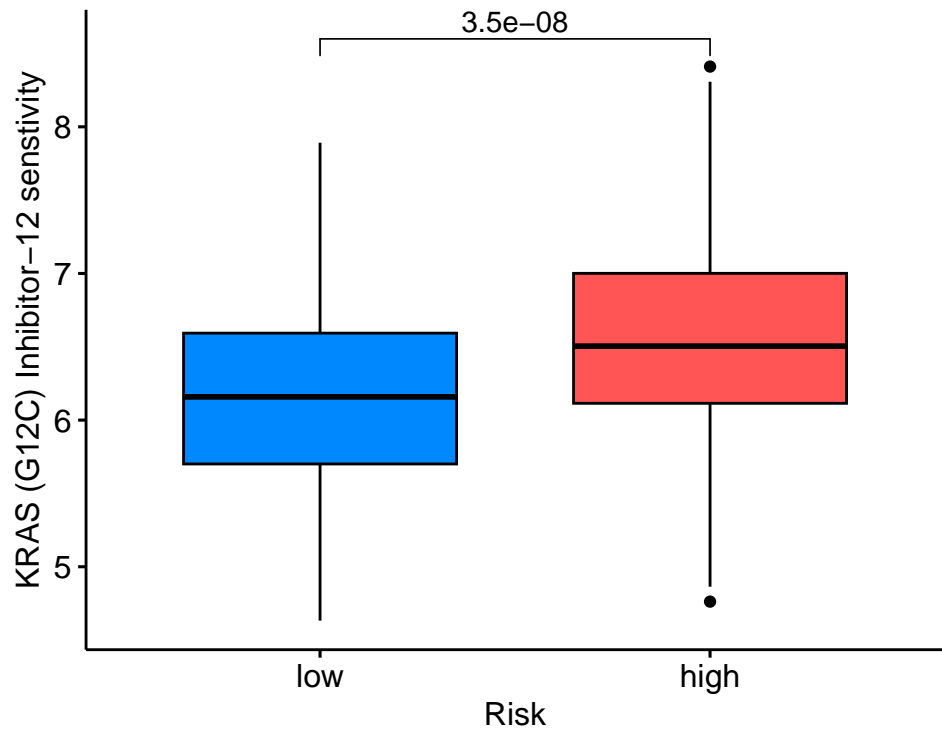

Supplement: Supplementary file 1 — Additional file 1. The different drug sensitivity between high and low-risk groups in TCGA cohort. [file 40001_2024_1642_MOESM1_ESM.zip › Supplementary material/supplementary file 1/drugSenstivity.KRAS (G12C) Inhibitor-12.pdf]

Risk 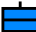 low 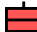 high

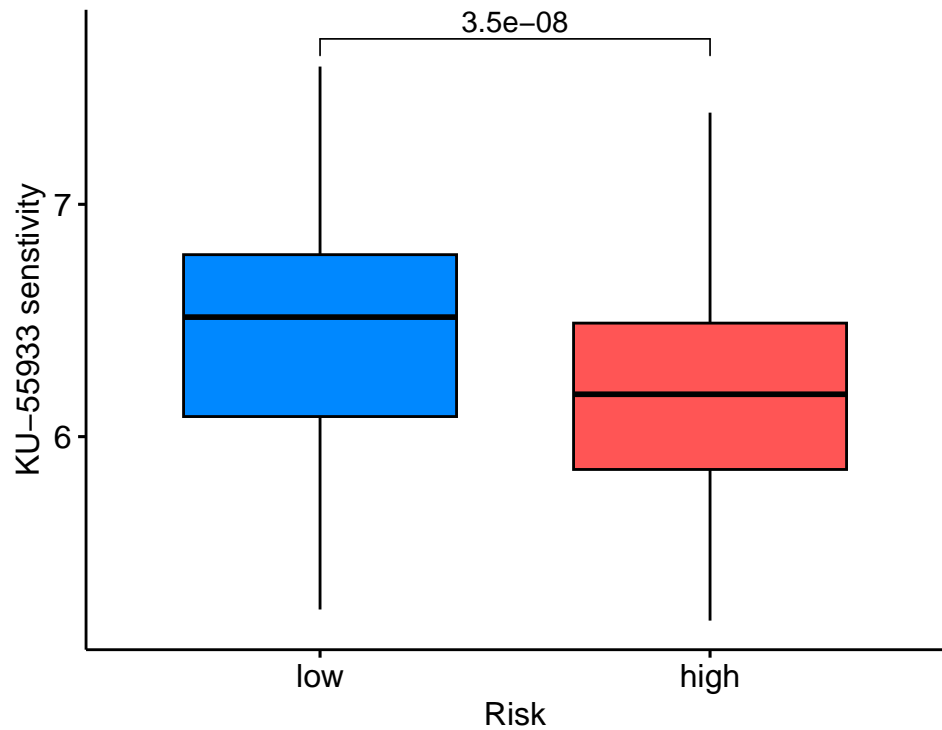

Supplement: Supplementary file 1 — Additional file 1. The different drug sensitivity between high and low-risk groups in TCGA cohort. [file 40001_2024_1642_MOESM1_ESM.zip › Supplementary material/supplementary file 1/drugSenstivity.KU-55933.pdf]

Risk low high

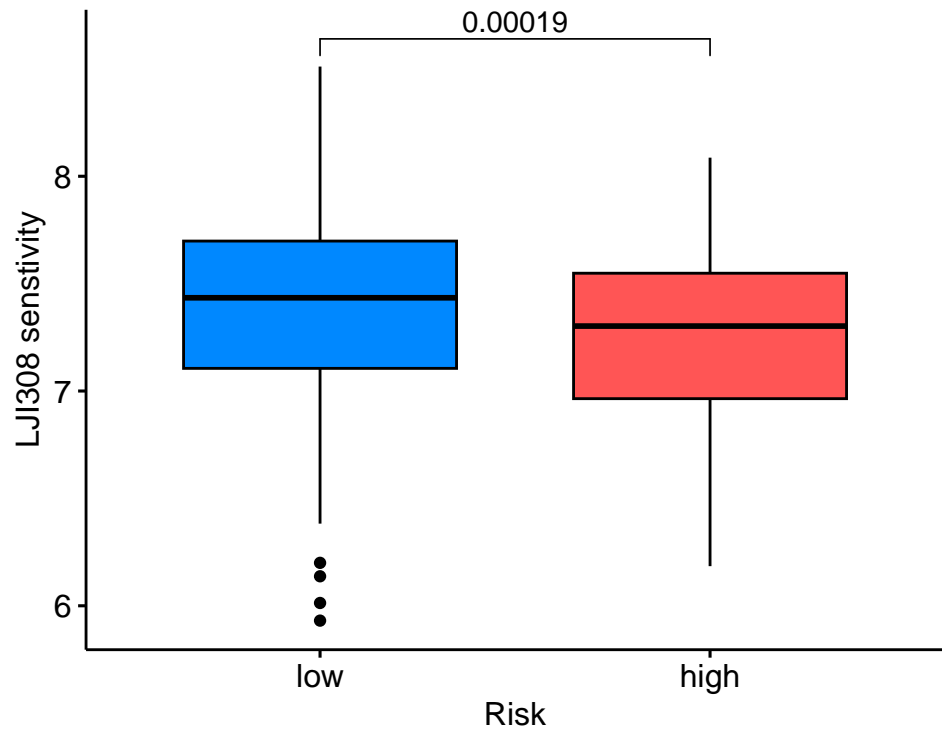

Supplement: Supplementary file 1 — Additional file 1. The different drug sensitivity between high and low-risk groups in TCGA cohort. [file 40001_2024_1642_MOESM1_ESM.zip › Supplementary material/supplementary file 1/drugSenstivity.LJI308.pdf]

Risk 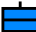 low 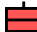 high

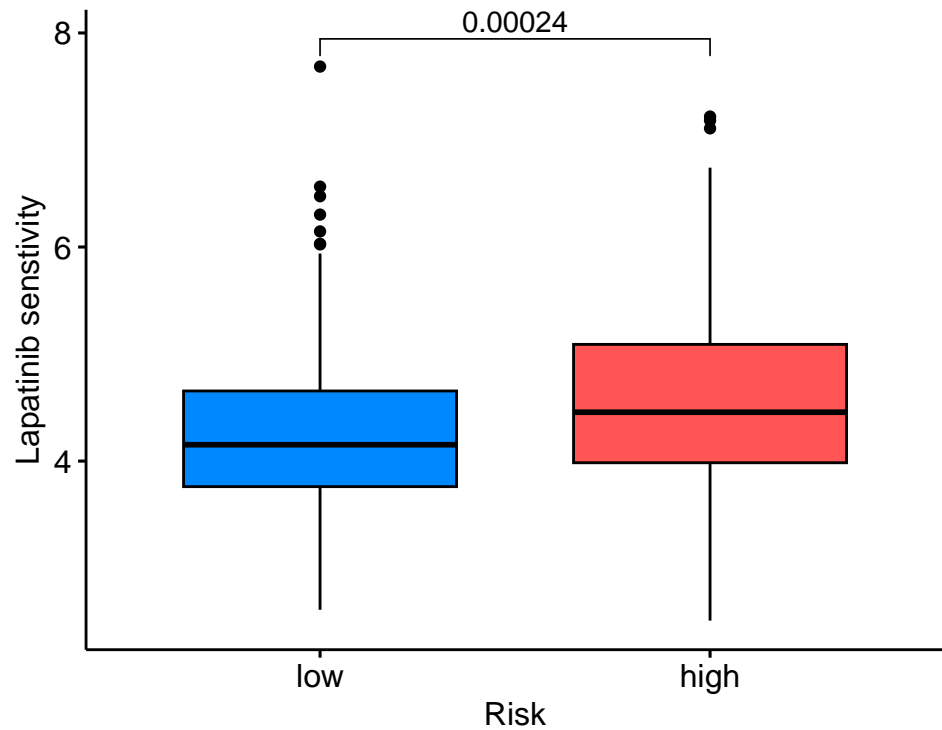

Supplement: Supplementary file 1 — Additional file 1. The different drug sensitivity between high and low-risk groups in TCGA cohort. [file 40001_2024_1642_MOESM1_ESM.zip › Supplementary material/supplementary file 1/drugSenstivity.Lapatinib.pdf]

Risk low high

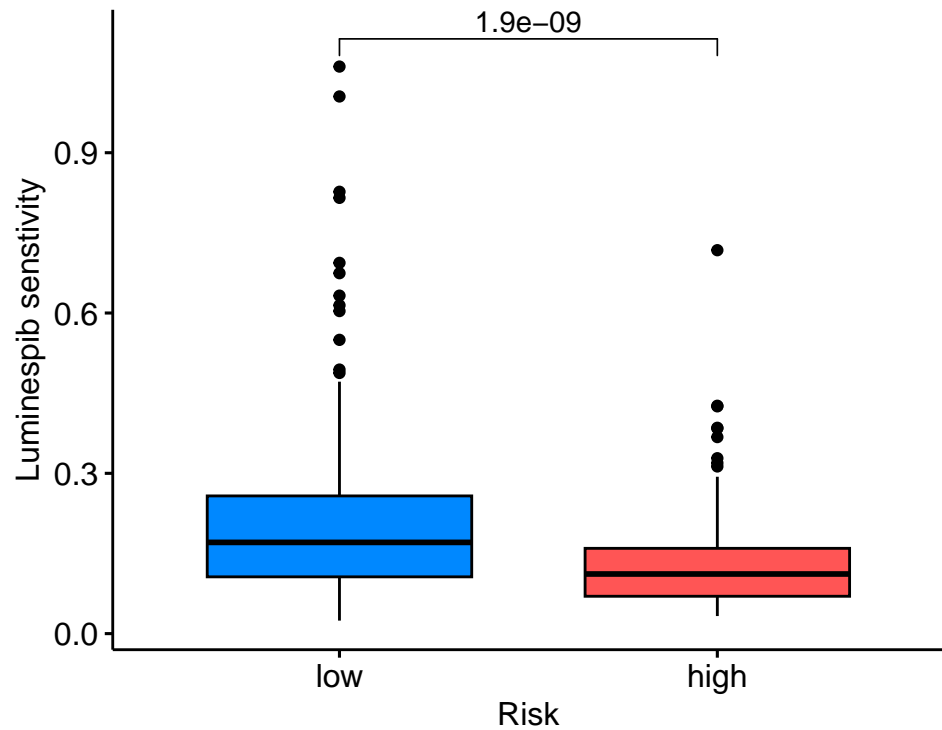

Supplement: Supplementary file 1 — Additional file 1. The different drug sensitivity between high and low-risk groups in TCGA cohort. [file 40001_2024_1642_MOESM1_ESM.zip › Supplementary material/supplementary file 1/drugSenstivity.Luminespib.pdf]

Risk 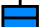 low 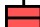 high

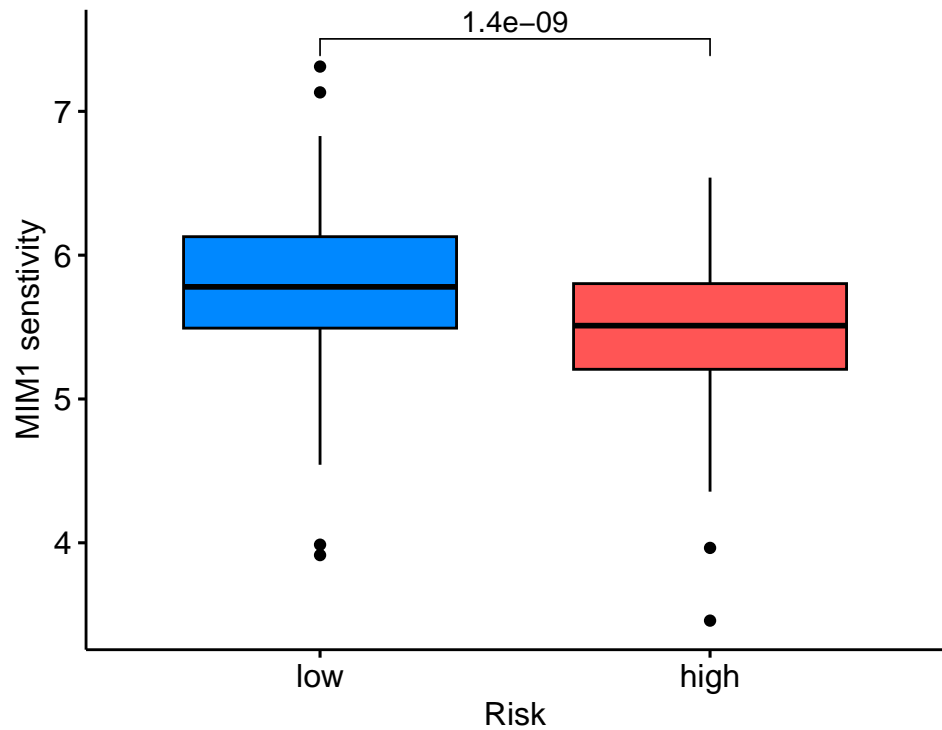

Supplement: Supplementary file 1 — Additional file 1. The different drug sensitivity between high and low-risk groups in TCGA cohort. [file 40001_2024_1642_MOESM1_ESM.zip › Supplementary material/supplementary file 1/drugSenstivity.MIM1.pdf]

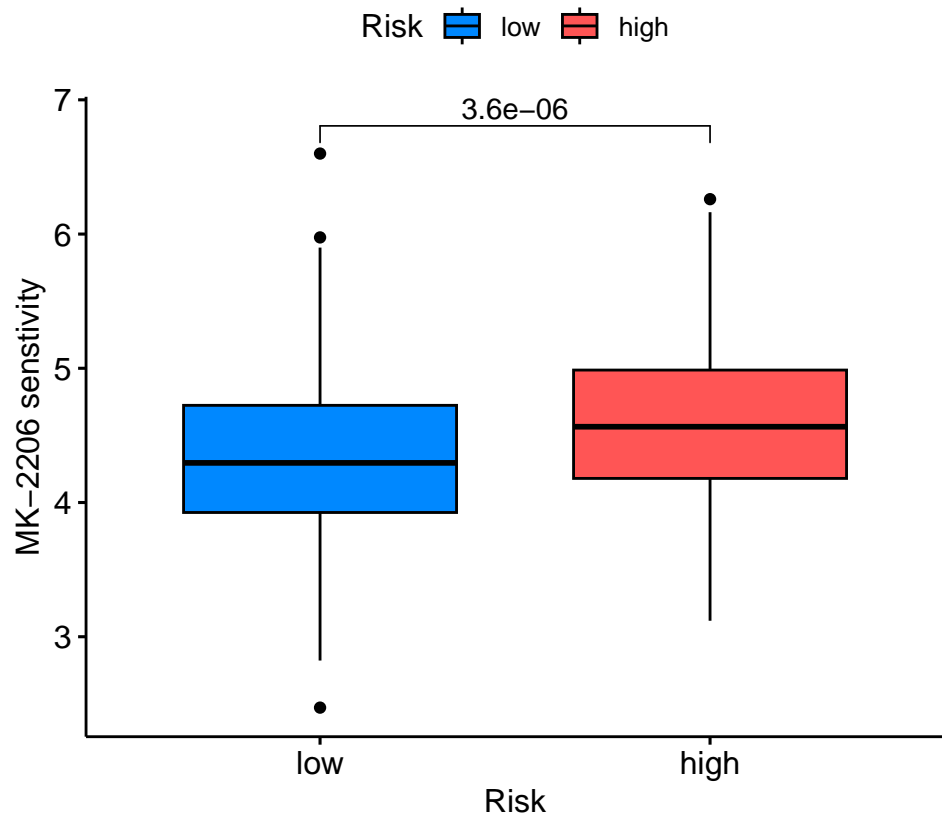

Supplement: Supplementary file 1 — Additional file 1. The different drug sensitivity between high and low-risk groups in TCGA cohort. [file 40001_2024_1642_MOESM1_ESM.zip › Supplementary material/supplementary file 1/drugSenstivity.MK-2206.pdf]

Risk 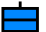 low 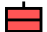 high

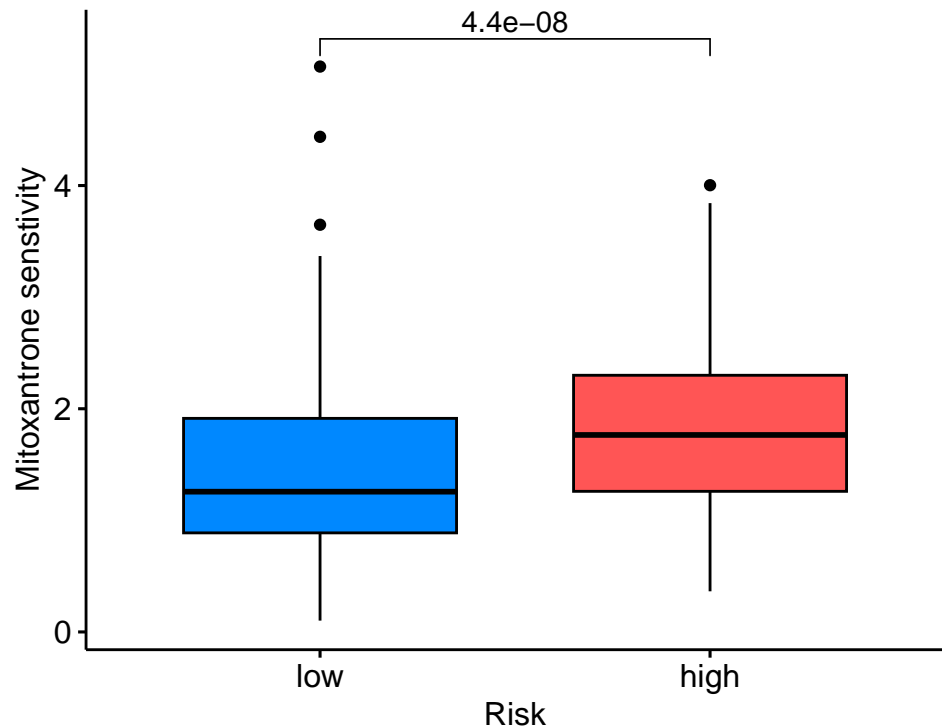

Supplement: Supplementary file 1 — Additional file 1. The different drug sensitivity between high and low-risk groups in TCGA cohort. [file 40001_2024_1642_MOESM1_ESM.zip › Supplementary material/supplementary file 1/drugSenstivity.Mitoxantrone.pdf]

Risk 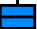 low 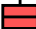 high

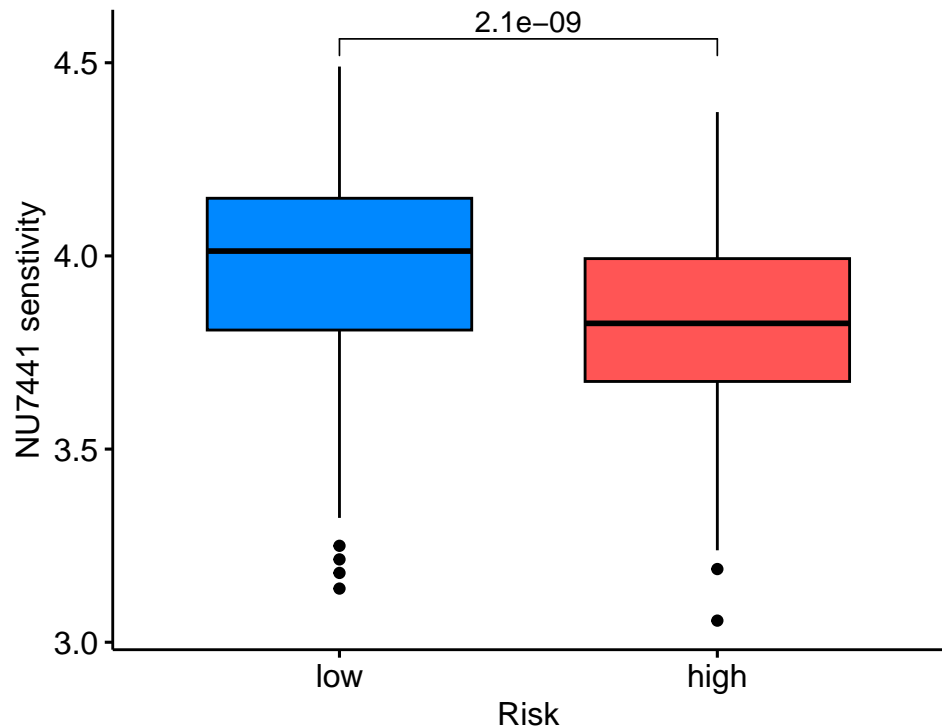

Supplement: Supplementary file 1 — Additional file 1. The different drug sensitivity between high and low-risk groups in TCGA cohort. [file 40001_2024_1642_MOESM1_ESM.zip › Supplementary material/supplementary file 1/drugSenstivity.NU7441.pdf]

Risk 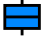 low 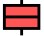 high

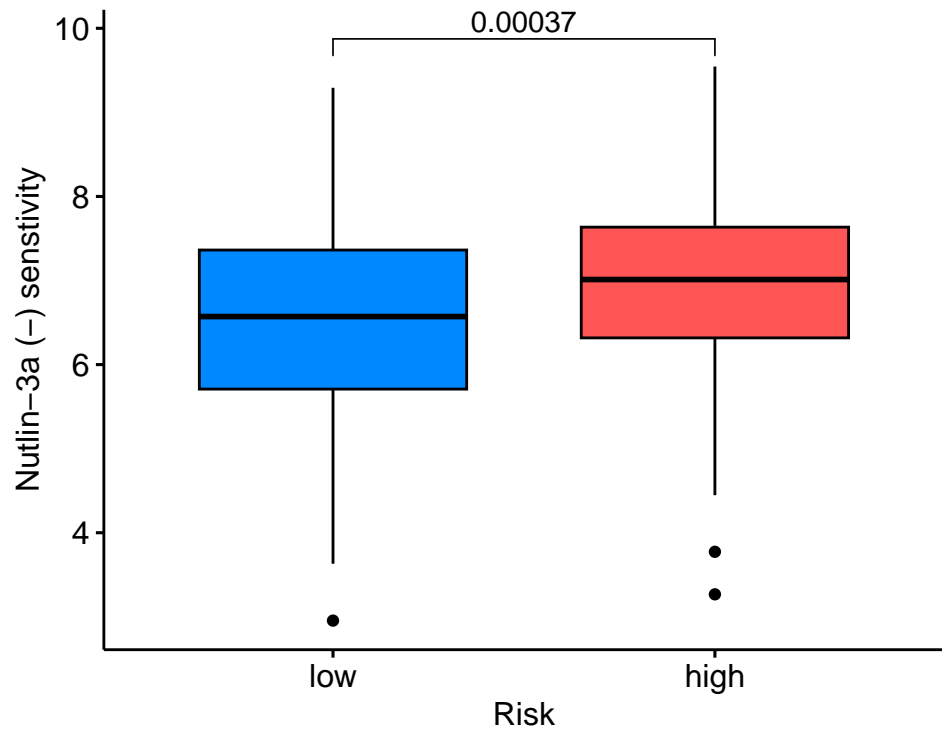

Supplement: Supplementary file 1 — Additional file 1. The different drug sensitivity between high and low-risk groups in TCGA cohort. [file 40001_2024_1642_MOESM1_ESM.zip › Supplementary material/supplementary file 1/drugSenstivity.Nutlin-3a (-).pdf]

Risk 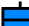 low 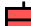 high

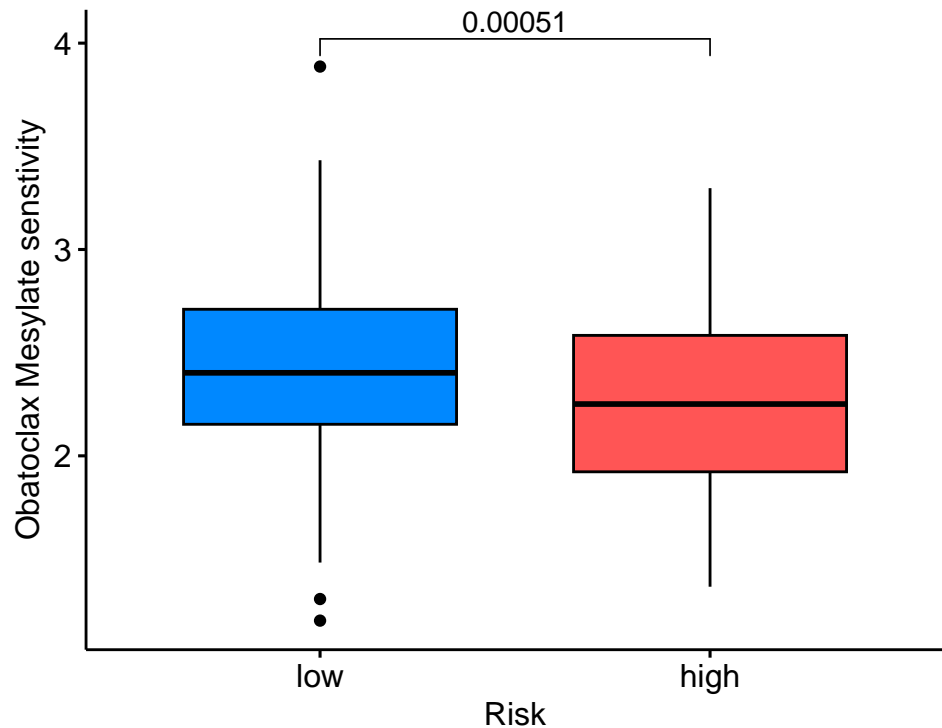

Supplement: Supplementary file 1 — Additional file 1. The different drug sensitivity between high and low-risk groups in TCGA cohort. [file 40001_2024_1642_MOESM1_ESM.zip › Supplementary material/supplementary file 1/drugSenstivity.Obatoclax Mesylate.pdf]

Risk low high

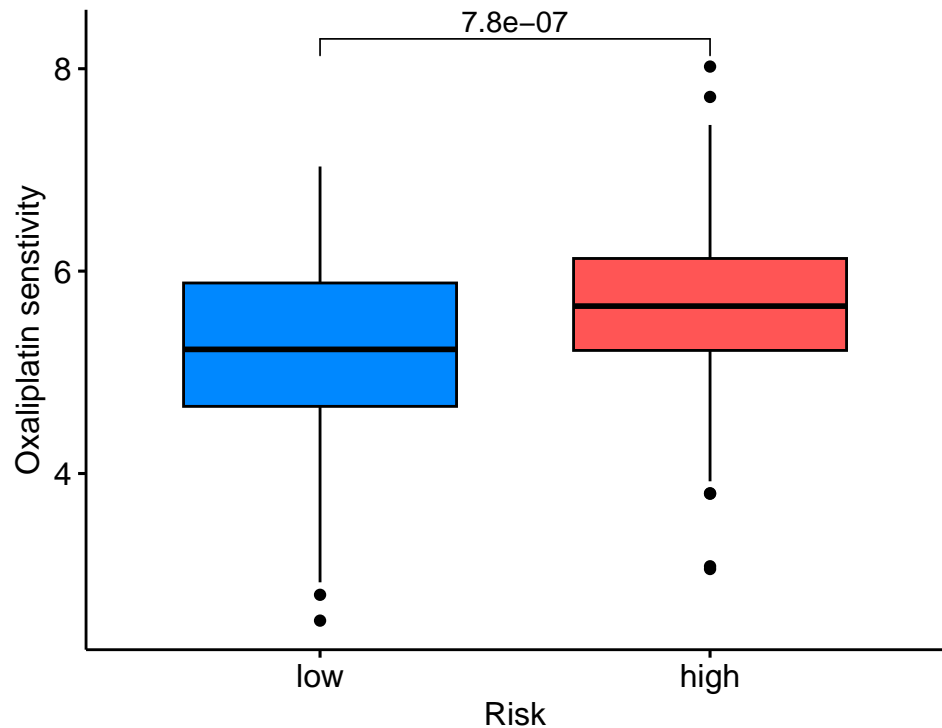

Supplement: Supplementary file 1 — Additional file 1. The different drug sensitivity between high and low-risk groups in TCGA cohort. [file 40001_2024_1642_MOESM1_ESM.zip › Supplementary material/supplementary file 1/drugSenstivity.Oxaliplatin.pdf]

Risk 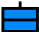 low 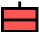 high

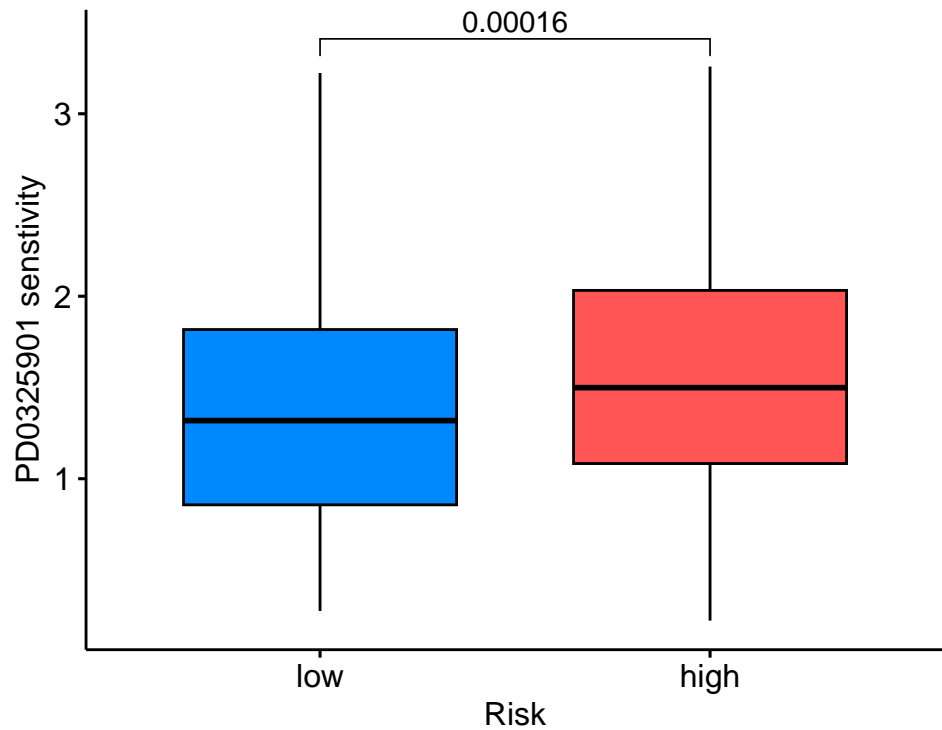

Supplement: Supplementary file 1 — Additional file 1. The different drug sensitivity between high and low-risk groups in TCGA cohort. [file 40001_2024_1642_MOESM1_ESM.zip › Supplementary material/supplementary file 1/drugSenstivity.PD0325901.pdf]

Risk 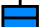 low 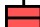 high

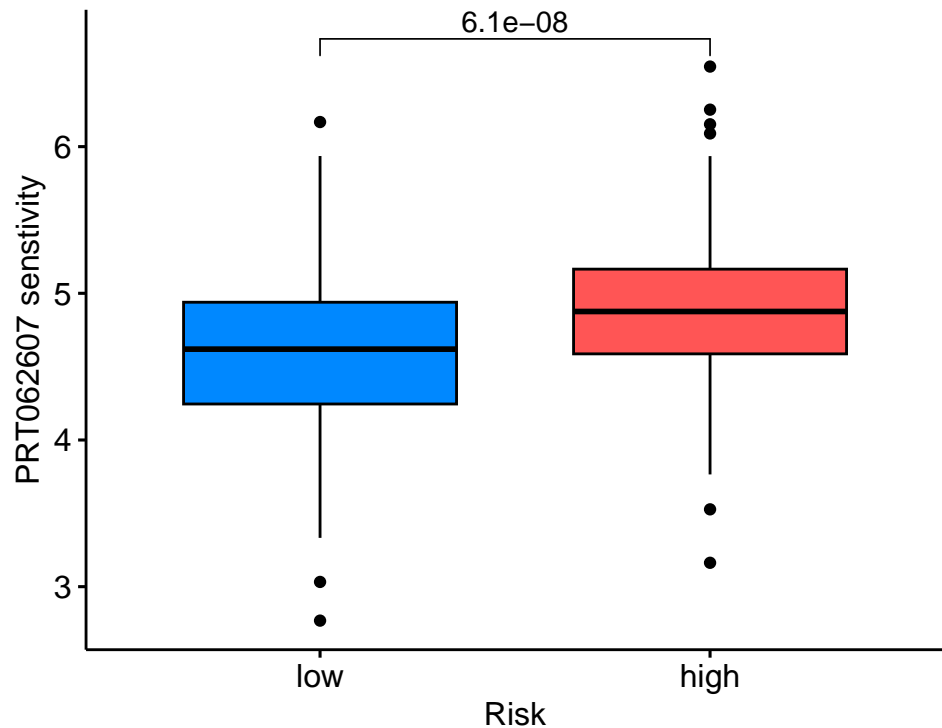

Supplement: Supplementary file 1 — Additional file 1. The different drug sensitivity between high and low-risk groups in TCGA cohort. [file 40001_2024_1642_MOESM1_ESM.zip › Supplementary material/supplementary file 1/drugSenstivity.PRT062607.pdf]

Risk 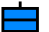 low 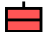 high

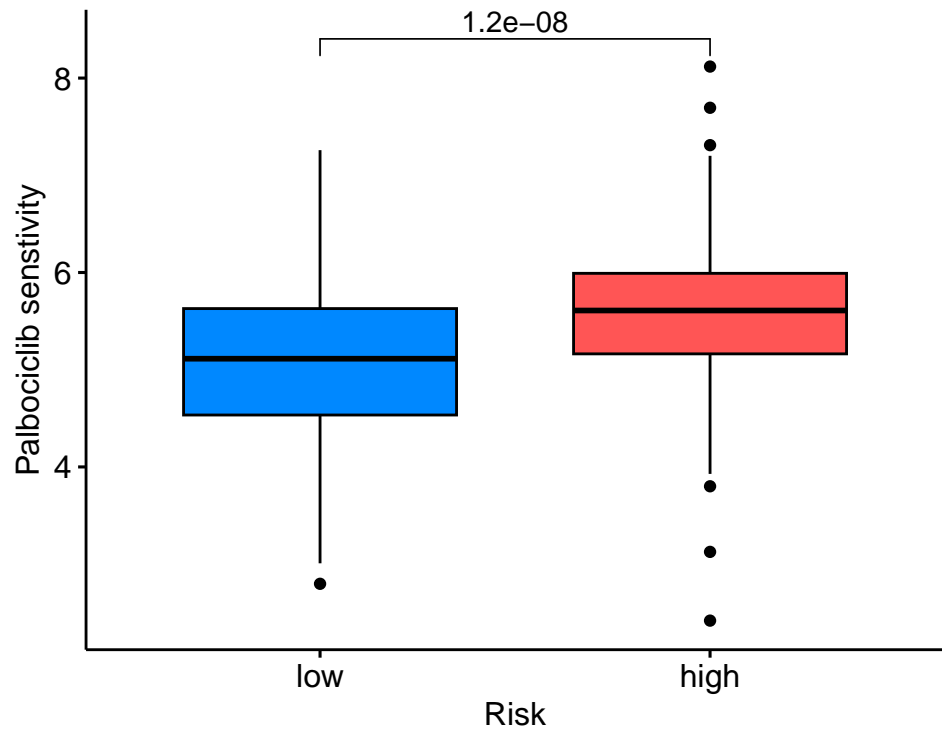

Supplement: Supplementary file 1 — Additional file 1. The different drug sensitivity between high and low-risk groups in TCGA cohort. [file 40001_2024_1642_MOESM1_ESM.zip › Supplementary material/supplementary file 1/drugSenstivity.Palbociclib.pdf]

Risk 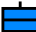 low 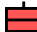 high

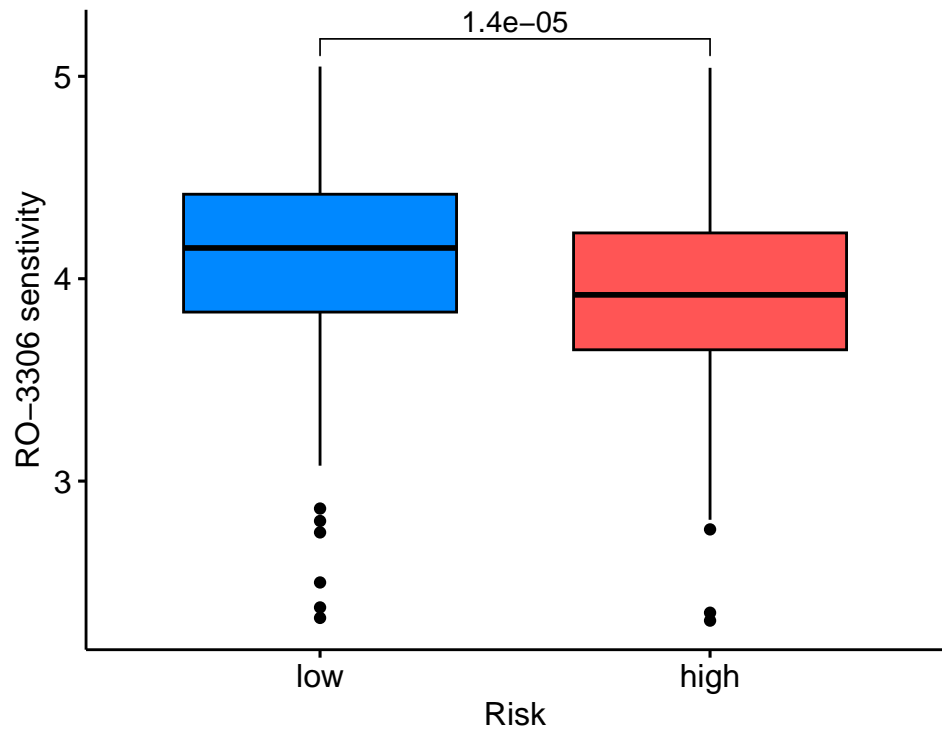

Supplement: Supplementary file 1 — Additional file 1. The different drug sensitivity between high and low-risk groups in TCGA cohort. [file 40001_2024_1642_MOESM1_ESM.zip › Supplementary material/supplementary file 1/drugSenstivity.RO-3306.pdf]

Risk 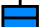 low 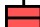 high

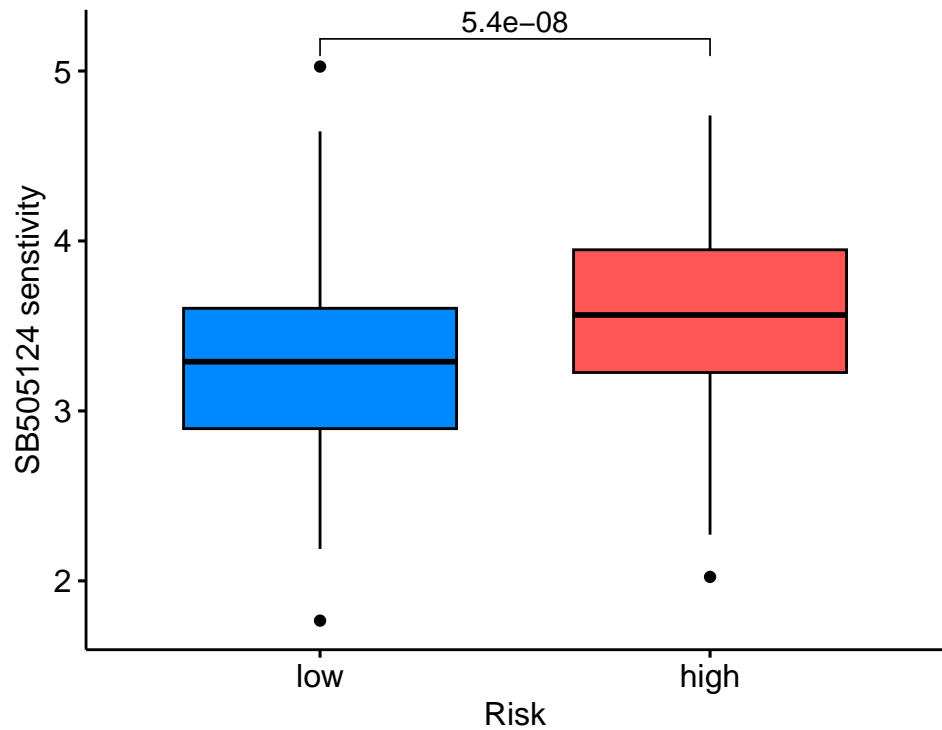

Supplement: Supplementary file 1 — Additional file 1. The different drug sensitivity between high and low-risk groups in TCGA cohort. [file 40001_2024_1642_MOESM1_ESM.zip › Supplementary material/supplementary file 1/drugSenstivity.SB505124.pdf]

Risk 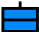 low 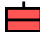 high

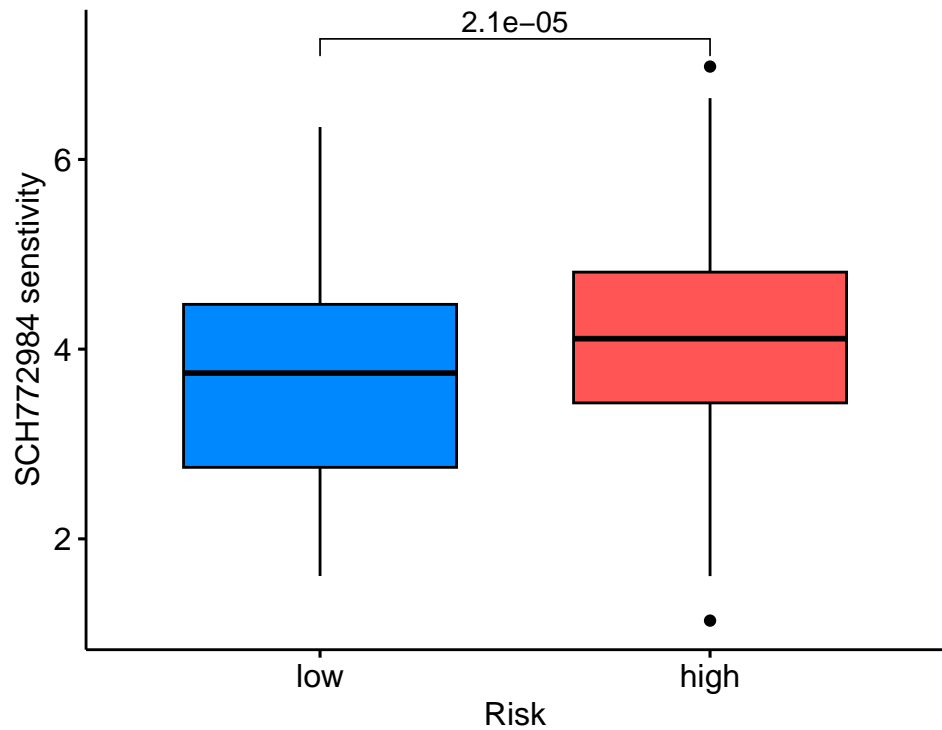

Supplement: Supplementary file 1 — Additional file 1. The different drug sensitivity between high and low-risk groups in TCGA cohort. [file 40001_2024_1642_MOESM1_ESM.zip › Supplementary material/supplementary file 1/drugSenstivity.SCH772984.pdf]

Risk 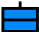 low 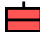 high

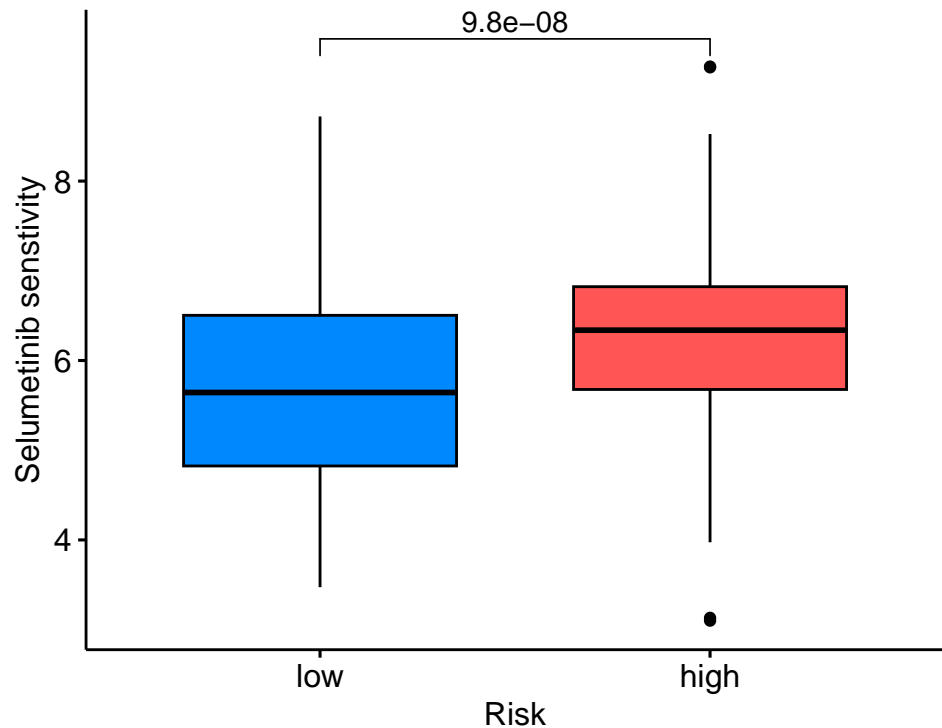

Supplement: Supplementary file 1 — Additional file 1. The different drug sensitivity between high and low-risk groups in TCGA cohort. [file 40001_2024_1642_MOESM1_ESM.zip › Supplementary material/supplementary file 1/drugSenstivity.Selumetinib.pdf]

Risk 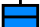 low 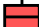 high

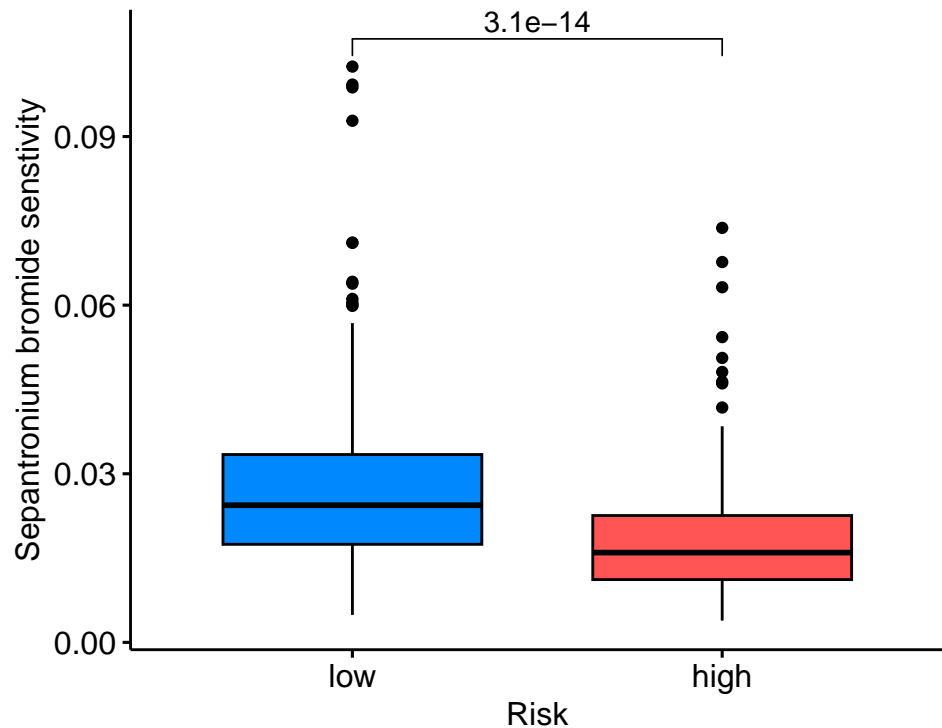

Supplement: Supplementary file 1 — Additional file 1. The different drug sensitivity between high and low-risk groups in TCGA cohort. [file 40001_2024_1642_MOESM1_ESM.zip › Supplementary material/supplementary file 1/drugSenstivity.Sepantronium bromide.pdf]

Risk low high

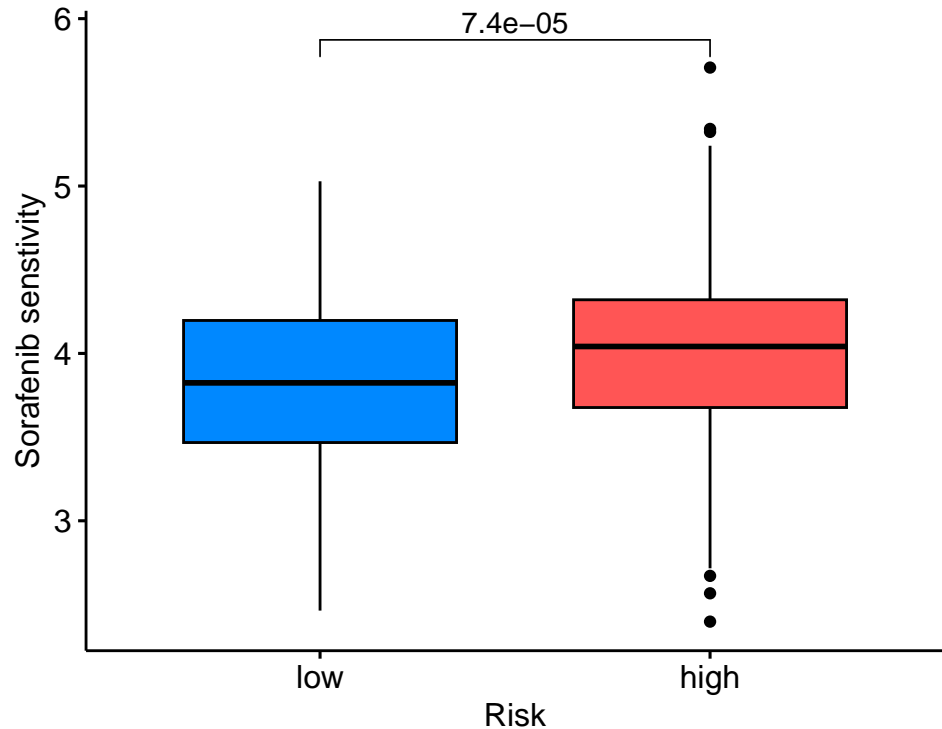

Supplement: Supplementary file 1 — Additional file 1. The different drug sensitivity between high and low-risk groups in TCGA cohort. [file 40001_2024_1642_MOESM1_ESM.zip › Supplementary material/supplementary file 1/drugSenstivity.Sorafenib.pdf]

Risk 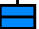 low 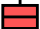 high

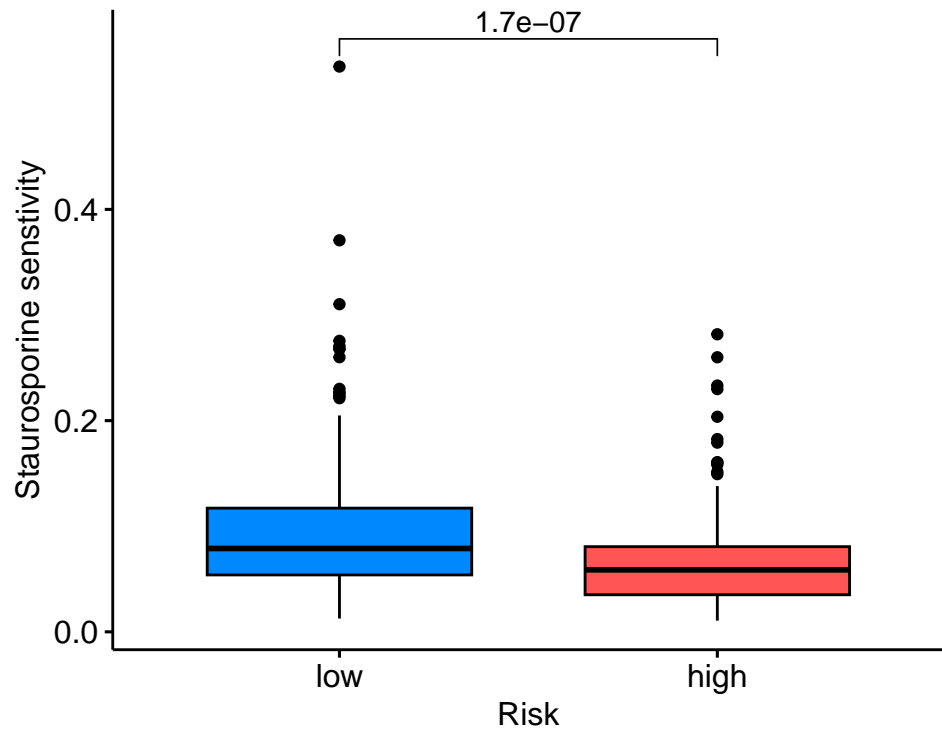

Supplement: Supplementary file 1 — Additional file 1. The different drug sensitivity between high and low-risk groups in TCGA cohort. [file 40001_2024_1642_MOESM1_ESM.zip › Supplementary material/supplementary file 1/drugSenstivity.Staurosporine.pdf]

Risk low high

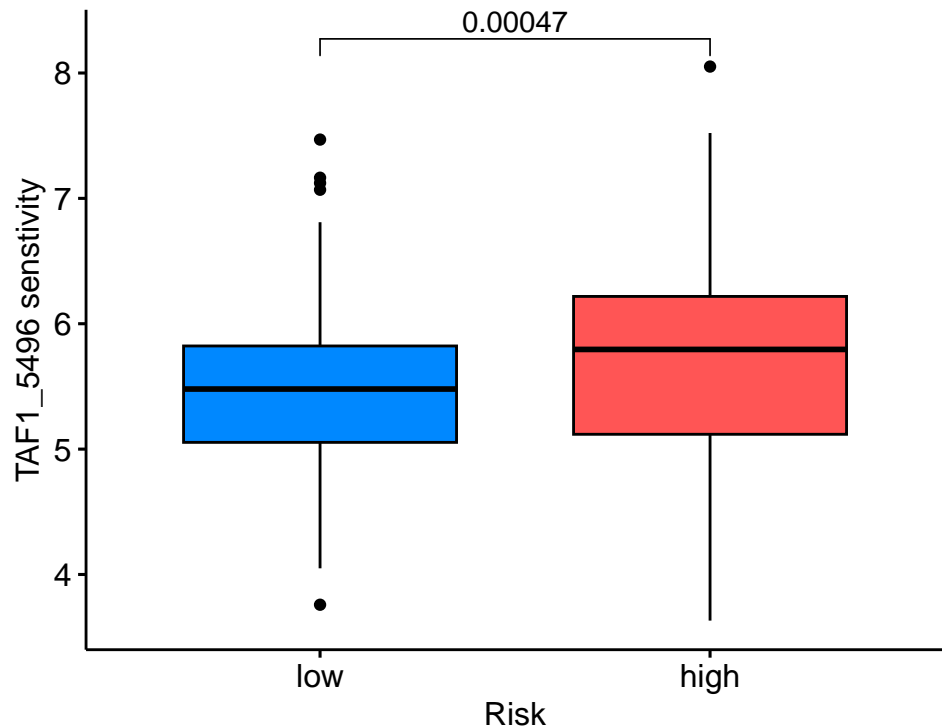

Supplement: Supplementary file 1 — Additional file 1. The different drug sensitivity between high and low-risk groups in TCGA cohort. [file 40001_2024_1642_MOESM1_ESM.zip › Supplementary material/supplementary file 1/drugSenstivity.TAF1_5496.pdf]

Risk 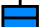 low 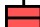 high

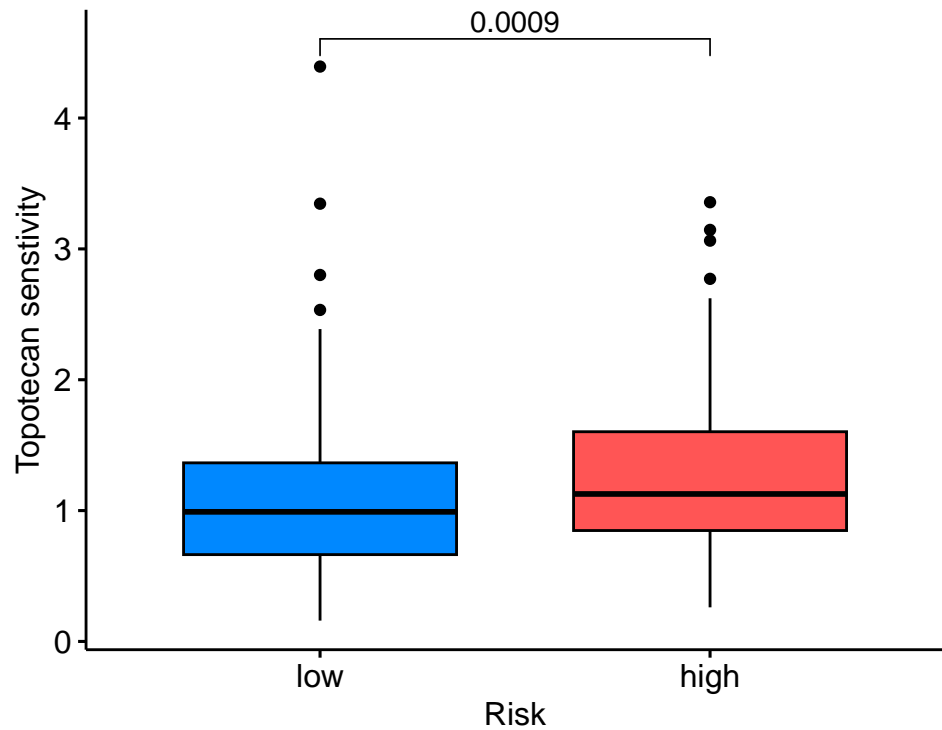

Supplement: Supplementary file 1 — Additional file 1. The different drug sensitivity between high and low-risk groups in TCGA cohort. [file 40001_2024_1642_MOESM1_ESM.zip › Supplementary material/supplementary file 1/drugSenstivity.Topotecan.pdf]

Risk low high

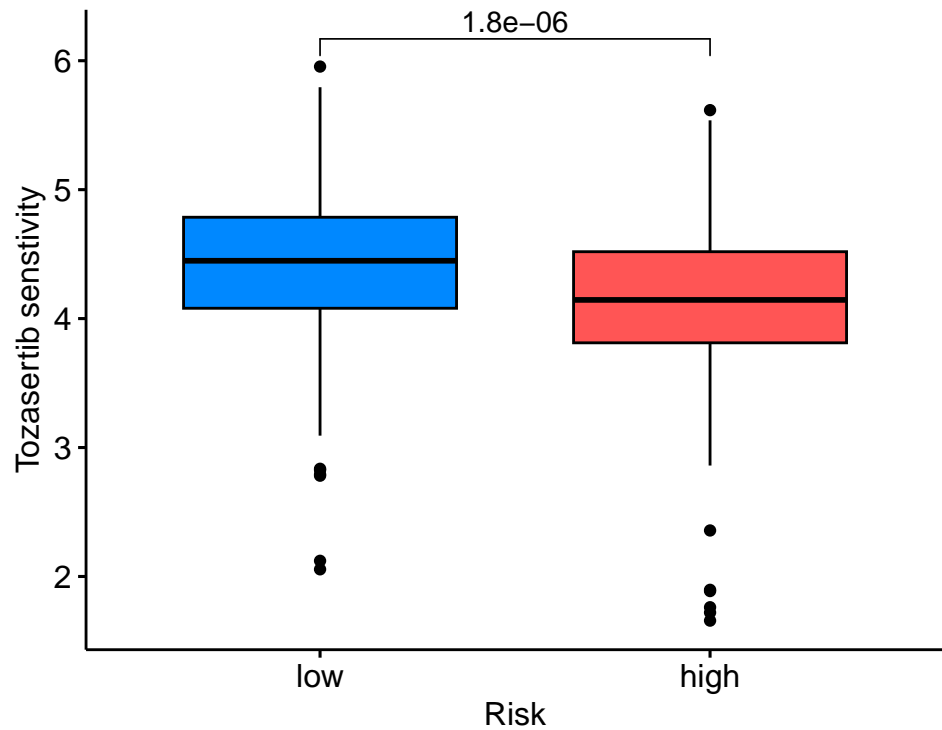

Supplement: Supplementary file 1 — Additional file 1. The different drug sensitivity between high and low-risk groups in TCGA cohort. [file 40001_2024_1642_MOESM1_ESM.zip › Supplementary material/supplementary file 1/drugSenstivity.Tozasertib.pdf]

Risk 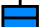 low 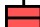 high

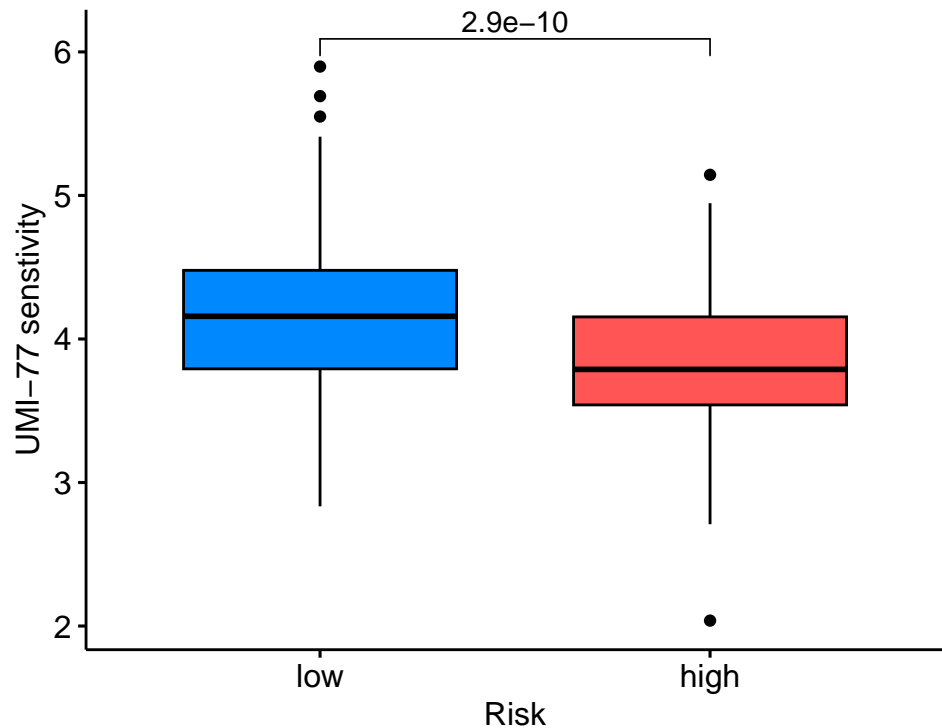

Supplement: Supplementary file 1 — Additional file 1. The different drug sensitivity between high and low-risk groups in TCGA cohort. [file 40001_2024_1642_MOESM1_ESM.zip › Supplementary material/supplementary file 1/drugSenstivity.UMI-77.pdf]

Risk 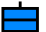 low 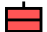 high

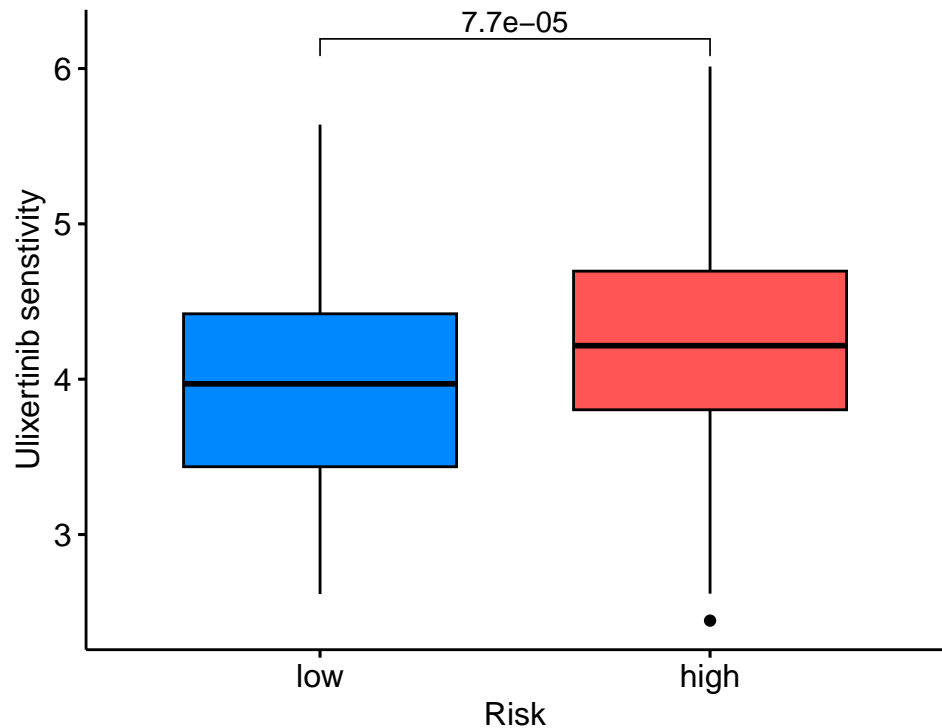

Supplement: Supplementary file 1 — Additional file 1. The different drug sensitivity between high and low-risk groups in TCGA cohort. [file 40001_2024_1642_MOESM1_ESM.zip › Supplementary material/supplementary file 1/drugSenstivity.Ulixertinib.pdf]

Risk 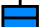 low 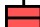 high

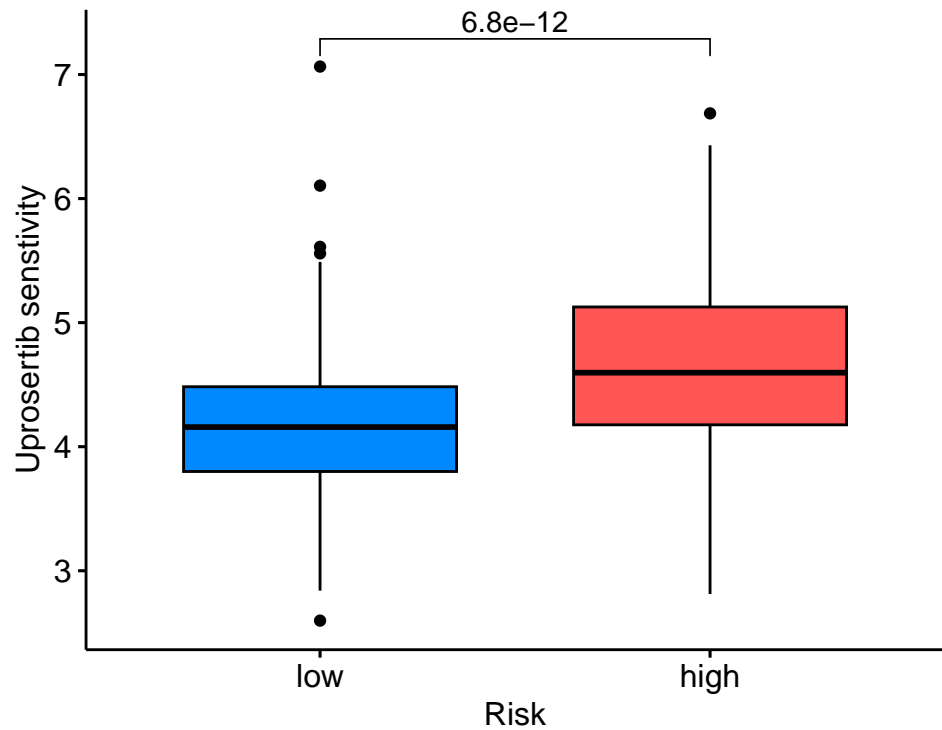

Supplement: Supplementary file 1 — Additional file 1. The different drug sensitivity between high and low-risk groups in TCGA cohort. [file 40001_2024_1642_MOESM1_ESM.zip › Supplementary material/supplementary file 1/drugSenstivity.Uprosertib.pdf]

Risk 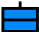 low 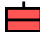 high

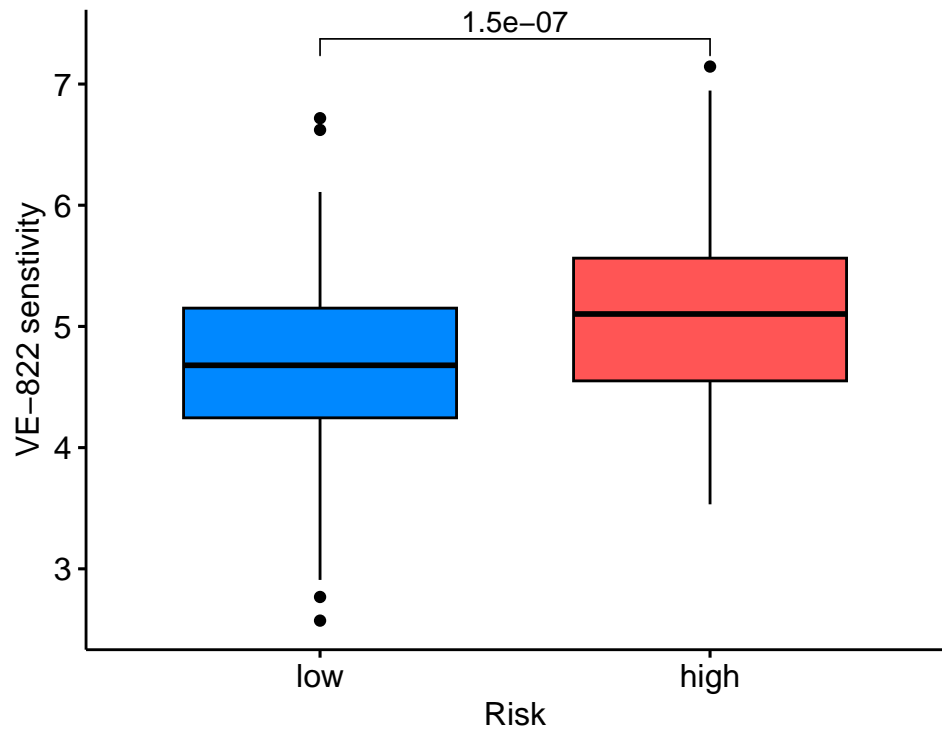

Supplement: Supplementary file 1 — Additional file 1. The different drug sensitivity between high and low-risk groups in TCGA cohort. [file 40001_2024_1642_MOESM1_ESM.zip › Supplementary material/supplementary file 1/drugSenstivity.VE-822.pdf]

Risk 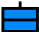 low 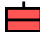 high

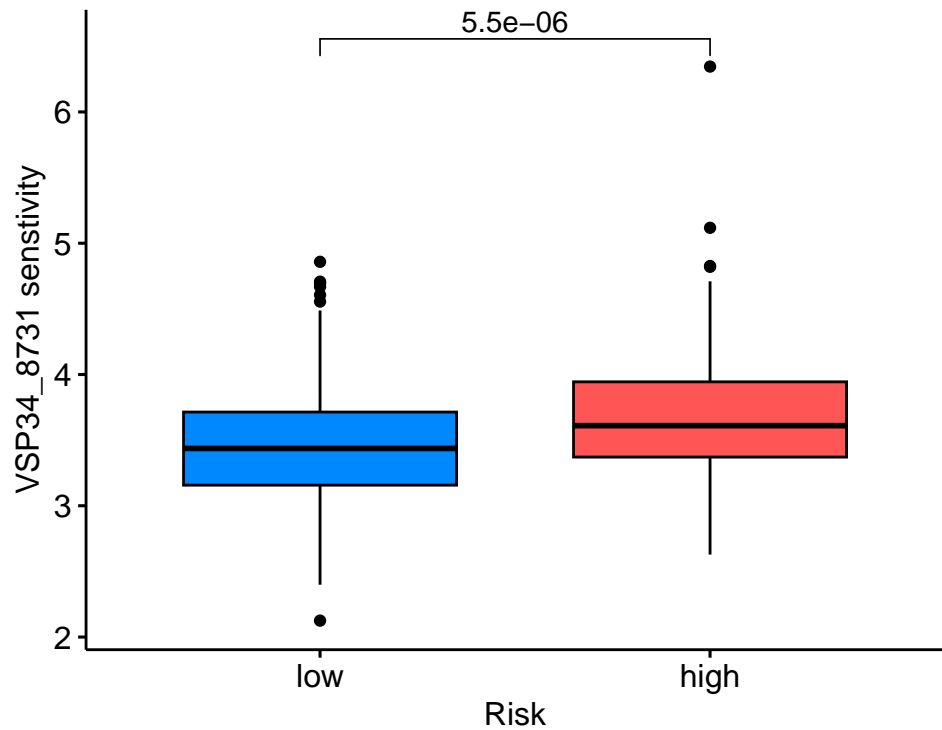

Supplement: Supplementary file 1 — Additional file 1. The different drug sensitivity between high and low-risk groups in TCGA cohort. [file 40001_2024_1642_MOESM1_ESM.zip › Supplementary material/supplementary file 1/drugSenstivity.VSP34_8731.pdf]

Risk 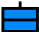 low 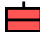 high

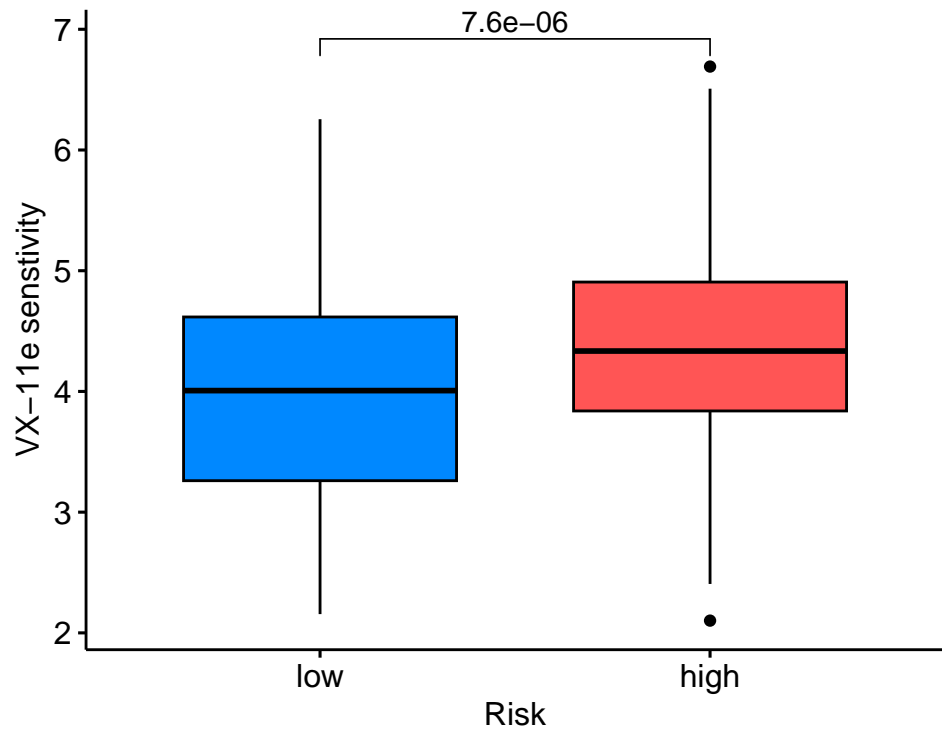

Supplement: Supplementary file 1 — Additional file 1. The different drug sensitivity between high and low-risk groups in TCGA cohort. [file 40001_2024_1642_MOESM1_ESM.zip › Supplementary material/supplementary file 1/drugSenstivity.VX-11e.pdf]

Risk low high

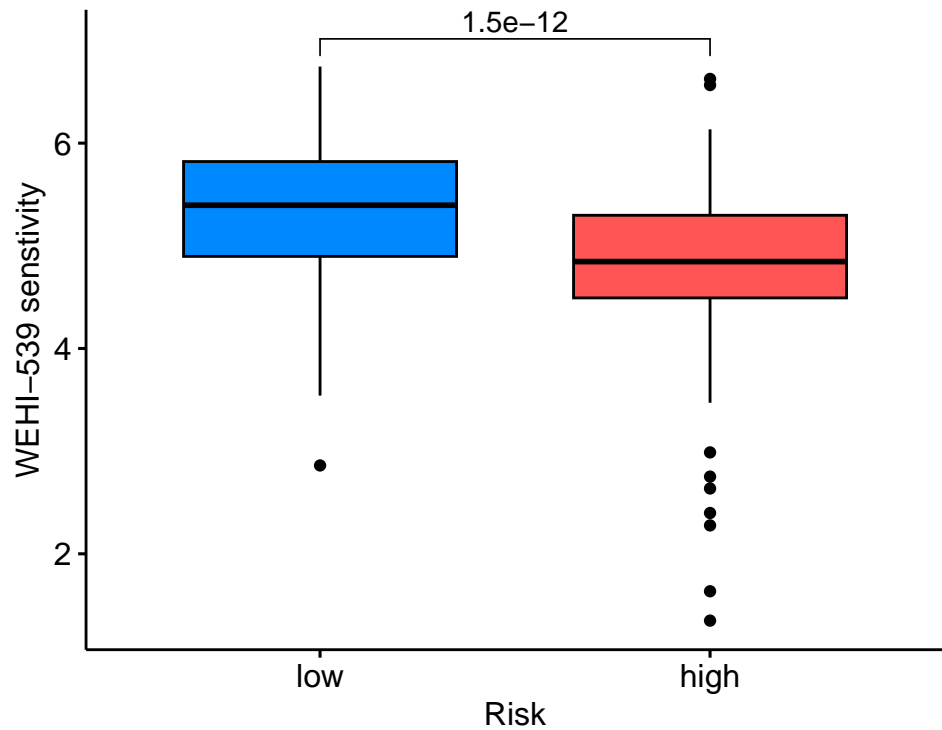

Supplement: Supplementary file 1 — Additional file 1. The different drug sensitivity between high and low-risk groups in TCGA cohort. [file 40001_2024_1642_MOESM1_ESM.zip › Supplementary material/supplementary file 1/drugSenstivity.WEHI-539.pdf]

Risk 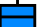 low 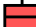 high

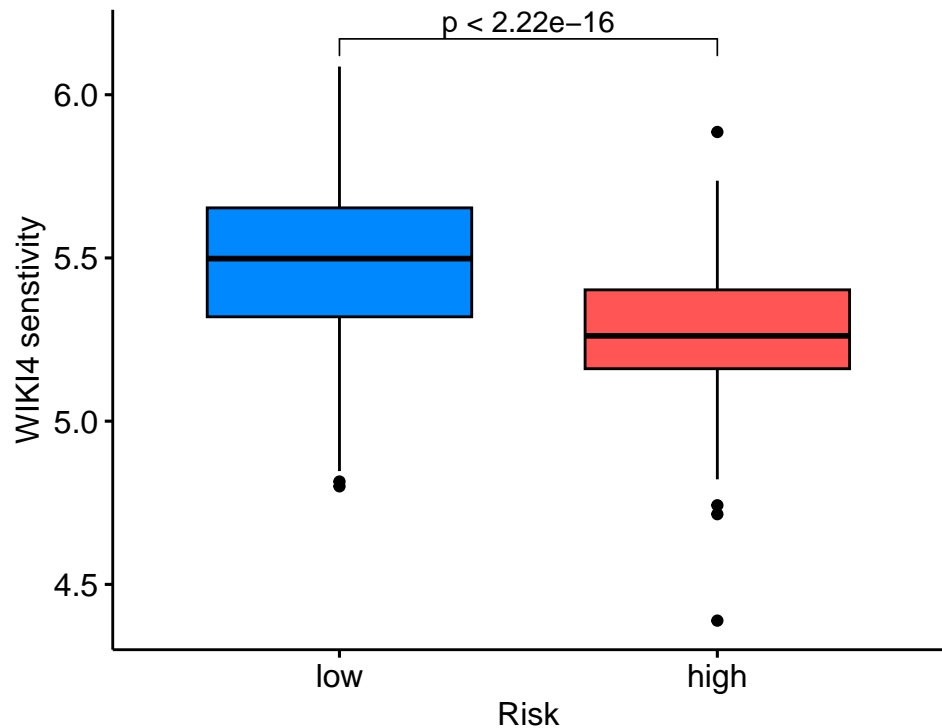

Supplement: Supplementary file 1 — Additional file 1. The different drug sensitivity between high and low-risk groups in TCGA cohort. [file 40001_2024_1642_MOESM1_ESM.zip › Supplementary material/supplementary file 1/drugSenstivity.WIKI4.pdf]

Risk low high

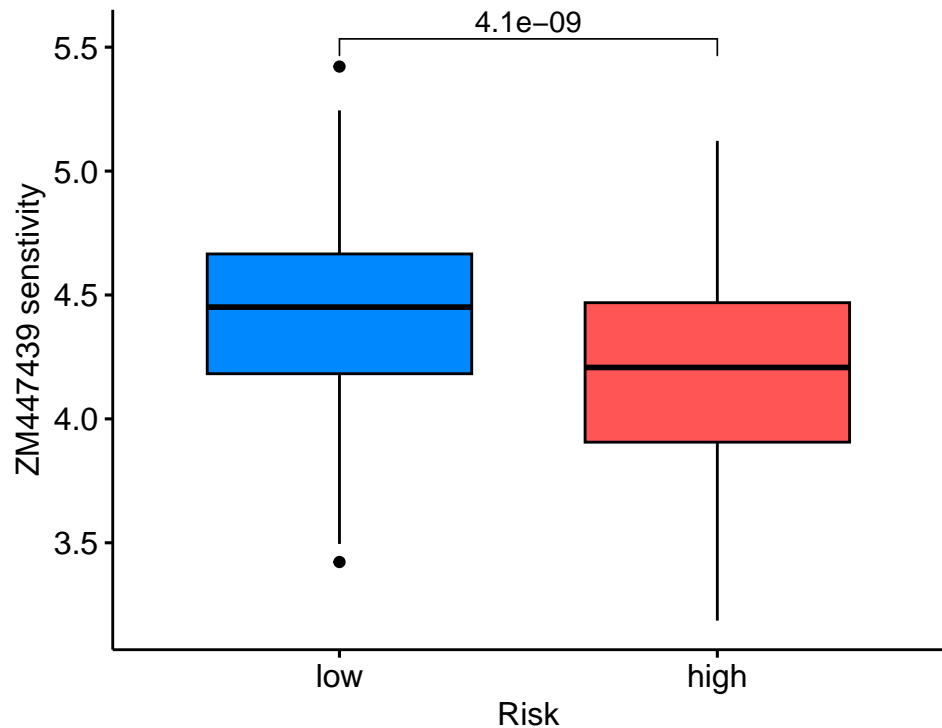

Supplement: Supplementary file 1 — Additional file 1. The different drug sensitivity between high and low-risk groups in TCGA cohort. [file 40001_2024_1642_MOESM1_ESM.zip › Supplementary material/supplementary file 1/drugSenstivity.ZM447439.pdf]
